# Sex-Specific Effects of Nutritional Supplements for Infants Born Early or Small: An Individual Participant Data Meta-Analysis (ESSENCE IPD-MA) I—Cognitive Function and Metabolic Risk

**DOI:** 10.3390/nu14030418

**Published:** 2022-01-18

**Authors:** Luling Lin, Greg D. Gamble, Caroline A. Crowther, Frank H. Bloomfield, Massimo Agosti, Stephanie A. Atkinson, Augusto Biasini, Nicholas D. Embleton, Mary S. Fewtrell, Fernando Lamy-Filho, Christoph Fusch, Maria L. Gianni, H. Gozde Kanmaz Kutman, Winston Koo, Ita Litmanovitz, Colin Morgan, Kanya Mukhopadhyay, Erica Neri, Jean-Charles Picaud, Niels Rochow, Paola Roggero, Atul Singhal, Kenneth Stroemmen, Maw J. Tan, Francesco M. Tandoi, Claire L. Wood, Gitte Zachariassen, Jane E. Harding

**Affiliations:** 1 Liggins Institute, University of Auckland, Auckland 1023, New Zealand; luling.lin@auckland.ac.nz (L.L.); gd.gamble@auckland.ac.nz (G.D.G.); c.crowther@auckland.ac.nz (C.A.C.); f.bloomfield@auckland.ac.nz (F.H.B.); 2 Neonatal Intensive Care Unit, Woman and Child Department, Ospedale Del Ponte, Insubria University, 21100 Varese, Italy; massimo.agosti@asst-settelaghi.it (M.A.); Francesco.Tandoi@asst-settelaghi.it (F.M.T.); 3 Department of Pediatrics, Faculty of Health Sciences, McMaster University, Hamilton, ON L8N 3Z5, Canada; satkins@mcmaster.ca (S.A.A.); Christoph.Fusch@klinikum-nuernberg.de (C.F.); nielsrochow@gmail.com (N.R.); 4 Donor Human Milk Bank Italian Association (AIBLUD), 20126 Milan, Italy; augustoclimb@gmail.com; 5 Population Health Sciences Institute, Newcastle University, Newcastle upon Tyne NE2 4AX, UK; nicholas.embleton@newcastle.ac.uk; 6 Childhood Nutrition Research Centre, UCL Great Ormond Street Institute of Child Health, London WC1N 1EH, UK; m.fewtrell@ucl.ac.uk; 7 Departamento de Medicina, Universidade Federal do Maranhão (UFMA), São Luís 65080-805, MA, Brazil; lamyfilho@gmail.com; 8 Department of Pediatrics, Nuremberg General Hospital, Paracelsus Medical University, 90471 Nuremberg, Germany; 9 Fondazione IRCCS Cà Granda Ospedale Maggiore Policlinico, Via Commenda 12, 20122 Milan, Italy; maria.gianni@unimi.it (M.L.G.); paola.roggero@unimi.it (P.R.); 10 Department of Clinical Sciences and Community Health, University of Milan, Via Commenda 19, 20122 Milan, Italy; 11 Department of Neonatology, Bilkent City Hospital, Ankara 06800, Turkey; gzdekanmaz@yahoo.com; 12 Department of Nutrition and Food Science, Wayne State University, Detroit, MI 48202, USA; aa3796@wayne.edu; 13 Department of Neonatology, Meir Medical Center, Kfar Saba 44281, Israel; litmani@clalit.org.il; 14 Department of Neonatology, Liverpool Women’s Hospital, Liverpool L8 7SS, UK; colin.morgan@lwh.nhs.uk; 15 Department of Pediatrics, Post Graduate Institute of Medical Education and Research (PGIMER), Chandigarh 160012, India; kanyapgi@gmail.com; 16 Department of Psychology, University of Bologna, 40100 Bologna, Italy; erica.neri4@unibo.it; 17 Division of Neonatology, Hôpital de la Croix-Rousse, Hospices Civils de Lyon, 69004 Lyon, France; jean-charles.picaud@chu-lyon.fr; 18 CarMen Laboratory, INSERM, INRA, Claude Bernard University Lyon 1, 69677 Pierre Benite, France; 19 Department of Nutrition, Institute of Child Health, London WC1N 1EH, UK; a.singhal@ucl.ac.uk; 20 Department of Neonatal Intensive Care, Division of Paediatric and Adolescent Medicine, Rikshospitalet, Oslo University Hospital, 0310 Oslo, Norway; kestromm@gmail.com; 21 Department of Developmental Paediatrics, Alder Hey Children’s NHS Foundation Trust, Liverpool L12 2AP, UK; tanyeo001@aol.com; 22 Translational and Clinical Research Institute, Faculty of Medical Sciences, Newcastle University, Newcastle upon Tyne NE1 7RU, UK; claire.wood@newcastle.ac.uk; 23 H.C. Andersen Children’s Hospital, Odense University Hospital and University of Southern Denmark, 5000 Odense, Denmark; gitte.zachariassen@rsyd.dk

**Keywords:** macronutrient supplementation, preterm infants, small-for gestational-age infants, cognitive function, metabolic risk, individual participants data meta-analysis, systematic review

## Abstract

Neonatal nutritional supplements are widely used to improve growth and development but may increase risk of later metabolic disease, and effects may differ by sex. We assessed effects of supplements on later development and metabolism. We searched databases and clinical trials registers up to April 2019. Participant-level data from randomised trials were included if the intention was to increase macronutrient intake to improve growth or development of infants born preterm or small-for-gestational-age. Co-primary outcomes were cognitive impairment and metabolic risk. Supplementation did not alter cognitive impairment in toddlers (13 trials, *n* = 1410; adjusted relative risk (aRR) 0.88 [95% CI 0.68, 1.13]; *p* = 0.31) or older ages, nor alter metabolic risk beyond 3 years (5 trials, *n* = 438; aRR 0.94 [0.76, 1.17]; *p* = 0.59). However, supplementation reduced motor impairment in toddlers (13 trials, *n* = 1406; aRR 0.76 [0.60, 0.97]; *p* = 0.03), and improved motor scores overall (13 trials, *n* = 1406; adjusted mean difference 1.57 [0.14, 2.99]; *p* = 0.03) and in girls not boys (*p* = 0.03 for interaction). Supplementation lowered triglyceride concentrations but did not affect other metabolic outcomes (high-density and low-density lipoproteins, cholesterol, fasting glucose, blood pressure, body mass index). Macronutrient supplementation for infants born small may not alter later cognitive function or metabolic risk, but may improve early motor function, especially for girls.

## 1. Introduction

As the mortality of infants born preterm or small-for-gestational-age (SGA) has decreased, there is a greater focus on improving their quality of life. Such infants are at increased risk of poor growth, disability and delayed development [1,2,3], and as adults, they are at increased risk of obesity, diabetes and heart disease [4]. Although provision of enhanced early nutritional supplement is reported to improve early growth and cognitive development [5,6,7], findings from observational studies suggest that early rapid growth may also contribute to adiposity, metabolic and cardiovascular diseases in later life [8,9,10].

Further, sexual diversity may exist in both nutrient needs and responses to early nutritional supplementation nutrition [11,12,13]. For example, it was reported that the protein and energy requirements of preterm boys are higher than those for girls [14], and delayed early nutrition is more likely to cause adverse effects in preterm boys [11]. A recent systematic review found that enhanced nutrition improved cognitive outcomes in toddler boys but not girls [12]. However, the long-term and the sex-specific effects of enhanced nutrition have not been adequately explored, with few trials separately analysing outcomes of girls and boys.

To address these limitations, we undertook individual participant data (IPD) meta-analysis (MA) of data from trials reporting post-discharge outcomes after macronutrient supplements for infants born preterm or small, and in particular whether these effects differed between girls and boys.

## 2. Methods

The protocol of the ESSENCE (Sex-Specific Effects of Early Nutritional Supplements in Children Born Early or Small) IPD-MA was published [15]. The study followed the IPD-MA approach [16] registered prospectively in PROSPERO (CRD42017072683) and reported according to the Preferred Reporting Items for Systematic Reviews and Meta-Analyses (PRISMA) guidelines (Text S1).

### 2.1. Search Strategies

The systematic search was conducted from inception to 1 April 2019 using OvidMedline, Embase, Cochrane Library Central Registry of Controlled Trials and Cochrane Database of Systematic Reviews. We also searched for eligible ongoing trials in Current Controlled Trials (www.controlled-trials.com, accessed on 30 January 2021), Clinical Trials (www.clinicaltrials.gov, accessed on 30 January 2021), the Australian and New Zealand Clinical Trials Registry (www.anzctr.org.au, accessed on 30 January 2021) and WHO ICTRP Search Portal (https://apps.who.int/trialsearch/, accessed on 30 January 2021) (Table S9).

### 2.2. Criteria for Inclusion and Exclusion

Published and unpublished randomised and quasi-randomised trials without restrictions on date of publication or language were included. Eligible trials studied infants born preterm (<37 weeks’ gestation) or born SGA (birthweight < 10th centile for gestational age) and the intervention was intended to increase the intake of one or more macronutrients (protein, carbohydrate, fat, energy content or protein to energy ratio), with the primary aim of improving growth and development.

Interventions could be enteral or parenteral or a combination thereof, commence at any time during the hospitalisation or after discharge from hospital and must have been provided for a minimum duration of one week. Trials were eligible if they reported comparisons between unsupplemented nutrition and supplemented nutrition with parenteral supplements, human breast milk supplements, formula milk or other macronutrients. Trials were excluded that examined the timing of the introduction of nutrition (early versus delayed feeding); that compared macronutrients of different composition (e.g., different types of lipids or proteins); whose outcomes focused on gastrointestinal development rather than growth and development and reported on variations in composition of micronutrients (including sodium, potassium, calcium, phosphorus, vitamins, other minerals, amino acids, fatty acids).

Outcome data required reporting beyond term equivalent age (>37 weeks’ postmenstrual age) or following discharge from hospital after birth. Outcomes were categorised and evaluated in infancy (≤1 years), toddlers (1 to 3 years), childhood (4 to 8 years), adolescence (9 to 18 years) and adulthood (>18 years).

The co-primary outcomes were (1) cognitive impairment: more than 1 SD below the mean of development (toddlers) or cognition/intelligence quotient (later ages) and (2) Metabolic risk: overweight/obesity; increased waist circumference; increased fat mass or fat mass percentage; elevated plasma triglyceride concentrations; low high-density lipoprotein (HDL) concentrations; elevated low-density lipoprotein (LDL) concentrations; elevated fasting plasma glucose concentrations; insulin resistance; impaired glucose tolerance; diagnosis of type 2 diabetes; high blood pressure and impaired flow-mediated vasodilatation (Table S10 for definitions).

The secondary outcomes (Table S11 for definitions) were: (1) composite of survival free of any disability (including death, cerebral palsy, motor development delay or impairment, cognition/intelligence delay or impairment, language delay, visual impairment, hearing impairment); (2) cognition/intelligence delay or impairment; (3) cognition/intelligence scores; (4) motor delay or impairment; (5) motor scores; (6) cerebral palsy (any); (7) severity of cerebral palsy; (8) visual impairment; (9) hearing impairment; (10) school performance; (11) measures of psychological well-being; (12) metabolic outcomes: waist circumference, overweight/obese, type-2 diabetes, blood lipid concentrations (triglycerides, HDL, LDL, HDL:LDL), fasting blood glucose concentration, insulin concentration, insulin resistance, glucose tolerance, IGF-I concentration; (13) cardiovascular risk outcomes: blood pressure (systolic, diastolic, mean arterial pressure), flow-mediated vasodilatation, measures of sympathetic and parasympathetic tone, cardiac size and structure; (14) brain development: whole brain, white matter and grey matter volumes and volumes of individual brain regions, brain maturation measured using MRI (white matter tracts, measures of diffusivity, myelination, surface folding), functional brain imaging; (15) health outcomes; (16) nutrition: feeding tolerance; intake (milk, energy), appetite, breast feeding; (17) death (neonatal or later death up to the time of follow-up and cause of death); (18) quality of life; (19) general health and use of healthcare resources; (20) adverse events; (21) cost of intervention.

### 2.3. Quality Assessment

The quality of eligible trials was assessed using the methods specified in the Cochrane Handbook for Systematic Reviews of Interventions [17]: (1) random sequence generation (selection bias); (2) allocation concealment (selection bias); (3) blinding of participants, personnel and outcome assessment (performance and detection bias); (4) incomplete outcome data (attrition bias); (5) selective reporting (reporting bias); (6) other bias (checking for bias due to problems not covered by (1) to (5) above).

### 2.4. Data Synthesis and Statistical Analysis

Trialists provided de-identified data which were recoded as required, verified and checked for consistency with published data. Each trial final dataset was verified by the original trialists before individual analysis using IPD-MA prespecified variables and outcomes and the results returned to the trialists for verification. The individual trial datasets were then combined for IPD meta-analysis. There was no imputation for missing data.

We used a one-stage approach for the analysis of each outcome so that the IPD from all eligible trials were included in a single random effects model. Binary outcomes were analysed using log binomial regression models and data were reported as Relative Risk (RR) with 95% CIs and associated 2-sided *p*-values. We changed the algorithm to get the models to converge where specified. Continuous data were analysed using linear regression models and data were reported as mean differences (MD) with 95% CIs and associated 2-sided *p*-value. All models included adjustment for prespecified confounders. The analyses of IPD were adjusted for sex, gestational age and birthweight z-scores.

The overall probability of a type 1 error was maintained at 5% by splitting the *p*-value equally between the co-primary outcomes and testing each at *p* = 0.025. No further adjustment was made for multiplicity in secondary and exploratory analyses. We explored the effects of infant sex by presenting data separately for each sex as prespecified subgroups, and by testing a treatment by sex interaction term within the model. Statistical analyses were performed using SAS (v.9.4, SAS Institute, Cary, NC, USA). We validated the one-stage model using a two-stage approach in RevMan 5.3 [18].

### 2.5. Planned Subgroup and Sensitivity Analyses

Where data were available, subgroup analyses were performed to explore whether the effect of supplements differed between subgroups with sex, size of infant at birth (≤1 kg vs. >1 kg at birth), size for gestation at birth (≤10th centile vs. >10th centile), gestational age (from ≤28 completed weeks vs. from 29–32completed weeks vs. from 33–36 completed weeks), timing of supplementation (in hospital vs. after discharge vs. both), type of supplement (protein vs. carbohydrate vs. fat vs. multicomponent and their interactions), primary feed (breastmilk vs. formula vs. parenteral combined with enteral feed), trial timing (conducted before or after 2000) and duration of supplement (1 to 2 weeks vs. 3 to 6 weeks vs. more than 7 weeks) and tested for interactions. No unplanned analyses were performed.

Sensitivity analyses were performed to assess whether the results were robust to trial design by excluding trials assessed as high risk of bias. Where trials were unable to contribute to the IPD, we assessed the robustness of exclusion of these trials by combining their aggregate data (AD) with the IPD. The analyses of combined IPD and AD were adjusted for gestational age.

## 3. Results

### 3.1. Search Results

**Figure 1:**
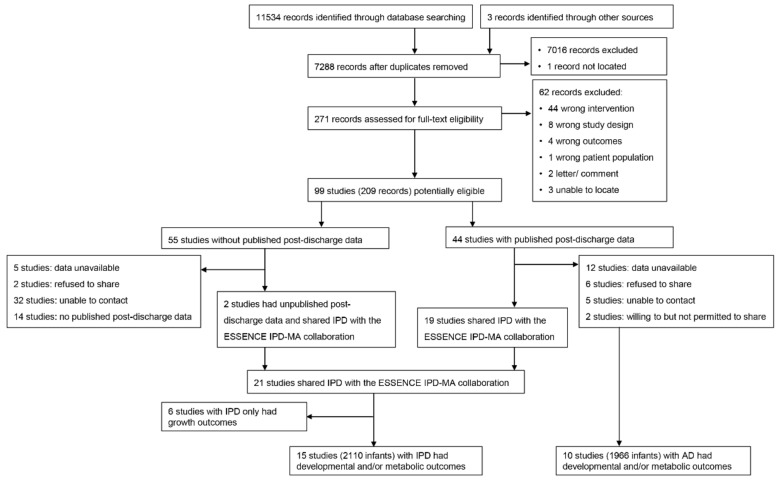
Flow diagram of included studies.

After de-duplication, 7288 records were identified (Figure 1).

After title and abstract screening, we completed full-text screening for 271 records, of which 62 did not meet our inclusion criteria. The remaining 99 potentially eligible trials (209 records) were included, among which 44 had published post-discharge data and 55 did not. We attempted to contact the authors of these 99 trials. Authors of 21 trials agreed to share IPD, for which 15 [5,19,20,21,22,23,24,25,26,27,28,29,30,31,32] had post-discharge developmental or metabolic outcome data, while 10 trials [33,34,35,36,37,38,39,40,41,42] for which IPD data were not available had published developmental or metabolic outcomes (Table 1). In total, 4106 infants were included in this analysis. Among these, 2110 infants from 15 trials were included in analysis of IPD only and 1996 infants from 10 trials were included in the analysis of combined IPD and AD.

### 3.2. Quality of the Included Studies

Methodological quality of included studies varied (Table S1). The infants in one study were selected based on their birth dates, which presented a high risk of selection bias [21]. Due to lack of blinding, 40% of the included studies were at high risk of performance bias, and two [19,24] were at high risk of attrition bias due to imbalances in baseline characteristics in those who were followed up.

### 3.3. Co-Primary Outcome: Cognitive Impairment

**Figure 2:**
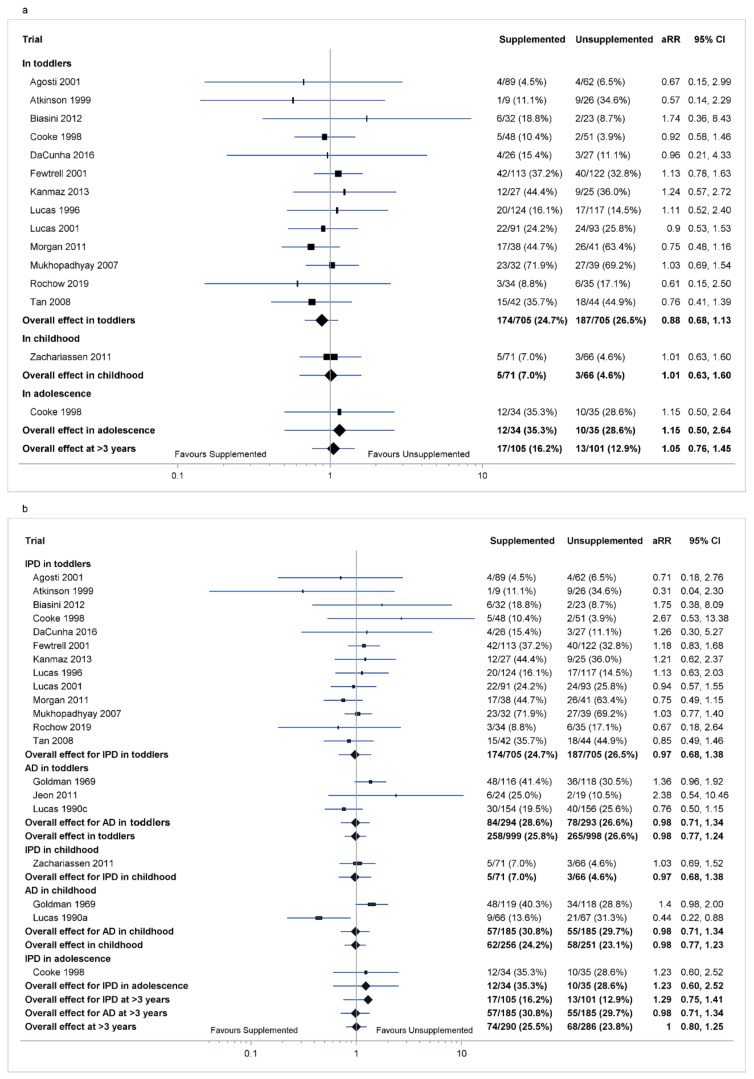
Effect of macronutrient supplementation on cognitive impairment. (**a**) IPD analysis; (**b**) combined IPD and AD analysis. The box size of the point estimate is proportional to inverse variance. Heterogeneity of IPD analysis in toddlers *p* = 0.71, tau^2^ = 0.02; at >3 years *p* = 0.63, tau^2^ = 0.03. Heterogeneity of combined IPD and AD analysis in toddlers tau^2^ = 0.02, in childhood tau^2^ = 0.01, at >3 years tau^2^ = 0.01. Numbers in bold are overall effects.

In toddlers, there was no effect of supplementation on cognitive impairment in the analysis of IPD (aRR 0.88 [95% CI 0.68, 1.13], *p* = 0.31, Tau^2^ = 0.02; 13 trials, 1410 toddlers; Figure 2a) or in the combined IPD and AD (aRR 0.98 [0.77, 1.24], *p* = 0.83, Tau^2^ = 0.02; 16 trials, 1997 toddlers; Figure 2b).

In childhood, there was no effect of supplementation on cognitive impairment in the analysis of IPD (aRR 1.01 [0.63, 1.60], *p* = 0.98; one trial, 137 children; Figure 2a) or of combined IPD and AD (aRR 0.98 [0.77, 1.23], *p* = 0.89, Tau^2^ = 0.01; three trials, 507 children; Figure 2b).

In adolescence, there was no effect of supplementation on cognitive impairment in the analysis of IPD adjusting for sex, gestational age and birthweight z-scores (aRR 1.15 [0.50, 2.64], *p* = 0.73; one trial, 69 children; Figure 2a) or adjusting for gestational age (aRR 1.23 [0.60, 2.52], *p* = 0.56; one trial, 69 children; Figure 2b). There was no AD for cognitive impairment in adolescence.

At >3 years, there was no effect of supplementation on cognitive impairment in the analysis of IPD (aRR 1.05 [0.76, 1.45], *p* = 0.77, Tau^2^ = 0.03; two trials, 206 children; Figure 2a) or of combined IPD and AD (aRR 1.00 [0.80, 1.25], *p* = 0.97, Tau^2^ = 0.01; four trials, 576 children; Figure 2b).

### 3.4. Co-Primary Outcome: Any Metabolic Risk

**Figure 3:**
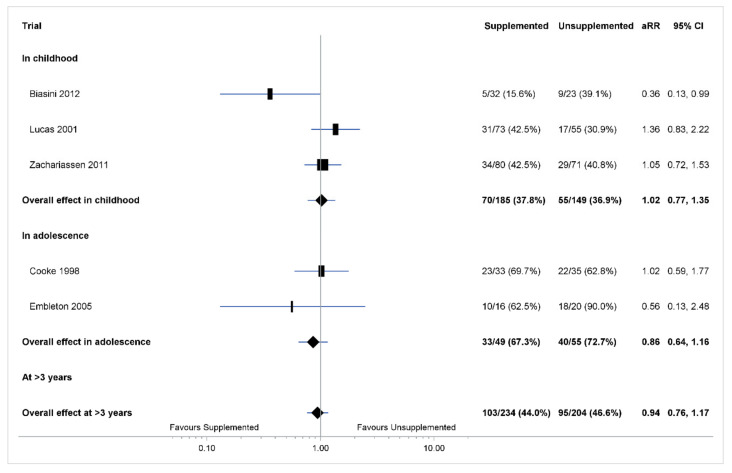
IPD analysis of any metabolic risk. The box size of point estimate is proportional to inverse variance within each age group. Heterogeneity in childhood *p* = 0.23, tau^2^ = 0.02; in adolescence = 0.15, tau^2^ = 0.02; at >3 years = 0.07 tau^2^ = 0.01. Numbers in bold are overall effects.

Supplemented and unsupplemented groups did not differ for any metabolic risk in childhood (aRR 1.02 [0.77, 1.35], *p* = 0.90, Tau^2^ = 0.02; three trials, 334 children; Figure 3), in adolescence (aRR 0.86 [0.64, 1.16], *p* = 0.31, Tau^2^ = 0.02; two trials, 104 children; Figure 3), or at >3 years (aRR 0.94 [0.76, 1.17], *p* = 0.59, Tau^2^ = 0.01; five trials, 438 children; Figure 3).

### 3.5. Secondary Developmental Outcomes

Supplemented and unsupplemented groups had similar cognitive scores in the analysis of IPD and of combined IPD and AD in toddlers, childhood, adolescence or at >3 years (Figure S1).

Toddlers in the supplemented group had a lower risk of motor impairment than those in the unsupplemented group in the analysis of IPD and of combined IPD and AD (Figure S2). Toddlers in the supplemented group also had higher motor scores in the analysis of IPD but not in the analysis of combined IPD and AD (Figure S3). There were no IPD available for motor scores in childhood, but in the analysis of AD, there was no significant effect of supplementation on motor scores.

### 3.6. Secondary Metabolic Outcomes

There were no differences between supplemented and unsupplemented groups in the analysis of IPD or of combined IPD and AD in childhood, adolescence, or at >3 years for systolic blood pressure (Figure S4), diastolic blood pressure (Figure S5) or mean arterial pressure (Figure S6).

Children in the supplemented group had lower triglyceride concentrations than children in the unsupplemented group in the analysis of IPD but not in the analysis of combined IPD and AD in childhood, and not in the analysis of IPD or of combined IPD and AD in adolescence or at >3 years (Figure S7).

There were no differences in cholesterol (Figures S8–S10), fasting blood glucose (Figure S11) or insulin concentrations (Figure S12) or BMI (Figure S13) between supplemented and unsupplemented groups in childhood, adolescence or at >3 years in the analysis of IPD or of combined IPD and AD, nor for BMI z-scores in the analysis of IPD (Figure S14). There were also no differences in IGF-1 concentrations in the analysis of IPD in adolescence (Figure S15).

There were no data for any outcomes >18 years.

**Figure 4:**
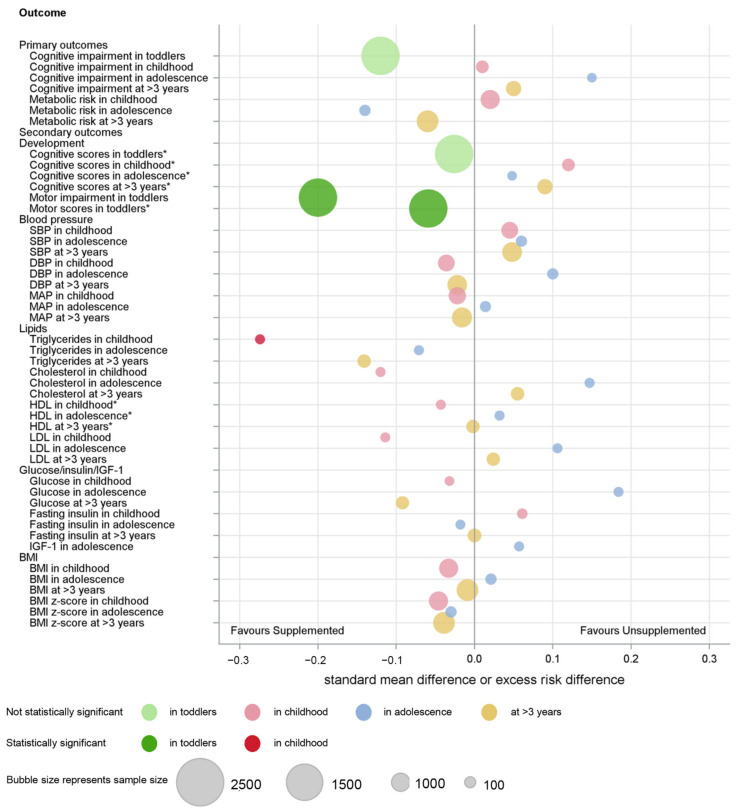
Summary of IPD analysis of macronutrient supplements on developmental and metabolic outcomes. * Direction of difference reversed.

**Figure 5:**
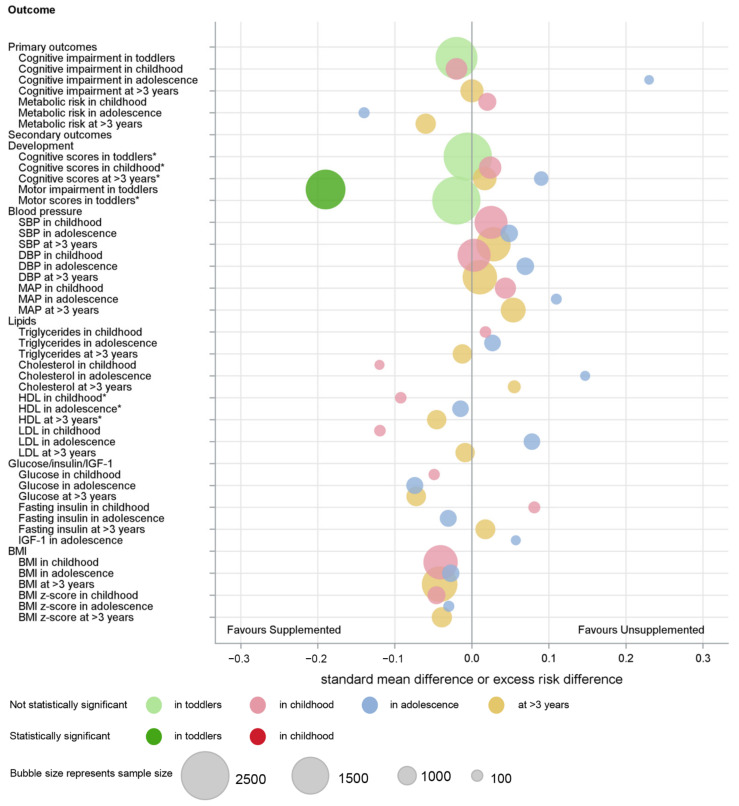
Summary of combined IPD and AD analysis of macronutrient supplements on developmental and metabolic outcomes. * Direction of difference reversed.

An overview of the main findings of the meta-analyses is provided graphically (Figure 4 and Figure 5).

### 3.7. Subgroup Analyses

#### 3.7.1. Sex of Infant: Primary Outcomes

**Figure 6:**
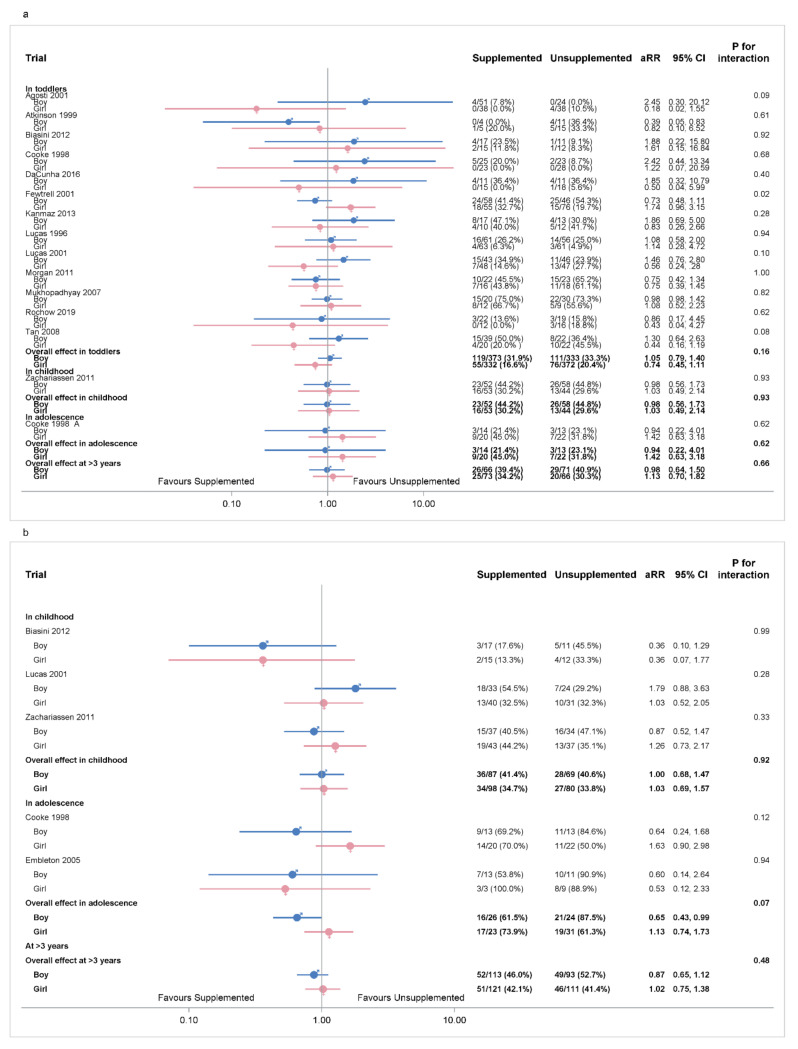
IPD analysis of primary outcomes separated by sex. (**a**) Cognitive impairment; (**b**) metabolic risks. Heterogeneity: a. cognitive impairment, boys in toddlerhood *p* = 0.90, tau^2^ = 0.02; at >3 years *p* = 0.98, tau^2^ = 0.04; girls in toddlerhood *p* = 0.89, tau^2^ = 0.04; at >3 years = 0.96, tau^2^ = 0.06. Heterogeneity: b. metabolic risks, boys in childhood *p* = 0·16, tau^2^ = 0.06; in adolescence *p* = 0.83, tau^2^ = 0.03; at >3 years *p* = 0.0009, tau^2^ = 0.02; girls in childhood *p* = 0·66, tau^2^ = 0.06; in adolescence *p* = 0.01, tau^2^ = 0.04; at >3 years *p* < 0.0001, tau^2^ = 0.02. Numbers in bold are overall effects.

Cognitive impairment was not different between supplemented and unsupplemented groups in boys or girls, and no significant sex interactions were identified (Figure 6a). In childhood and at >3 years the supplemented and unsupplemented groups were similar for metabolic risk in boys and girls, and there were no significant sex interactions (Figure 6b). In adolescence, supplementation reduced metabolic risk in boys (aRR 0.65 [0.43, 0.99], *p* = 0.04, Tau^2^ = 0.00; two trials, 50 boys), but not in girls (aRR 1.13 [0.74, 1.73], *p* = 0.58, Tau^2^ = 1.72; two trials, 54 girls), but the sex interaction was not significant (*p* = 0.07).

#### 3.7.2. Sex of Infant: Secondary Outcomes

Cognitive scores were similar between supplemented and unsupplemented groups in boys and girls, and there were no sex interactions (Figure S16a).

In toddlers, supplementation reduced the risk of motor impairment in girls but not boys (*p* = 0.03 for sex interaction) (Figure S16b). Similarly, motor scores were higher in the supplemented group in girls but not boys, but the sex interaction was not significant (Figure S16c).

There were no differences between supplemented and unsuppplemented groups in boys and girls, and no significant sex interactions for systolic, diastolic and mean blood pressures (Figure S17), HDL and LDL concentrations (Figure S18), blood glucose and fasting insulin (Figure S19), IGF-I concentrations (Figure S19c), BMI or BMI z-scores (Figure S20).

Supplemented boys, but not girls, had lower triglyceride concentrations at >3 years, and higher cholesterol concentrations in adolescence, but the sex interaction terms were not significant, and there were no differences in boys or girls at other ages between the groups (Figure S18).

#### 3.7.3. Size for Gestation of the Infant

The effects of supplements on cognitive impairment or any metabolic risk were not different in SGA and appropriate gestational age (AGA) subgroups, and there were no significant interactions (Table S2). However, supplemented children born SGA but not those born AGA had less motor impairment in toddlerhood but lower cognitive scores in childhood. There were significant interactions between the effects of supplements on some metabolic outcomes and size for gestation, with supplemented children born SGA but not those born AGA having lower fasting triglyceride but higher cholesterol concentrations at >3 years, and higher fasting insulin concentrations in adolescence than unsupplemented groups.

#### 3.7.4. Size of Infant at Birth

The effects of supplements on cognitive impairment or any metabolic risk was not different in babies with birthweight ≤1 kg and with birthweight >1 kg (Table S3). In childhood, supplemented children with birthweight >1 kg had lower triglyceride concentrations than unsupplemented children, but there were no effects of supplementation for children with birthweight ≤1 kg, and no significant interactions.

#### 3.7.5. Gestational Age of Infant at Birth

There were no differences between supplemented and unsupplemented groups in different gestational age subgroups for cognitive impairment or any metabolic risk (Table S4). Supplemented children born very preterm (28-32 weeks), but not those born extremely (<28 weeks) or moderate-late (32-37 weeks) preterm, had lower BMI and BMI z-scores as toddlers and in childhood than unsupplemented children. Supplemented children had better motor scores in toddlerhood, lower fasting HDL concentrations and higher fasting blood glucose concentrations in adolescence and at >3 years than unsupplemented children only in the subgroup born moderate–late preterm, and lower triglyceride concentrations in childhood and lower fasting blood glucose concentrations in adolescence only in the subgroup born very preterm, but there were no significant interactions.

#### 3.7.6. Timing of Supplement

There were no differences between supplemented and unsupplemented groups for cognitive impairment in the subgroups who received supplements in hospital or post-discharge (Table S5). Children who received supplements in hospital, but not those who received supplements post-discharge, had lower incidence of any metabolic risk, lower BMI and BMI z-score in adolescence and at >3 years, and lower triglyceride concentrations at >3 years (all interaction terms *p* < 0.05).

#### 3.7.7. Type of Supplement

There were no differences between supplemented and unsupplemented groups for cognitive impairment in the subgroups who received protein and who received multicomponent fortification (Table S6). Supplemented children receiving additional protein, but not those who received multicomponent fortification, had lower incidence of any metabolic risk, lower BMI, BMI z-score in adolescence and at >3 years, and lower triglyceride concentrations at >3 years (all interaction terms *p* < 0.05).

#### 3.7.8. Primary Feed

There were no differences between supplemented and unsupplemented groups for cognitive impairment and metabolic risk in the subgroups of children whose primary feed was breastmilk, formula, or a combination of parenteral and enteral feeds (Table S7). Only supplemented children who had received breast milk as primary milk feed had higher motor scores as toddlers, and lower triglyceride concentrations at >3 years than unsupplemented children, but none of the interaction terms were significant. These effects were not due to differences between the primary feed groups in baseline macronutrient intakes or quantity of the supplements (Text S2).

#### 3.7.9. Different Trial Timing

There were no differences between supplemented and unsupplemented groups for cognitive impairment in the subgroups of trials conducted before or after 2000 (Table S8). In trials conducted after 2000, but not those conducted before, supplemented children had lower metabolic risk in adolescence, lower BMI and BMI z-scores in adolescence and at >3 years, and lower fasting blood glucose and fasting insulin concentrations in adolescence than unsupplemented children. The interaction terms were significant only for metabolic risk in adolescence, BMI and BMI z-scores in adolescence and at >3 years. These effects were not due to changes over time in baseline macronutrient intake or quantity of the supplements (Text S2).

There were insufficient data to allow subgroup analyses of duration of supplement (1 to 2 weeks vs. 3 to 6 weeks vs. more than 7 weeks).

## 4. Discussion

We found that early macronutrient supplementation of preterm and SGA infants did not alter cognitive function nor increase metabolic risk in toddlers and older children, although the data are limited for older ages. This contrasts with previous observational studies [43,44] suggesting that greater macronutrient intake was associated with better cognitive development in preterm infants.

Early macronutrient supplements improved motor function in toddlers, especially for girls. Children born preterm are at three to four times greater risk of motor impairment than the general population [43]. Even in the absence of cerebral palsy, developmental coordination disorder (DCD) is a common motor impairment, particularly in children born preterm [44,45,46] with deficits in coordination, balance, gross and fine motor control that can interfere with academic performance and activities of daily living [47]. Thus, the benefit of macronutrient supplements on motor function, although small in magnitude in toddlers, might be clinically important if it persisted into later childhood, but there are insufficient data to assess this.

Macronutrient supplementation of preterm and SGA infants leads to greater weight and length in toddlers [13]. However, faster growth during critical periods in early life has been associated with detrimental effects on long-term metabolic outcomes [48,49,50,51,52]. Therefore, we hypothesised that macronutrient supplementation may increase risk of later metabolic disease. This was not supported by our analysis of IPD, which showed no adverse effect on later metabolic outcomes. Indeed, supplementation was associated with lower triglyceride concentration in childhood in the analysis of IPD, but not in adolescence or at >3 years or in the combined analysis of IPD and AD. Although these findings are limited by the small number of randomised trials and few data from children at older ages, we found no evidence that early macronutrient supplementation has adverse effects on later metabolic outcomes.

A number of epidemiological studies have investigated the sex differences in long-term outcomes following early life exposures. Males seem to be more sensitive to insults in utero than females, and females have better outcomes in the perinatal period, particularly after preterm birth [53,54]. We did not find sex-specific effects on cognitive outcomes, but did find that supplements reduced motor impairment and improved motor scores for toddler girls but not boys. This is not consistent with findings of a systematic review of published randomised trials [12] that supplements had no sex-specific effect on motor function, but improved cognitive scores for toddler boys and not for girls. That systematic review was limited to two trials with 400 toddlers, whereas we included 13 trials with more than 1400 toddlers in this IPD-MA. The effects of supplements on later metabolic risk did not show clear sex-specificity.

To further explore the effect of early macronutrient supplements, we conducted several other prespecified subgroup analyses. In the subgroup analysis of SGA versus AGA babies, supplemented children born SGA had lower cognitive scores in childhood, but not in toddlerhood, adolescence or at >3 years. However, the evidence is insufficient to draw firm conclusions since only one trial of 20 children born SGA reported cognitive scores in childhood. Possible adverse effects of nutritional supplements on cognitive scores in toddlers born SGA were reported in two previous randomised trials, one included in the analysis of IPD [25] and one included in the analysis of combined IPD and AD [36]; thus, further investigation is warranted.

We also found supplementation decreased triglyceride but increased cholesterol concentrations at >3 years and increased fasting insulin concentrations in adolescence for children born SGA, but not for children born AGA. One systematic review of observational studies [51] of term-born SGA infants reported that rapid postnatal weight gain seemed to adversely affect adiposity and related markers of metabolic health at later ages. Many animal studies have also reported adverse metabolic outcomes in those born SGA who received a high-nutrient diet after birth [55]. However, since our analyses included only three trials with fewer than 25 children born SGA, we are unable to draw a definite conclusion about effects of macronutrient supplements for children born SGA.

In the subgroup analysis of different gestational age groups at birth, supplements improved motor scores in toddlers, decreased HDL concentrations and increased fasting blood glucose concentrations in adolescence and at >3 years in the moderate–late preterm subgroup. However, the numbers of included trials and children was smallest in this group, and the reasons for differences between gestational age groups are not clear. One observational study [56] reported that rapid weight gain in the first four months was associated with overweight or obesity in childhood for moderate–late preterm infants, but not for infants born <34 weeks.

We found that BMI, triglyceride and fasting blood glucose concentrations at >3 years were all lower in the supplemented compared to non-supplemented children when they received in-hospital nutritional supplements and protein as the type of supplement, but the effects were not seen in children who received post-discharge nutritional supplements and multicomponent supplements. A previous systematic review [13] found that toddler size increased in supplemented infants who received post-discharge nutrition, but not in those who received supplements in hospital. It is possible that increasing macronutrient intake after discharge may hasten ‘catch-up’ growth and that this ‘catch-up’ growth may influence the risk of adverse metabolic outcomes [57]. It is also possible that higher intake of protein rather than other macronutrients may have different effects on later metabolic disease. Protein intake in early life is associated with linear growth up to 36 weeks’ corrected age [58,59], so protein might promote growth of lean mass, which could minimise metabolic risk, whereas other macronutrients might promote fat accumulation and increase metabolic risk. However, the effects of the timing and composition of supplements cannot be distinguished in our analysis, as some trials studied in-hospital supplements with protein as the primary supplement, whereas other trials studied post-discharge supplementation with multicomponent supplements. We were also concerned that there might be similar overlaps between different size for gestational age at birth and timing of supplements, i.e., infants born SGA may have received in-hospital nutrition, whereas AGA infants may have received post-discharge nutrition. Although we did not find any significant interactions between timing of supplements and size for gestational age, data on timing of supplements were only available at the trial rather than at the individual participant level, so we are unable to exclude possible interactions between the supplement composition, timing, and size for gestation.

Previous studies have consistently reported an advantage in developmental scores in infants who were fed breast milk [60,61,62]. We found better motor scores in toddlers and lower triglyceride concentration at >3 years only in supplemented children who had breast milk as their primary feed, but not if the primary feed was formula or if supplements were provided as both parenteral and enteral feeds, although the interaction terms were not significant. Breastmilk generally has lower energy and protein content than formula, so infants whose primary feed was breastmilk may have received less total nutrition or less additional nutrition in the supplemented group. However, we found that unsupplemented infants had planned macronutrient intakes that were similar regardless of their primary feed. Moreover, supplemented infants receiving breastmilk as their primary feed had higher protein, carbohydrate and energy intakes than unsupplemented infants, whereas supplemented infants receiving formula as primary feed had a smaller increase in protein and much smaller increases in carbohydrate and energy. Protein may be more important than other macronutrients for early development [59,63], and this might explain the improved motor scores of supplemented children who received breast milk as primary feed.

In the subgroup analyses of different trial epochs, we found that supplemented infants had lower BMI and BMI z-scores in adolescence or at >3 years only in the studies conducted after 2000. This could not be attributed to unsupplemented infants in later studies receiving higher macronutrient intakes, leading to smaller effects of additional supplements compared to earlier studies. However, our estimations may not accurately reflect the actual intake of infants because they were based on trial-level data and there are wide variations in estimated macronutrient composition of preterm human milk, and in the compositions of formula and fortifier over time [64,65].

Previously, three systematic reviews have compared the effects of fortified/unfortified breastmilk started in hospital and after discharge, and supplemented versus unsupplemented formula after discharge [66,67,68] and one [12] evaluated the effect of macronutrient supplements in preterm and SGA infants. However, systematic reviews of published studies are limited by which outcomes are published, within-trial variation in gestational age, size of the infants at birth, nature of the interventions and quality of evidence. A key strength of our study is that IPD-MA is the gold standard for systematic reviews [69], allowing inclusion of a large sample size and exploration of different subgroups, including potential sex-specific effects. IPD also allowed us to use consistent outcome definitions, for example of motor impairment and metabolic risk, and to adjust for potential confounding by sex, gestational age and size at birth [16]. Further, the quality of the included trials was assessed based on both the published paper and on the details provided by the trialists, with the result that the overall risk of bias was low across most included studies.

Our IPD-MA has some limitations. Although we identified 22 eligible trials with published post-discharge outcomes, only 12 trials and 52% of the originally randomised infants were included, potentially leading to inclusion bias. To address this, we conducted sensitivity analyses combining both IPD and AD. Most gave results consistent with the analysis of IPD only, except for the motor scores in toddlers and triglycerides in childhood, where differences between supplemented and unsupplemented groups were no longer statistically significant in the combined analysis. Furthermore, a post hoc calculation showed that the analysis of IPD alone (1410 children) had sufficient power to detect a 6.3% difference in cognitive impairment or three-point difference in cognitive scores between groups with 80% power and alpha of 0.05, 5% type I error. Therefore, it is unlikely that clinically important effects of supplementation were missed. Nevertheless, not all trials collected or were able to provide data for the prespecified subgroups, which may have limited the power to be able to detect subgroup differences for some important outcomes. Furthermore, a large number of subgroups, multiple outcomes and multiple timepoints were analysed, which increases the risk of type 1 error, [70], and subgroup results should be interpreted cautiously when generating a hypothesis. In addition, the included studies were of different types of macronutrient supplements given at different times for different durations. Although we followed the strategies suggested by the Cochrane handbook to address heterogeneity [17], significant unexplained heterogeneity remained for some outcomes.

This ESSENCE IPD-MA shows that early macronutrient supplements may not alter cognitive function nor increase later metabolic risks for infants born preterm or SGA. However, early macronutrient supplements may improve motor function in toddlers born small, particularly for girls, although the long-term effects on motor function are unclear.

## Figures and Tables

**Table 1:** Included trials and their characteristics.

Author/Year	Country	Participants	Participants, *n*	Intervention	Control	Duration	Outcomes
Studies with IPD Available							
Agosti 2003 [19]	Italy	Inclusion criteria: preterm BW < 1500 g and previously fed with a preterm formula. Exclusion criteria: malformations intraventricular haemorrhage, periventricular leukomalacia, chronic lung disease, necrotising enterocolitis grade >1, total parenteral nutrition >2 weeks, sepsis, retinopathy of prematurity grade >1.	Intervention: 89 Control: 67	Preterm formula (protein 2.4 g/100 mL, energy 80 kcal/100 mL)	Standard term formula (protein 1.7 g/100 mL, energy 70 kcal/100 mL)	Started from 40 weeks PMA, stopped at 55 weeks PMA.	GMDS at 6, 9, 12 and 18 months’ CA
Atkinson 1999 [20]	Canada	Inclusion criteria: BW < 2500 g; GA < 42 weeks; birthweight <5th percentile and fed only formula at entry into the study.	Intervention: 22 Control: 28	Ross Discharge formula (protein 1.8 g/100 mL, energy 74 kcal/100 mL)	Similac with Iron formula (energy 68 kcal/100 mL)	Started from discharge, stopped at 1 year CA	Bayley II at 6 and 12 months’ CA.
Biasini 2012 [21]	Italy	Inclusion criteria: BW 580–1250 g and GA < 32 weeks.	Intervention: 34 Control:27	Protein supplemented (protein 4.8 g/kg/day, energy 141 kcal/day)	Control (protein 3.5 g/kg/day, energy 135 kcal/day)	Started from the first day of full enteral feeding, stopped at discharge.	GMDS at 3, 6, 9, 12, 15, 18 and 24 months’ CA.
Cooke 1998 [22]	UK	Inclusion criteria: GA ≤ 34 weeks and BW ≤ 1750 g, and growing normally at the time of hospital discharge, i.e., ≥25 g/day. Exclusion criteria: systemic disease or require medication.	Intervention: 56 Control: 57	Preterm formula (protein 2.2 g/100 mL, fat 4.4 g/100 mL, carbohydrate 8.5 g/100 mL, energy 80 kcal/100 mL)	Term formula (protein 1.4 g/100 mL, fat 3.6 g/100 mL, carbohydrate 7.5 g/100 mL, energy 66 kcal/100 mL)	Started from discharge, stopped at 6 months’ CA.	Bayley II at 18 months’ CA; WISC at 10 years’ CA; blood pressure, triglyceride, cholesterol, HDL, LDL, fasting blood glucose concentration, fasting insulin concentration IGF-I at 13 years’ CA.
da Cunha 2016 [23]	Brazil	Inclusion criteria: GA < 37 weeks and BW < 1500 g, and discharged exclusively breastfeeding. Exclusion criteria: major malformations; hydrocephalus; chromosomal abnormalities; fetal hydrops; congenital infections; maternal use of illicit drugs, tobacco, alcohol and continuous use of corticosteroids; twin pregnancy; necrotising enterocolitis sequelae; cerebral palsy.	Intervention: 26 Control: 27	Breast milk supplementation (daily increase of 0.56 g of protein, 1.04 g of total fat and 2.12 g of carbohydrates)	Breast milk without supplementation	Started 7–10 days after discharge, stopped at four to six months.	Bayley III at 12 months’ CA.
Embleton 2005 [24]	UK	Inclusion criteria: GA ≤ 34 weeks and BW ≤ 1750 g, tolerating enteral intake ≥150 mL/kg/day for ≥48 h and current weight ≥1000 g.	Intervention: 25 Control: 26	Formula A (protein 2.6 g/100 mL, fat 4.3 g/100 mL, carbohydrate 7.9 g/100 mL, energy 80 kcal/100 mL)	Formula C (protein 2.2 g/100 mL, fat 4.5 g/100 mL, carbohydrate 7.9 g/100 mL, energy 80 kcal/100 mL)	Started when full enteral feeding 150 mL/kg/day, stopped at 12 week’ CA.	Blood pressure, triglyceride, cholesterol, HDL, LDL, fasting blood glucose concentration, fasting insulin concentration, IGF-I at 10 years’ CA.
Fewtrell 2001 [25]	UK	Inclusion criteria: GA ≥ 37 weeks and BW below the 10th centile for gestation and sex according to UK growth charts.	Intervention: 152 Control: 147	Enriched formula (protein 1.85 g/100 mL, fat 3.96 g/100 mL, carbohydrate 7.24 g/100 mL, energy 72 kcal/100 mL)	Term formula (protein 1.45 g/100 mL, fat 3.85 g/100 mL, carbohydrate 6.96 g/100 mL, energy 68 kcal/100 mL)	Started within the first week, stopped at 9 months’ CA.	Bayley II at 18 months’ corrected age.
Kanmaz 2013 [26]	Turkey	Inclusion criteria: GA ≤ 32 weeks and BW ≤ 1500 g; fed with human milk. Exclusion criteria: major congenital anomalies, chronic illnesses, respiratory support requirements, or sepsis and those who were receiving mixed feeding.	Intervention: 29 Control: 26	Aggressive fortification: 1.2 g of human milk fortifier added to each 20 mL human milk (protein: 3.6 g/kg/day)	Standard fortification: 1.2 g of human milk fortifier added to each 30 mL human milk (protein: 3.0 g/kg/day)	Started when infants reached 90–100 mL/kg enteral feeding, stopped at discharge.	Bayley II at 2 years’ corrected age.
Lucas 1996 [5]	UK	Inclusion criteria: BW < 1850 g, GA < 37 weeks, and survived to be assigned to a study group between 48 and 72 h of age. Exclusion criteria: major congenital anomalies.	Intervention: 137 Control: 138	Fortified human breast milk; fortifier containing protein 0.7 g/100 mL, fat 0.05 g/100 mL, carbohydrate 2.73 g/100 mL, energy 14 kcal/100 mL	Human breast milk	Started within 48 h, stopped at discharge or when the infants reached 2000 g.	Bayley II at 18 months’ CA.
Lucas 2001 [27]	UK	Inclusion criteria: GA < 37 weeks and BW < 1750 g. Exclusion criteria: congenital malformations or conditions known to affect growth or development.	Intervention: 113 Control: 116	Post-discharge formula (protein 1.85 g/100 mL, fat 3.96 g/100 mL, carbohydrate 7.24 g/100 mL, energy 72 kcal/100 mL)	Term formula (protein 1.45 g/100 mL, fat 3.82 g/100 mL, carbohydrate 6.96 g/100 mL, energy 68 kcal/100 mL)	Started one week before discharge, stopped at 9 months post-term.	Bayley II at 18 months’ CA. Blood pressure at 5 years, body composition at 5 years.
Morgan 2014 [28]	UK	Inclusion criteria: GA 24–28 weeks and BW < 1200 g. Exclusion criteria: unlikely to survive the first week after birth; diagnosed with major congenital or chromosomal abnormalities known to affect gastrointestinal function or head growth, including definite parenchymal lesions on cranial ultrasound scan in first 48 h.	Intervention: 74 Control: 76	Higher macronutrient content (parenteral intake with protein 2.8 g/kg/day, fat 2.8 g/kg/day, carbohydrate 13.5 g/kg/day, energy 85 kcal/kg/day)	Standard macronutrient content (parenteral intake with protein 3.8 g/kg/day, fat 3.8 g/kg/day, carbohydrate 15.6 g/kg/day, energy 103 kcal/kg/day)	Started within 120 h of birth, stopped at 28 days.	Bayley III at 2 to 3.5 years of CA.
Mukhopadhyay 2007 [29]	India	Inclusion criteria: GA ≤ 34 weeks and BW ≤ 1500 g, reached feed volume of 150 mL/kg/day, feed constituted at least 80% breast milk. Exclusion criteria: major congenital malformation, gastrointestinal abnormalities.	Intervention: 84 Control: 82	Fortified human milk: (fortifier contained protein 0.4 g/100 mL; fat 0.2 g/100 mL; carbohydrate 2.4 g/100 mL; energy 13 kcal/100 mL)	Exclusive human milk.	Started when feed volume reached 150 mL/kg/day, stopped when reached 2 kg or full breastfeeds.	Bayley II at 12 months’ CA.
Rochow 2019 [30]	Canada	Inclusion criteria: GA < 30 weeks, and length of stay > 21 days and receiving fortified BM. Exclusion criteria: gastrointestinal malformation, major congenital anomalies, necrotising enterocolitis abdominal surgery, and gram-negative sepsis.	Intervention: 52 Control: 51	Target fortified human milk:(protein 3.0 g/100 mL, fat 4.4 g/100 mL, carbohydrates 8.5 g/100 mL)	Standard fortified human milk	Started when enteral intake was ≥100 mL/kg/day, stopped at 36 weeks’ PMA.	Bayley III at 18 months’ CA.
Tan 2008 [31]	UK	Inclusion criteria: GA < 29 weeks. Exclusion criteria: triplets and higher multiplicity, admitted after 7 days of age, major congenital abnormalities	Intervention: 68 Control: 74	Parenteral protein 4 g/kg/day, fat 4 g/kg/day, carbohydrate 16.3 g/kg/day, energy 117 kcal/kg/day; enteral breast milk or formula with target protein 4 g/kg/day, energy 133–150 kcal/kg/day	Parenteral protein 3 g/kg/day, fat 3 g/kg/day, carbohydrate 13.5 g/kg/day, energy 93 kcal/kg/day; enteral breast milk or formula with target protein 3.3 g/kg/day, energy 133 kcal/kg/day	Started when infants received parenteral and enteral nutrition from the first week, stopped at 34 weeks’ PMA.	Bayley II at 3 and 9 months’ CA.
Zachariassen 2011 [32]	Denmark	Inclusion criteria: GA ≤ 32 weeks, breastfeeding. Exclusion criteria: severe diseases or circumstances influencing eating and feeding ability at discharge.	Intervention: 105 Control: 102	Fortified mother’s milk (protein 1.375 g/day, energy 17.5 kcal/day)	Unfortified mother’s milk	Started from shortly before discharge, stopped at 4 months’ CA.	WISC at 6 years’ CA; Blood pressure, triglyceride, HDL, LDL, fasting blood glucose concentration, fasting insulin concentration, at 6 years’ CA.
**Studies with AD available**							
Amesz 2010 [33]	Netherlands	Inclusion criteria: GA ≤ 32 weeks or BW ≤ 1500 g. Exclusion criteria: congenital malformations or conditions known to affect growth or body composition (e.g., severe bronchopulmonary dysplasia, an inborn error of metabolism, cardiac or renal disease, necrotising enterocolitis with substantial gut loss, grade IV intraventricular haemorrhage).	Intervention: 52 Control:50	Post-discharge formula (protein 1.7 g/100 mL, fat 3.5 g/100 mL, carbohydrate 7.0 g/100 mL, energy 67 kcal/100 mL)	Term formula (protein 1.47 g/100 mL, fat 3.5 g/100 mL, carbohydrate 7.2 g/100 mL, energy 70 kcal/100 mL)	Started from term, stopped at 6 months’ CA.	Blood pressure, triglyceride, HDL, LDL, fasting blood glucose concentration, insulin sensitivity, insulin resistance (HOMA-IR), fasting leptin at 8 years’ CA.
Bellagamba 2016 [34]	Italy	Inclusion criteria: preterm BW 500–1249 g.	Intervention: 82 Control: 82	High protein (protein supplementation started at 1.5 g/kg/day and increased by 0.5 g/kg/day to a maximum of 3.5 g/kg/day on the fifth day after birth)	Standard protein (protein supplementation started at 1.5 g/kg/day and increased by 0.5 g/kg/day to a maximum of 2.5 g/kg/day on the third day after birth)	Started from birth, stopped at discharge.	Bayley III at 2 years’ corrected age.
Dogra 2017 [35]	India	Inclusion criteria: GA < 32 weeks. Exclusion criteria: lethal congenital malformations.	Intervention: 59 Control: 56	Fortified breast milk with higher protein (fortifier containing protein 1.0 g/100 mL, fat 0.01 g/100 mL, carbohydrate 3.6 g/100 mL, energy 17.2 kcal/100 mL)	Fortified breast milk with standard protein (fortifier containing protein 0.4 g/100 mL, fat 0.2 g/100 mL, carbohydrate 2.4 g/100 mL, energy 13 kcal/100 mL)	Started when infants reached a feed volume of 100 mL/kg/day, stopped at discharge or full breast-feeds, whichever was earlier.	DASII at 12 to 18 months’ CA
Goldman 1969 [36]	USA	Inclusion criteria: BW < 2000 g. Exclusion criteria: major congenital malformations, intestinal obstruction, Rhesus disease, > 3 days old on admission, or died during the first few days generally received no milk feedings.	Intervention: 152 Control: 152	Enriched formula (protein 4.0 g/100 mL, fat 3.9 g/100 mL, carbohydrate 7.6 g/10 mL, 80 kcal/100 mL)	Standard formula (protein 2.0 g/100 mL, fat 3.9 g/100 mL, carbohydrate 9.6 g/100 mL, energy 80 kcal/100 mL)	Started from 24 to 72 h, stopped when the infants reached 2200 g (at discharge).	Cognitive impairment (Stanford–Binet scores) at 3 years’ CA.
Jeon 2011 [37]	Korea	Inclusion criteria: GA < 33 weeks and BW < 1500 g, formula as the primary food source. Exclusion criteria: chromosomal disorders or serious congenital malformations at discharge that would affect growth and development.	Intervention: 35 Control: 34	Preterm formula (protein 2.3 g/100 mL, fat 4.1 g/100 mL, carbohydrate 8.5 g/100 mL, energy 80 kcal/100 mL)	Term formula (protein 1.6 g/100 mL, fat 3.5 g/100 mL, carbohydrate 7.2 g/100 mL, energy 67 kcal/100 mL)	Started at term, stopped at 6 months’ corrected age.	Bayley II at 18 months’ CA.
Lucas 1989 [38]	UK	Inclusion criteria: GA < 37 weeks and BW < 1850 g. Exclusion criteria: major congenital abnormality known to impair growth or development, or died before randomisation within the first 48 h.	(1) Lucas 1989a: Intervention: 76 Control: 83 (2) Lucas 1989b: Intervention: 173 Control: 170 (3) Lucas 1989c: combined Lucas 1989a and Lucas 1989b: Intervention: 249 Control: 253	(1) Lucas 1989a Preterm formula as sole diet (protein 2.0 g/100 mL, fat 4.9 g/100 mL, carbohydrate 7.0 g/100 mL, energy 80 kcal/100 mL) (2) Lucas 1989b Preterm formula as supplement (3) Lucas 1989c: combined Lucas 1989a and Lucas 1989b	(1) Lucas 1989a: Banked breast milk as sole diet (protein 1.1 g/100 mL, fat 1.7 g/100 mL, carbohydrate 7.1 g/100 mL, energy 46 kcal/100 mL); (2) Lucas 1989b: banked breast milk as supplement; (3) Lucas 1989c: combined Lucas 1989a and Lucas 1989b	Started within 48 h, stopped at discharge or when the infants reached 2000 g.	Bayley II at 9, 18 months’ CA; Blood pressure at 7.5 to 8 years’ and 13 to 16 years’ CA; Triglyceride, HDL, LDL, fasting blood glucose concentration, fasting insulin concentration, insulin resistance (fasting 32–33 split proinsulin) at 13 to 16 years’ CA.
Lucas 1990 [39]	UK	Inclusion criteria: BW < 1850 g and GA < 37 weeks; Exclusion criteria: major congenital abnormality known to impair growth or development or died before randomisation within the first 48 h.	(1) Lucas 1990a:Intervention: 81 Control: 79 (2) Lucas 1990b: Intervention: 132 Control: 132 (3) Lucas 1990c: combined Lucas 1990a and Lucas 1990b: Intervention: 213 Control: 211	(1) Lucas 1990a: Preterm formula as sole diet (protein 2.0 g/100 mL, fat 4.9 g/100 mL, carbohydrate 7.0 g/100 mL, energy 80 kcal/100 mL) (2) Lucas 1990b Preterm formula as supplement (3) Lucas 1990c: combined Lucas 1990a and Lucas 1990b	(1) Lucas 1990a: term formula as sole diet (protein 1.5 g/100 mL, fat 3.8 g/100 mL, carbohydrate 7.0 g/100 mL, energy 68 kcal/100 mL) (2) Lucas 1990b: term formula as supplement (3) Lucas 1990c: combined Lucas 1990a and Lucas 1990b	Started within 48 h, stopped at discharge or when the infants reached 2000 g.	Bayley II at 9, 18 months’ CA; Wechsler Intelligence Scale for Children (WISC) at 7.5 to 8 years’ CA; Blood pressure at 7.5 to 8 years’ and 13 to 16 years’ CA; Triglyceride, HDL, LDL, fasting blood glucose concentration, fasting insulin concentration, insulin resistance (fasting 32–33 split proinsulin) at 13 to 16 years’ CA.
O’Connor 2008 [40]	Canada	Inclusion criteria: GA < 33 weeks and BW between 750 and 1800 g who received ≥80% of their total feedings as human milk 3 days before hospital discharge; Exclusion criteria: serious congenital or chromosomal anomalies that could affect growth, grade 3 or 4 periventricular or intraventricular haemorrhage, oral steroids within 14 days of randomisation, severe asphyxia and known maternal alcohol or drug abuse.	Intervention: 19 Control: 20	Human milk with a multi-nutrient fortifier (protein 2.0 g/100 mL, fat 4.2 g/100 mL, carbohydrate 8.8 g/100 mL, energy 81 kcal/100 mL)	Unfortified human milk (protein 1.3 g/100 mL, fat 3.9 g/100 mL, carbohydrate 7.2 g/100 mL, energy 68 kcal/100 mL	Started from discharge, stopped at 12 weeks after discharge.	Bayley II at 18 months’ CA.
Roggero 2012 [41]	Italy	Inclusion criteria: GA ≤ 32 weeks or BW ≤ 1500 g and being fed human milk for 20% of the total milk intake; Exclusion criteria: congenital malformations or conditions that interfere with growth or body composition.	Intervention: 110 Control: 107	Nutrient-enriched formula (protein 2.0 g/100 mL, fat 4.1 g/100 mL, carbohydrate 7.5 g/100 mL, energy 75 kcal/100 mL)	Term formula (protein 1.4 g/100 mL, fat 3.7 g/100 mL, carbohydrate 7.4 g/100 mL, energy 68 kcal/100 mL)	Started from term CA, stopped at 6 months.	GMDS at 24 months’ CA.
Svenningsen 1982 [42]	Sweden	Inclusion criteria: Very low birthweight preterm with mean BW 1385 ± 343 g and GA 30.8 ± 2.9 weeks.	Intervention: 16 Control: 14	Nutrition enriched formula (protein 2.1 g/100 mL, energy 69.5 kcal/100 mL)	Standard formula (protein 1.6 g/100 mL, energy 68.5 kcal/100 mL)	Started from the third week after birth, stopped at the seventh week after birth.	Development impairments at 6 months, 1 and 2 years of age.

## Data Availability

The deidentified participant data analysed for the ESSENCE IPD-MA project remain the property of the ESSENCE-IPD Trialist Group. Researchers should contact the original trial investigator directly for access to these data. The data dictionary, statistical analysis plan and metadata for this IPD-MA are available at doi:10.17608/k6.auckland.18729068.v1. Researchers are able to use this information and the provided contact address (researchhub@auckland.ac.nz) to request further information through the Data Access Committee of the Liggins Institute.

## Supplementary Materials

The following supporting information can be downloaded at: https://www.mdpi.com/article/10.3390/nu14030418/s1, Text S1. PRISMA-IPD Checklist of items to include when reporting a systematic review and meta-analysis of individual participant data (IPD). Text S2. Comparison of macronutrient intakes. Figure S1. Forest plot of effect of macronutrient supplementation on cognitive scores; Figure S2. Forest plot of effect of macronutrient supplementation on motor impairment; Figure S3. Forest plot of effect of macronutrient supplementation on motor scores; Figure S4. Forest plot of effect of macronutrient supplementation on SBP; Figure S5. Forest plot of effect of macronutrient supplementation on DBP; Figure S6. Forest plot of effect of macronutrient supplementation on MAP; Figure S7. Forest plot of effect of macronutrient supplementation on triglyceride concentrations; Figure S8. Forest plot of effect of macronutrient supplementation on cholesterol concentration (IPD analysis); Figure S9. Forest plot of effect of macronutrient supplementation on HDL; Figure S10. Forest plot of effect of macronutrient supplementation on LDL; Figure S11. Forest plot of effect of macronutrient supplementation on fasting blood glucose concentration; Figure S12. Forest plot of effect of macronutrient supplementation on fasting insulin concentration; Figure S13. Forest plot of effect of macronutrient supplementation on BMI; Figure S14. Forest plot of effect of macronutrient supplementation on BMI z-scores (IPD analysis); Figure S15. Forest plot of effect of macronutrient supplementation on IGF-I concentration (IPD analysis); Figure S16. IPD analysis of secondary developmental outcomes separated for boys and girls; Figure S17. IPD analysis of blood pressure separated for boys and girls; Figure S18. IPD analysis of metabolic outcomes separated for boys and girls; Figure S19. IPD analysis of metabolic outcomes separated for boys and girls; Figure S20. IPD analysis of metabolic outcomes separated for boys and girls; Table S1. Risk of bias within studies; Table S2. Subgroup analyses of size for gestation of the infant; Table S3. Subgroup analyses of size of infant at birth; Table S4. Subgroup analyses of gestational age of infant at birth; Table S5. Subgroup analyses of timing of supplements; Table S6. Subgroup analyses of type of supplement; Table S7. Subgroup analyses of primary milk feed; Table S8. Subgroup analyse of different epochs; Table S9. Search strategies; Table S10. Definitions for primary outcome of metabolic risk; Table S11. Definitions for secondary outcomes.
