# Supplementary material for: Sex-Specific Effects of Nutritional Supplements for Infants Born Early or Small: An Individual Participant Data Meta-Analysis (ESSENCE IPD-MA) I—Cognitive Function and Metabolic Risk
Source: Nutrients. 2022 Jan 18;14(3):418. doi: 10.3390/nu14030418 (PMC8838132; doi:10.3390/nu14030418)
Supplement: Supplementary file 1 [file nutrients-14-00418-s001.zip › nutrients-1500540-supplementary.pdf]

**Supporting information captions**

Text S1. PRISMA-IPD Checklist of items to include when reporting a systematic review and meta-analysis of individual participant data (IPD)
Text S2. Comparison of macronutrient intakes
Figure S1. Forest plot of effect of macronutrient supplementation on cognitive scores
Figure S2. Forest plot of effect of macronutrient supplementation on motor impairment
Figure S3. Forest plot of effect of macronutrient supplementation on motor scores
Figure S4. Forest plot of effect of macronutrient supplementation on SBP
Figure S5. Forest plot of effect of macronutrient supplementation on DBP
Figure S6. Forest plot of effect of macronutrient supplementation on MAP
Figure S7. Forest plot of effect of macronutrient supplementation on triglyceride concentrations
Figure S8. Forest plot of effect of macronutrient supplementation on cholesterol concentration (IPD analysis)
Figure S9. Forest plot of effect of macronutrient supplementation on HDL
Figure S10. Forest plot of effect of macronutrient supplementation on LDL
Figure S11. Forest plot of effect of macronutrient supplementation on fasting blood glucose concentration
Figure S12. Forest plot of effect of macronutrient supplementation on fasting insulin concentration
Figure S13. Forest plot of effect of macronutrient supplementation on BMI
Figure S14. Forest plot of effect of macronutrient supplementation on BMI z-scores (IPD analysis)
Figure S15. Forest plot of effect of macronutrient supplementation on IGF-I concentration (IPD analysis)
Figure S16. IPD analysis of secondary developmental outcomes separated for boys and girls
Figure S17. IPD analysis of blood pressure separated for boys and girls
Figure S18. IPD analysis of metabolic outcomes separated for boys and girls
Figure S19. IPD analysis of metabolic outcomes separated for boys and girls
Figure S20. IPD analysis of metabolic outcomes separated for boys and girls
Table S1. Risk of bias within studies
Table S2. Subgroup analyses of size for gestation of the infant
Table S3. Subgroup analyses of size of infant at birth
Table S4. Subgroup analyses of gestational age of infant at birth
Table S5. Subgroup analyses of timing of supplements
Table S6. Subgroup analyses of type of supplement
Table S7. Subgroup analyses of primary milk feed
Table S8. Subgroup analyse of different epochs
Table S9. Search strategies
Table S10. Definitions for primary outcome of metabolic risk
Table S11. Definitions for secondary outcomes

**Text S1. PRISMA-IPD Checklist of items to include when reporting a systematic review and meta-analysis of individual participant data (IPD)**

| PRISMA-IPD Section/topic | Item No | Checklist item | Reported on page |
|---|---|---|---|
| **Title** | | | |
| Title | 1 | Identify the report as a systematic review and meta-analysis of individual participant data. | Title page |
| **Abstract** | | | |
| Structured summary | 2 | Provide a structured summary including as applicable: | Abstract, Title page |
| | | **Background**: state research question and main objectives, with information on participants, interventions, comparators and outcomes. | |
| | | **Methods**: report eligibility criteria; data sources including dates of last bibliographic search or elicitation, noting that IPD were sought; methods of assessing risk of bias. | |
| | | **Results**: provide number and type of studies and participants identified and number (%) obtained; summary effect estimates for main outcomes (benefits and harms) with confidence intervals and measures of statistical heterogeneity. Describe the direction and size of summary effects in terms meaningful to those who would put findings into practice. | |
| | | **Discussion:** state main strengths and limitations of the evidence, general interpretation of the results and any important implications. | |
| | | **Other:** report primary funding source, registration number and registry name for the systematic review and IPD meta-analysis. | |
| Introduction | | | |
| Rationale | 3 | Describe the rationale for the review in the context of what is already known. | Introduction: paragraph 1-2 |
| Objectives | 4 | Provide an explicit statement of the questions being addressed with reference, as applicable, to participants, interventions, comparisons, outcomes and study design (PICOS). Include any hypotheses that relate to particular types of participant-level subgroups. | Introduction: paragraph 3 |
| Methods | | | |
| Protocol and registration | 5 | Indicate if a protocol exists and where it can be accessed. If available, provide registration information including registration number and registry name. Provide publication details, if applicable. | Methods: paragraph 1 |
| Eligibility criteria | 6 | Specify inclusion and exclusion criteria including those relating to participants, interventions, comparisons, outcomes, study design and characteristics (e.g. years when conducted, required minimum follow-up). Note whether these were applied at the study or individual level i.e. whether eligible participants were included (and ineligible participants excluded) from a study that included a wider population than specified by the review inclusion criteria. The rationale for criteria should be stated. | Methods: 2.2 |
| Identifying studies - information sources | 7 | Describe all methods of identifying published and unpublished studies including, as applicable: which bibliographic databases were searched with dates of coverage; details of any hand searching including of conference proceedings; use of study registers and agency | Methods: 2.1 |

| | | | |
|---|---|---|---|
| | | or company databases; contact with the original research team and experts in the field; open adverts and surveys. Give the date of last search or elicitation. | |
| Identifying studies - search | 8 | Present the full electronic search strategy for at least one database, including any limits used, such that it could be repeated. | Table S9 |
| Study selection processes | 9 | State the process for determining which studies were eligible for inclusion. | Methods: 2.2 |
| Data collection processes | 10 | Describe how IPD were requested, collected and managed, including any processes for querying and confirming data with investigators. If IPD were not sought from any eligible study, the reason for this should be stated (for each such study). | Methods: 2.2, 2.4, 2.5 |
| | | If applicable, describe how any studies for which IPD were not available were dealt with. This should include whether, how and what aggregate data were sought or extracted from study reports and publications (such as extracting data independently in duplicate) and any processes for obtaining and confirming these data with investigators. | |
| Data items | 11 | Describe how the information and variables to be collected were chosen. List and define all study level and participant level data that were sought, including baseline and follow-up information. If applicable, describe methods of standardising or translating variables within the IPD datasets to ensure common scales or measurements across studies. | Methods: 2.2, 2.4, 2.5 |
| IPD integrity | A1 | Describe what aspects of IPD were subject to data checking (such as sequence generation, data consistency and completeness, baseline imbalance) and how this was done. | Methods: 2.3 |
| Risk of bias assessment in individual studies. | 12 | Describe methods used to assess risk of bias in the individual studies and whether this was applied separately for each outcome. If applicable, describe how findings of IPD checking were used to inform the assessment. Report if and how risk of bias assessment was used in any data synthesis. | Methods: 2.3 |
| Specification of outcomes and effect measures | 13 | State all treatment comparisons of interests. State all outcomes addressed and define them in detail. State whether they were pre-specified for the review and, if applicable, whether they were primary/main or secondary/additional outcomes. Give the principal measures of effect (such as risk ratio, hazard ratio, difference in means) used for each outcome. | Methods: 2.2, 2.4, 2.5 |
| Synthesis methods | 14 | Describe the meta-analysis methods used to synthesise IPD. Specify any statistical methods and models used. Issues should include (but are not restricted to):<br>• Use of a one-stage or two-stage approach.<br>• How effect estimates were generated separately within each study and combined across studies (where applicable).<br>• Specification of one-stage models (where applicable) including how clustering of patients within studies was accounted for.<br>• Use of fixed or random effects models and any other model assumptions, such as proportional hazards.<br>• How (summary) survival curves were generated (where applicable).<br>• Methods for quantifying statistical heterogeneity (such as $I^2$ and $\tau^2$).<br>• How studies providing IPD and not providing IPD were analysed together (where applicable).<br>• How missing data within the IPD were dealt with (where applicable). | Methods: 2.4, 2.5 |
| Exploration of variation in effects | A2 | If applicable, describe any methods used to explore variation in effects by study or participant level characteristics (such as estimation of interactions between effect and covariates). State all participant-level characteristics that were analysed as potential effect modifiers, and whether these were pre-specified. | Methods: 2.4, 2.5 |
| Risk of bias across studies | 15 | Specify any assessment of risk of bias relating to the accumulated body of evidence, including any pertaining to not obtaining IPD for particular studies, outcomes or other variables. | Methods: 2.3 |

| | | | |
|---|---|---|---|
| Additional analyses | 16 | Describe methods of any additional analyses, including sensitivity analyses. State which of these were pre-specified. | Methods: 2.4, 2.5 |
| **Results** | | | |
| Study selection and IPD obtained | 17 | Give numbers of studies screened, assessed for eligibility, and included in the systematic review with reasons for exclusions at each stage. Indicate the number of studies and participants for which IPD were sought and for which IPD were obtained. For those studies where IPD were not available, give the numbers of studies and participants for which aggregate data were available. Report reasons for non-availability of IPD. Include a flow diagram. | Results: 3.1, Figure 1 |
| Study characteristics | 18 | For each study, present information on key study and participant characteristics (such as description of interventions, numbers of participants, demographic data, unavailability of outcomes, funding source, and if applicable duration of follow-up). Provide (main) citations for each study. Where applicable, also report similar study characteristics for any studies not providing IPD. | Table 1 |
| IPD integrity | A3 | Report any important issues identified in checking IPD or state that there were none. | Results: 3.1 |
| Risk of bias within studies | 19 | Present data on risk of bias assessments. If applicable, describe whether data checking led to the up-weighting or down-weighting of these assessments. Consider how any potential bias impacts on the robustness of meta-analysis conclusions. | Results: 3.2 Table S1 |
| Results of individual studies | 20 | For each comparison and for each main outcome (benefit or harm), for each individual study report the number of eligible participants for which data were obtained and show simple summary data for each intervention group (including, where applicable, the number of events), effect estimates and confidence intervals. These may be tabulated or included on a forest plot. | Results: 3.3-3.6, Figure 2, Figure 3, Figure S1-S15 |
| Results of syntheses | 21 | Present summary effects for each meta-analysis undertaken, including confidence intervals and measures of statistical heterogeneity. State whether the analysis was pre-specified, and report the numbers of studies and participants and, where applicable, the number of events on which it is based. | Results: 3.3-3.7, Figure 2, Figure 3, Figure S1-S15 |
| | | When exploring variation in effects due to patient or study characteristics, present summary interaction estimates for each characteristic examined, including confidence intervals and measures of statistical heterogeneity. State whether the analysis was pre-specified. State whether any interaction is consistent across trials. | |
| | | Provide a description of the direction and size of effect in terms meaningful to those who would put findings into practice. | |
| Risk of bias across studies | 22 | Present results of any assessment of risk of bias relating to the accumulated body of evidence, including any pertaining to the availability and representativeness of available studies, outcomes or other variables. | Results: 3.2 Table S1 |
| Additional analyses | 23 | Give results of any additional analyses (e.g. sensitivity analyses). If applicable, this should also include any analyses that incorporate aggregate data for studies that do not have IPD. If applicable, summarise the main meta-analysis results following the inclusion or exclusion of studies for which IPD were not available. | Results: 3.3-3.7, Figure 2, Figure 3, Figure S1-S20, Table S2-S8 |
| **Discussion** | | | |

| | | | |
|---|---|---|---|
| Summary of evidence | 24 | Summarise the main findings, including the strength of evidence for each main outcome. | Discussion: paragraph 1-2, Figure 5-6 |
| Strengths and limitations | 25 | Discuss any important strengths and limitations of the evidence including the benefits of access to IPD and any limitations arising from IPD that were not available. | Discussion: paragraph 12-13 |
| Conclusions | 26 | Provide a general interpretation of the findings in the context of other evidence. | Discussion: paragraph 14 |
| Implications | A4 | Consider relevance to key groups (such as policy makers, service providers and service users). Consider implications for future research. | Discussion: paragraph 14 |
| **Funding** | | | |
| Funding | 27 | Describe sources of funding and other support (such as supply of IPD), and the role in the systematic review of those providing such support. | Funding, acknowledge, author contribution |

A1 – A3 denote new items that are additional to standard PRISMA items. A4 has been created as a result of re-arranging content of the standard PRISMA statement to suit the way that systematic review IPD meta-analyses are reported.

© Reproduced with permission of the PRISMA IPD Group, which encourages sharing and reuse for non-commercial purpose

**Text S2. Comparison of macronutrient intake**

**a. Macronutrient intakes between trials using formula as primary feed and breast milk as primary feed.**

To explore whether the differences in effects of supplements between infants receiving breast milk or formula as their primary feed were due to different baseline macronutrient intakes or quantity of supplements, we compared the mean macronutrient intakes in the unsupplemented groups receiving breast milk or formula as their primary feed, and the mean difference in intakes between supplemented and unsupplemented groups. Infants in the unsupplemented group who received breastmilk as their primary feed had similar protein, fat, carbohydrate and energy intakes to those whose primary feed was formula. However, amongst infants who received breastmilk as their primary feed, those in the supplemented group received more protein, energy and carbohydrate than those in unsupplemented group, whereas amongst infants who received formula as their primary feed, the supplemented formula group received much smaller increases in protein, energy and carbohydrate than the unsupplemented group.

| | Breast milk | | Formula | | |
|---|---|---|---|---|---|
| | Mean | SD | Mean | SD | P Value |
| **Mean intakes in the unsupplemented groups** | | | | | |
| Protein (g/100 ml) | 1·43 | 0·24 | 1·64 | 0·33 | 0·26 |
| Fat (g/100 ml) | 4 | 0·49 | 3·94 | 0·39 | 0·84 |
| Carbohydrate (g/100 ml) | 6·53 | 2·33 | 7·26 | 0·45 | 0·49 |
| Energy (g/100 ml) | 68 | 5·29 | 70·17 | 4·92 | 0·48 |
| **Mean differences intakes between supplemented and unsupplemented groups** | | | | | |
| Protein (g/100 ml) | 0·92 | 0·49 | 0·46 | 0·15 | 0·07 |
| Fat (g/100 ml) | 0·06 | 0·73 | 0·14 | 0·21 | 0·84 |
| Carbohydrate (g/100 ml) | 2·15 | 0·46 | 0·24 | 0·17 | 0·0001 |
| Energy (g/100 ml) | 11·5 | 6·89 | 5·17 | 3·37 | 0·07 |
| The composition information for formulae were from IPD or extracted from the publications, and the composition of breastmilk was from IPD or estimated according to the recent guideline[1]. | | | | | |

**b. Macronutrient intakes between trials conducted up to 2000 and those conducted after 2000.**

To explore whether the differences in effects of supplements between trials conducted before and during 2000 or after 2000 were due to gradual increases in baseline macronutrient intakes over time, we compared the mean macronutrient intakes in the unsupplemented groups in trials conducted before or after 2000, and the mean differences in intakes between supplemented and unsupplemented groups. This showed that there were no differences between the two epochs in mean baseline intakes or in mean differences in intake between supplemented and unsupplemented groups for protein, fat, carbohydrate or energy.

| | Before and during 2000 | | After 2000 | | |
|---|---|---|---|---|---|
| | Mean | SD | Mean | SD | P Value |
| **Mean intakes in the unsupplemented groups** | | | | | |
| Protein (g/100 ml) | 1·46 | 0·15 | 1·58 | 0·38 | 0·52 |
| Fat (g/100 ml) | 3·87 | 0·25 | 4·05 | 0·53 | 0·54 |
| Carbohydrate (g/100 ml) | 7·03 | 0·12 | 6·68 | 2·39 | 0·78 |
| Energy (g/100 ml) | 68 | 1·1 | 70·17 | 7·14 | 0·48 |
| **Mean differences intakes between supplemented and unsupplemented groups** | | | | | |
| Protein (g/100 ml) | 0·54 | 0·15 | 0·86 | 0·55 | 0·23 |
| Fat (g/100 ml) | 0·18 | 0·15 | 0·03 | 0·73 | 0·69 |

| | | | | | |
|---|---|---|---|---|---|
| Carbohydrate (g/100 ml) | 0·92 | 1·21 | 1·6 | 0·95 | 0·39 |
| Energy (g/100 ml) | 7·5 | 3·89 | 9·17 | 8·13 | 0·66 |
| The composition information for formulae were from IPD or extracted from the publications, and the composition of breastmilk was from IPD or estimated according to the recent guideline[1]. | | | | | |

**References**


1. National Health & Medical Research Council (NHMRC). Dietary guidelines for children and adolescents in Australia - incorporating the infant feeding guidelines for health workers. Australia: The National Health and Medical Research Council; 2003 [updated 10 April 2003; cited 2019 17 June ]. Available from: http://childaustralia.mrooms.net/pluginfile.php/4134/mod_page/content/38/diet-guidelines.pdf

a.

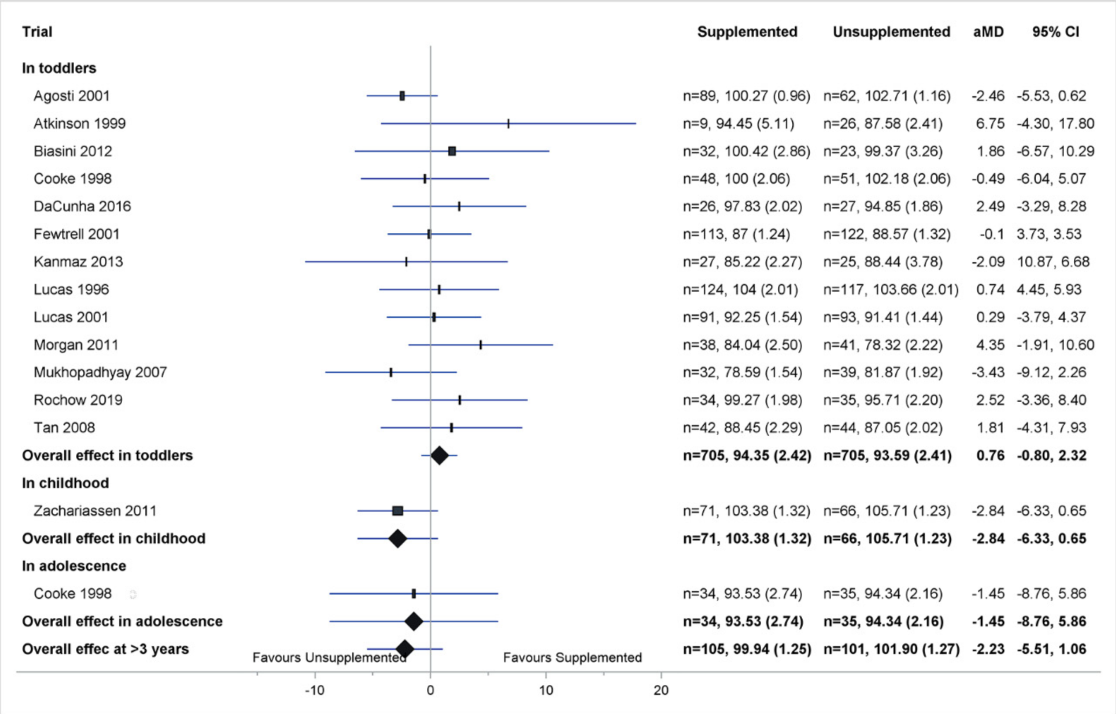


b.

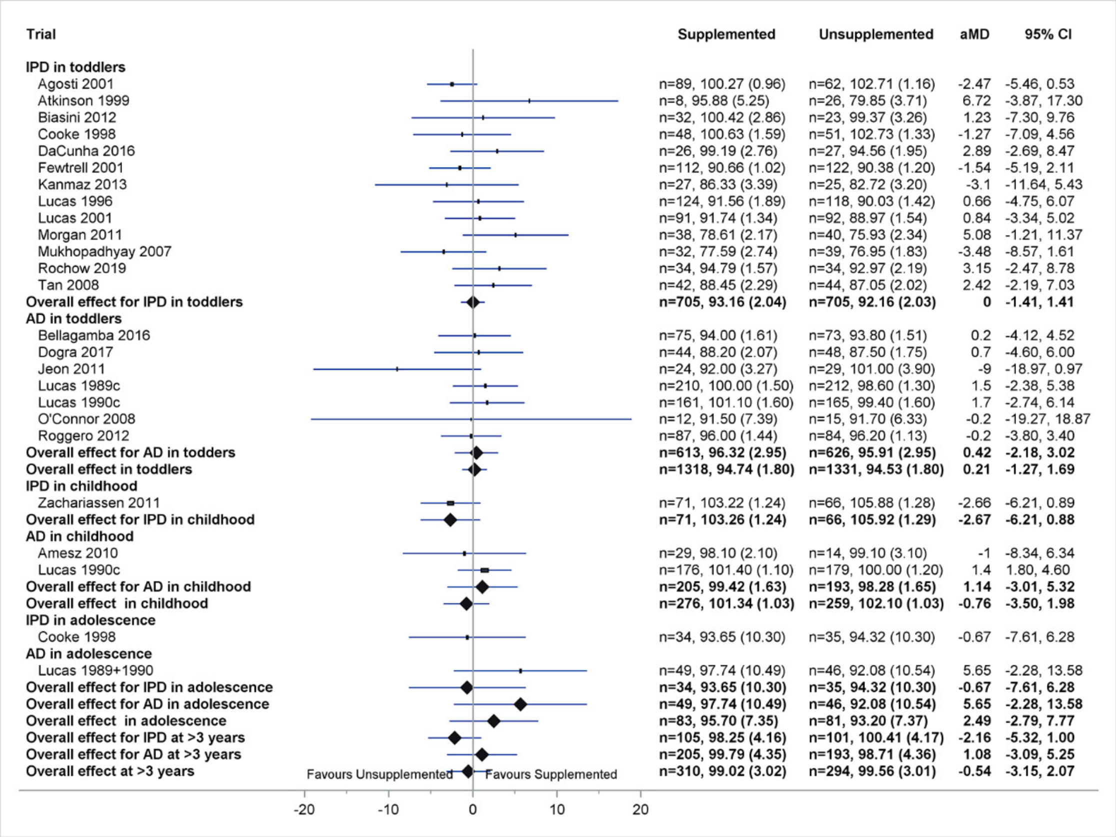


**Figure S1. Forest plot of effect of macronutrient supplementation on cognitive scores.** a. IPD analysis, b. Combined IPD and AD analysis. Data are mean and standard error, with adjusted mean difference (aMD) for treatment effect and 95% confidence intervals (CIs) adjusting for sex, gestational age and birthweight z-scores. The box size of point estimate is proportional to inverse variance. Heterogeneity of IPD analysis in toddlers p=0.77, $tau^2$=0.63; at >3 years p=0.69, $tau^2$=2.66. Heterogeneity of combined IPD and AD analysis in toddlers $tau^2$=0.52, in childhood $tau^2$=1.74, in adolescence $tau^2$=6.97, at >3 years $tau^2$=2.66. IPD, individual participant data; AD, aggregated data.

a.

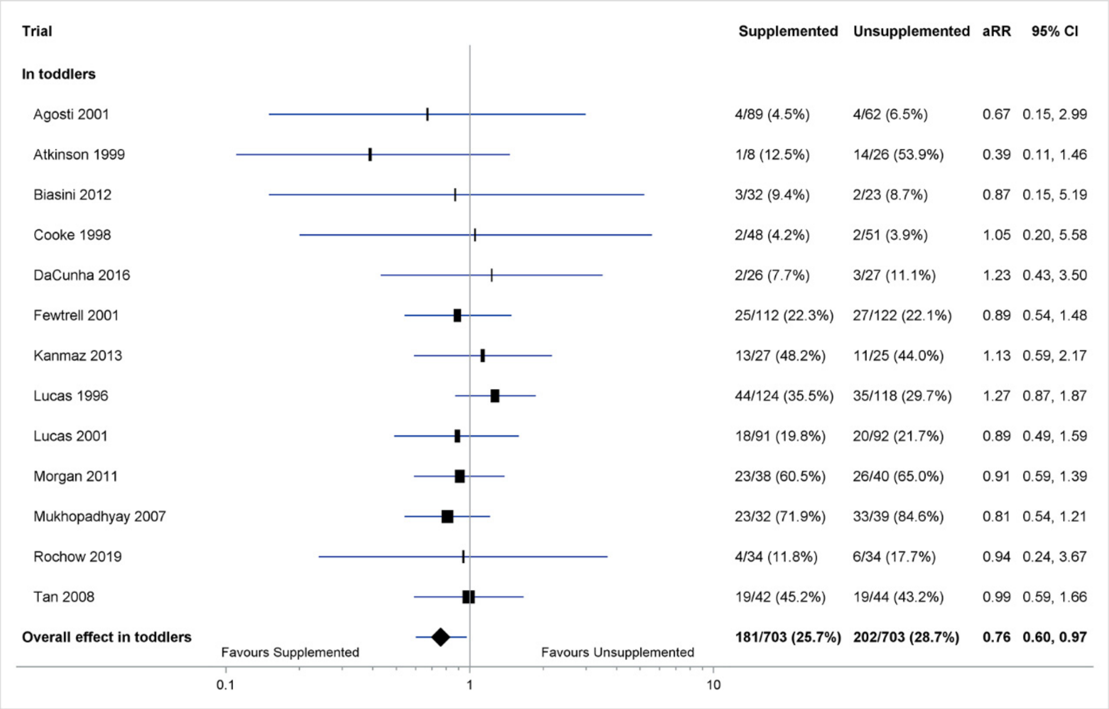


b.

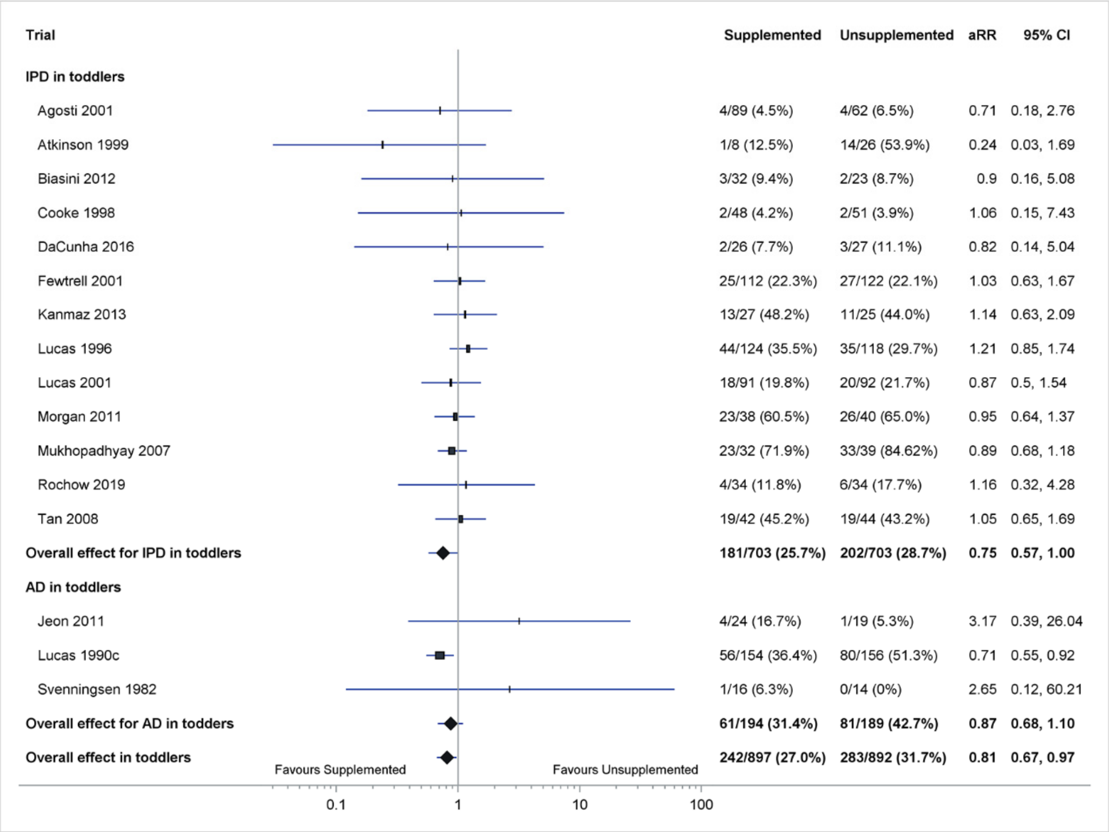


**Figure S2. Forest plot of effect of macronutrient supplementation on motor impairment.** a. IPD analysis, b. Combined IPD and AD analysis. Data are numbers (percentages) with adjusted relative risk (aRR) and 95% confidence intervals (CIs) for treatment effect adjusting for sex, gestational age and birthweight z-scores. The box size of point estimate is proportional to inverse variance. Heterogeneity: IPD analysis in toddlers p=0.87, $tau^2$=0.02; Combined IPD and AD analysis in toddlers $tau^2$=0.01. IPD, individual participant data; AD, aggregated data.

a.

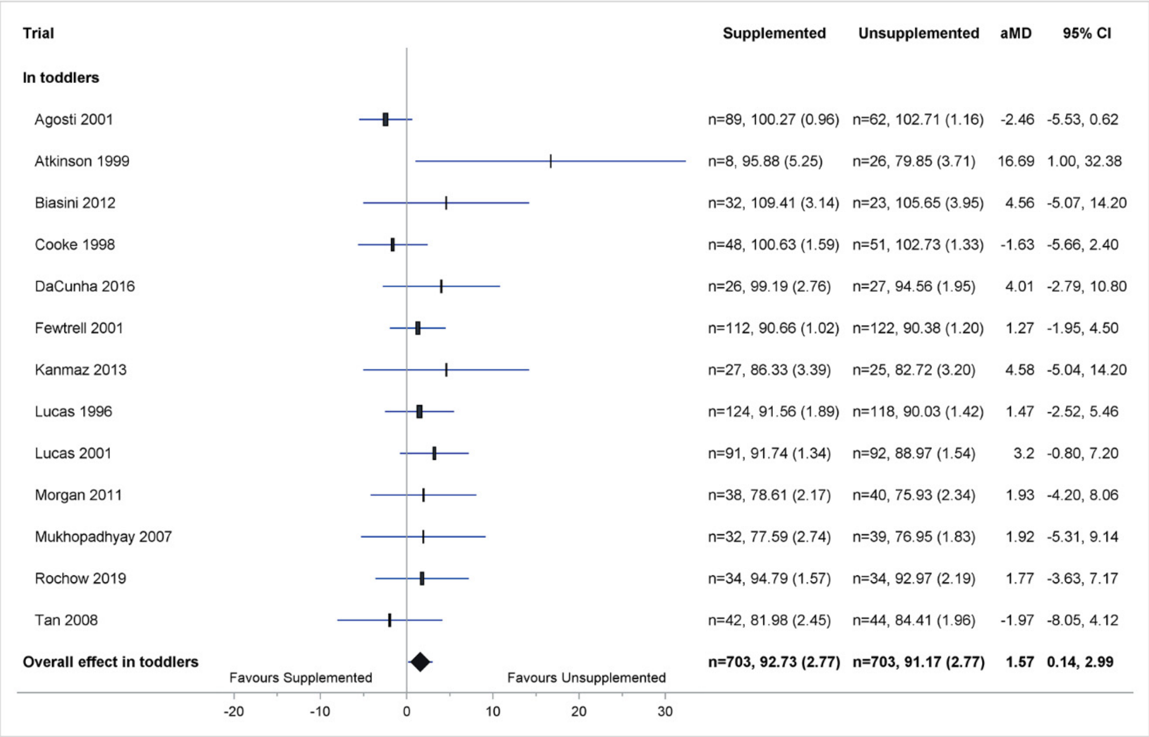


b.

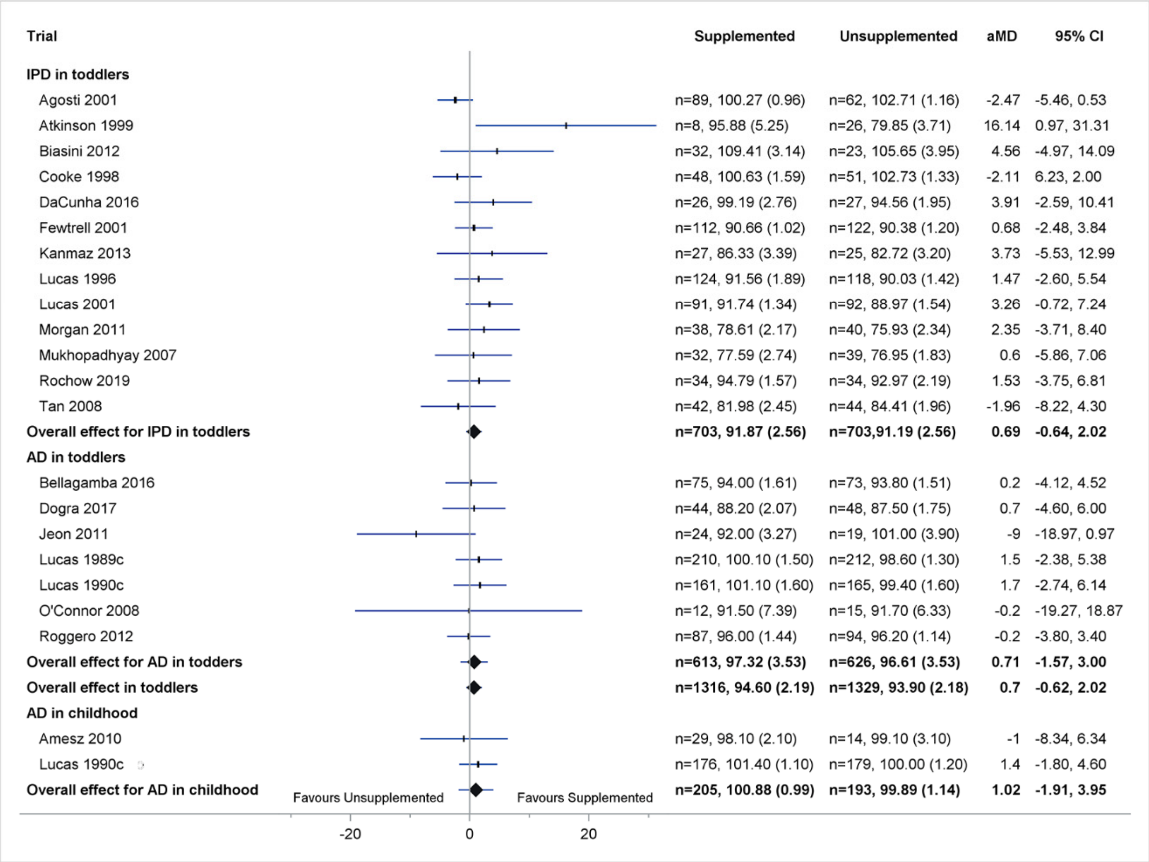


**Figure S3. Forest plot of effect of macronutrient supplementation on motor scores.** a. IPD analysis, b. Combined IPD and AD analysis. Data are mean and standard error, with adjusted mean difference (aMD) for treatment effect and 95% confidence intervals (CIs) adjusting for sex, gestational age and birthweight z-scores. The box size of point estimate is proportional to inverse variance. Heterogeneity: IPD analysis in toddlers p=0.25, $tau^2$=0.53; Combined IPD and AD analysis $tau^2$=0.45. IPD, individual participant data; AD, aggregated data.

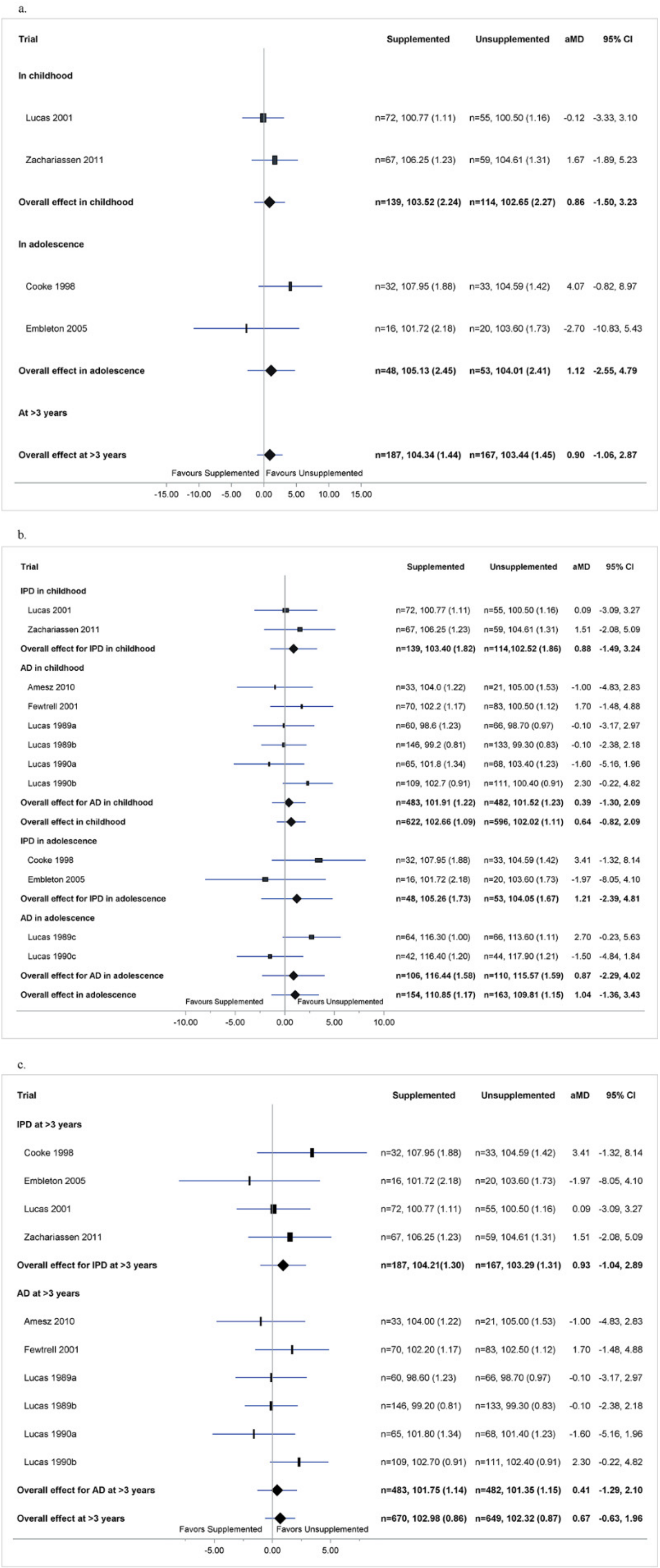


**Figure S4. Forest plot of effect of macronutrient supplementation on SBP.** a. IPD analysis, b. Combined IPD and AD analysis in childhood and in adolescence. C. Combined IPD and AD analysis at >3 years. Data are mean and standard error, with adjusted mean difference (aMD) for treatment effect and 95% confidence intervals (CIs) adjusted for sex, gestational age and birthweight z-scores. The box size of point estimate is proportional to inverse variance. Heterogeneity of IPD analysis in childhood p=0.44, $tau^2$=1.44; in adolescence p=0.10, $tau^2$=3.39; at >3 years p=0.46, $tau^2$=0.98. Heterogeneity of combined IPD and AD analysis in childhood $tau^2$=0.55; in adolescence $tau^2$=1.44; at >3 years $tau^2$=0.44. SBP, systolic blood pressure; IPD, individual participant data; AD, aggregated data.

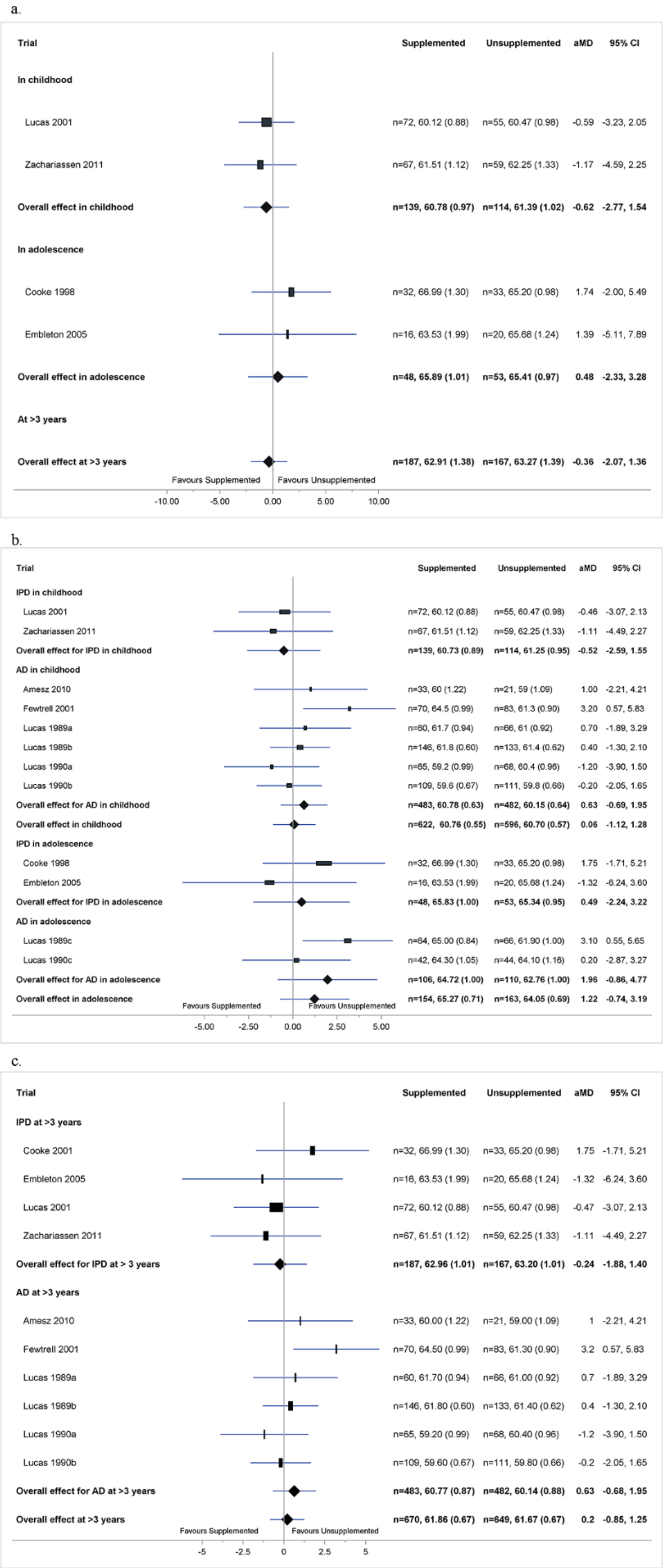


**Figure S5. Forest plot of effect of macronutrient supplementation on DBP.** a. IPD analysis, b. Combined IPD and AD analysis in childhood and in adolescence. C. Combined IPD and AD analysis at >3 years. Data are mean and standard error, with adjusted mean difference (aMD) for treatment effect and 95% confidence intervals (CIs) adjusted for sex, gestational age and birthweight z-scores. The box size of point estimate is proportional to inverse variance. Heterogeneity of IPD analysis in childhood p=0.83, tau²=1.19, in adolescence p=0.32, tau²=1.99; at >3 years p=0.73, tau²=0.76. Heterogeneity of combined IPD and AD analysis in childhood tau²=0.36; in adolescence tau²=0.98; at >3 years tau²=0.25. DBP, diastolic blood pressure; IPD, individual participant data; AD, aggregated data.

a,

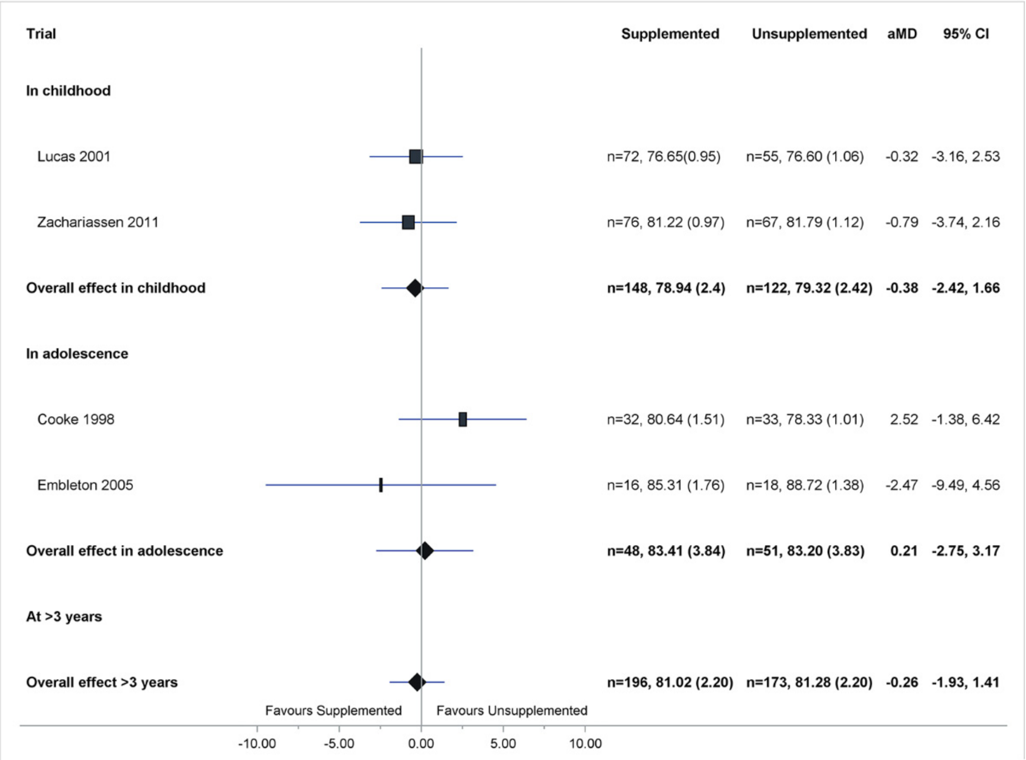


b.

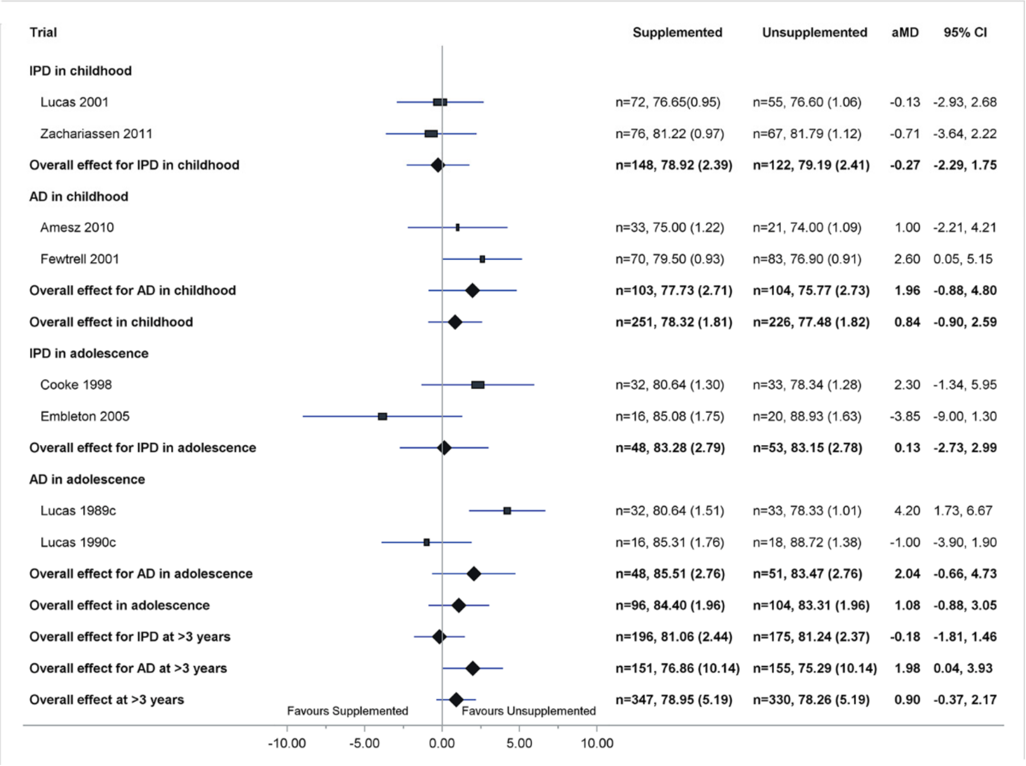


**Figure S6. Forest plot of effect of macronutrient supplementation on MAP.** a. IPD analysis, b. Combined IPD and AD analysis. Data are mean and standard error, with adjusted mean difference (aMD) for treatment effect and 95% confidence intervals (CIs) adjusted for sex, gestational age and birthweight z-scores. The box size of point estimate is proportional to inverse variance. Heterogeneity of IPD analysis in childhood p=0.84, $tau^2$=1.06; in adolescence p= 0.07, $tau^2$=2.22; at >3 years p=0.42, $tau^2$=0.72. Heterogeneity of combined IPD and AD analysis in childhood $tau^2$=0.77; in adolescence $tau^2$=0.98; at >3 years $tau^2$=0.42. MAP, mean arterial pressure; IPD, individual participant data; AD, aggregated data.

a.

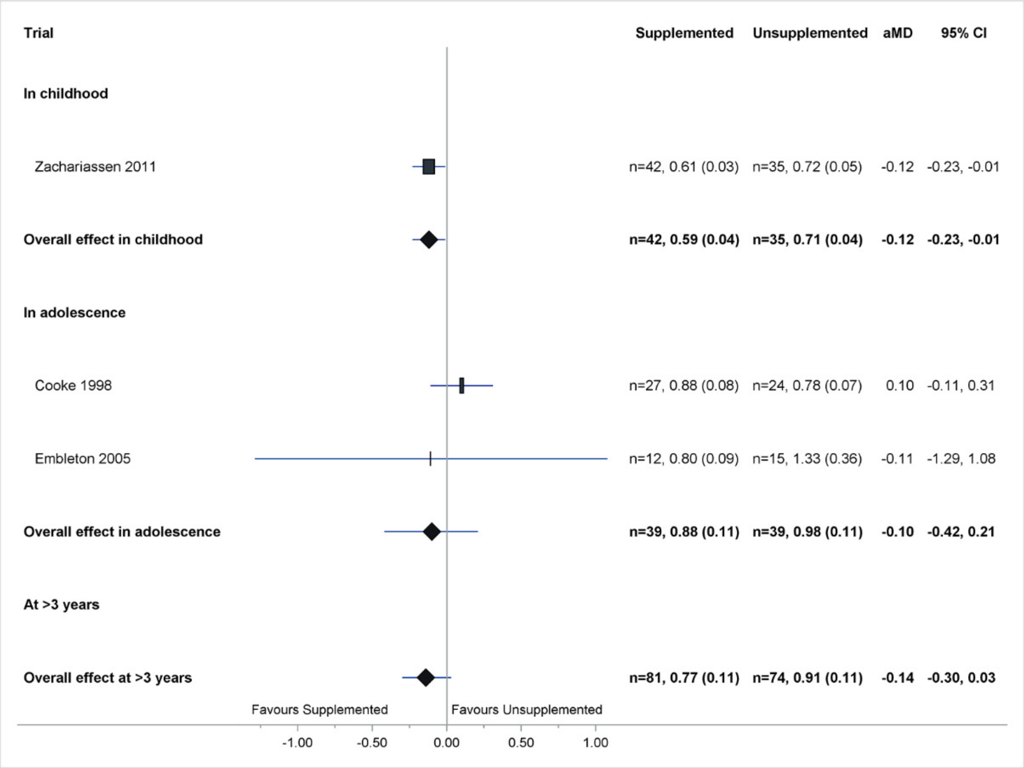


b.

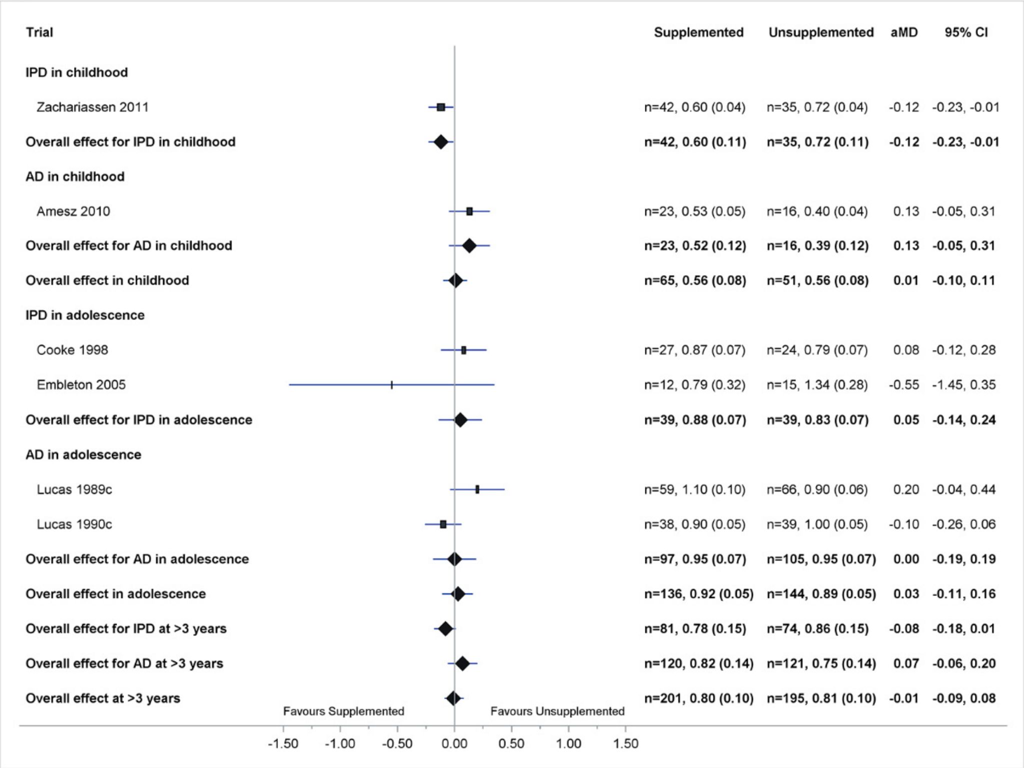


**Figure S7. Forest plot of effect of macronutrient supplementation on triglyceride concentrations.** a. IPD analysis, b. Combined IPD and AD analysis. Data are mean and standard error, with adjusted mean difference (aMD) for treatment effect and 95% confidence intervals (CIs) adjusted for sex, gestational age and birthweight z-scores. The box size of point estimate is proportional to inverse variance. Heterogeneity of IPD analysis: in adolescence = 0.17, $tau^2$=0.03; at >3 years =0.11, $tau^2$=0.01. Heterogeneity of combined IPD and AD analysis in childhood $tau^2$=0.002; in adolescence $tau^2$=0.004; at >3 years $tau^2$=0.002. IPD, individual participant data; AD, aggregated data.

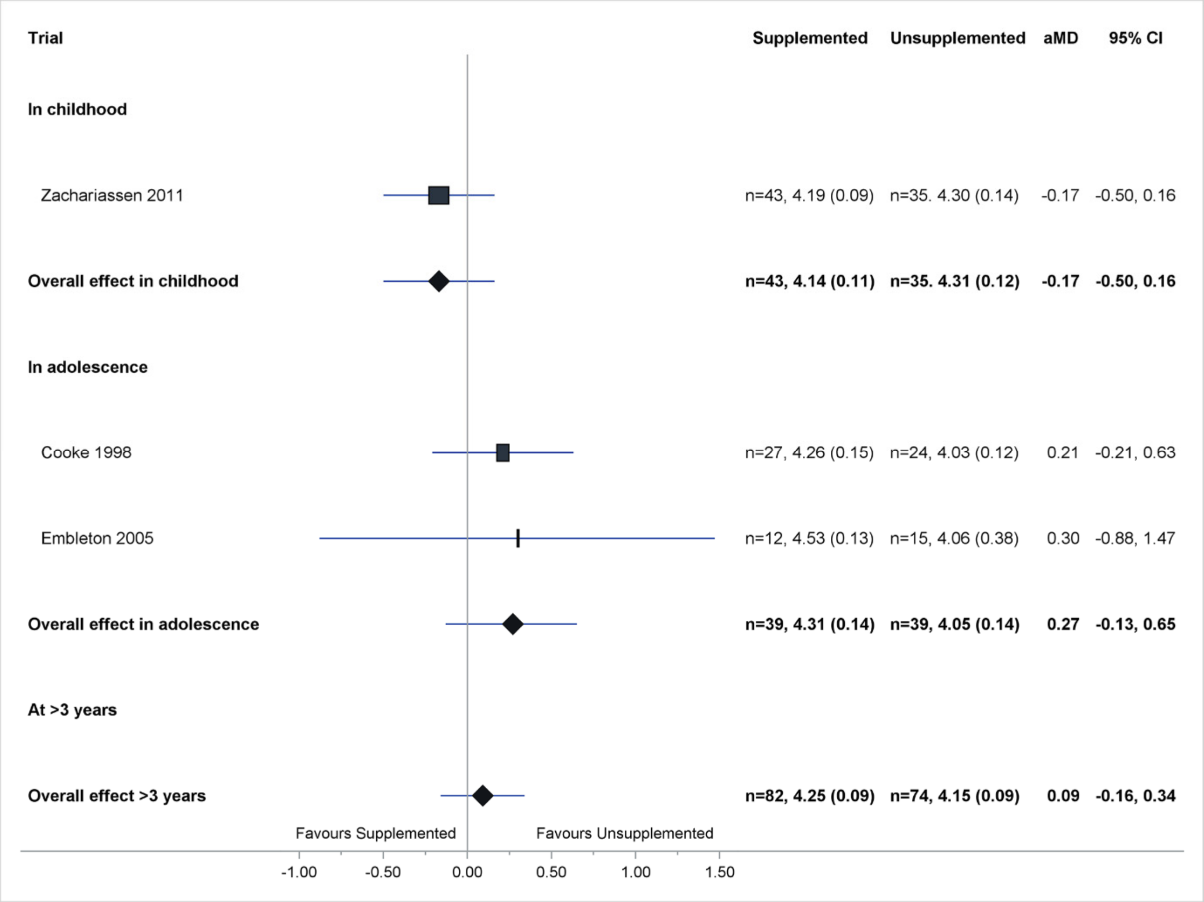


**Figure S8. Forest plot of effect of macronutrient supplementation on cholesterol concentration (IPD analysis).** Data are mean and standard error, with adjusted mean difference (aMD) for treatment effect and 95% confidence intervals (CIs) adjusted for sex, gestational age and birthweight z-scores. The box size of point estimate is proportional to inverse variance. for Heterogeneity in adolescence p=0.73, $tau^2$=0.03; at >3 years p=0.20, $tau^2$=0.032.

a.

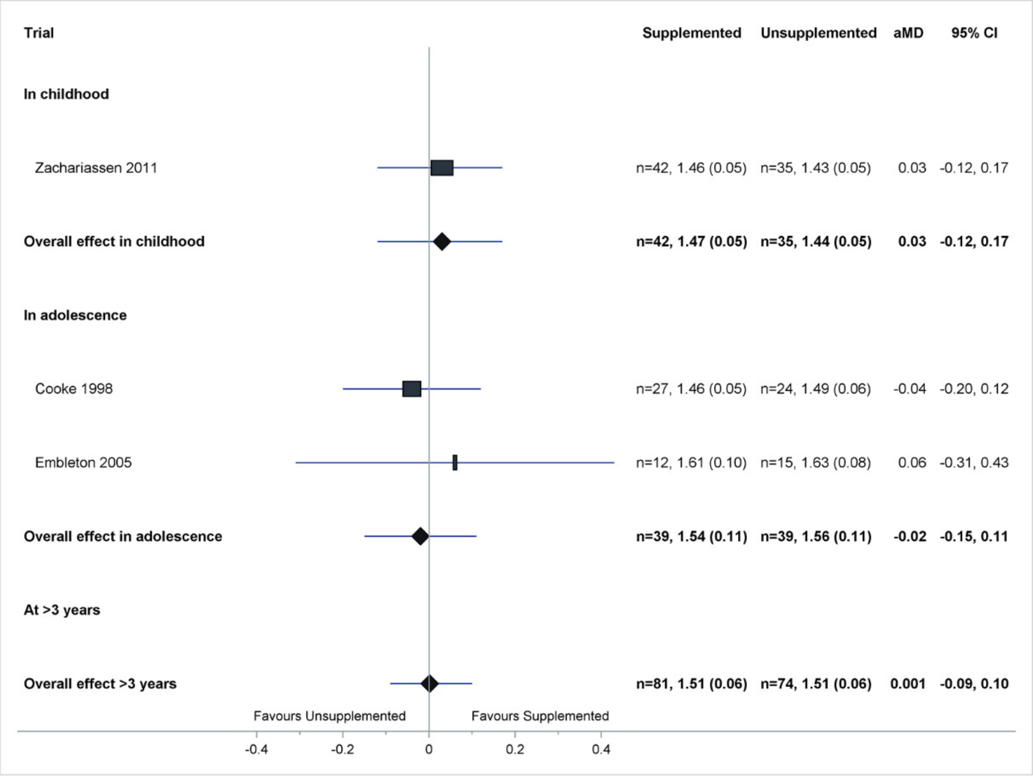


b.

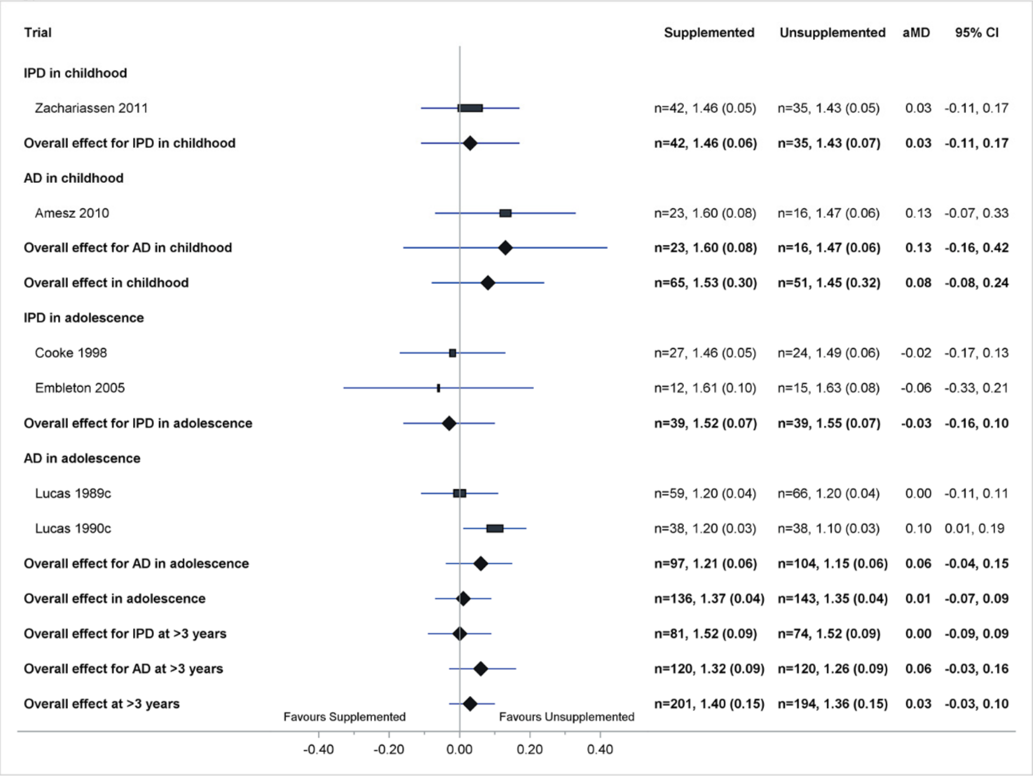


**Figure S9. Forest plot of effect of macronutrient supplementation on HDL.** a. IPD analysis, b. Combined IPD and AD analysis. Data are mean and standard error, with adjusted mean difference (aMD) for treatment effect and 95% confidence intervals (CIs) adjusted for sex, gestational age and birthweight z-scores. The box size of point estimate is proportional to inverse variance. Heterogeneity of IPD analysis in adolescence = 0.77, tau$^2$=0.01; at >3 years p=0.90, tau$^2$=0.003. Heterogeneity of combined IPD and AD analysis in childhood tau$^2$=0.01; in adolescence tau$^2$=0.002; at >3 years tau$^2$=0.001. IPD, individual participant data; AD, aggregated data.

a.

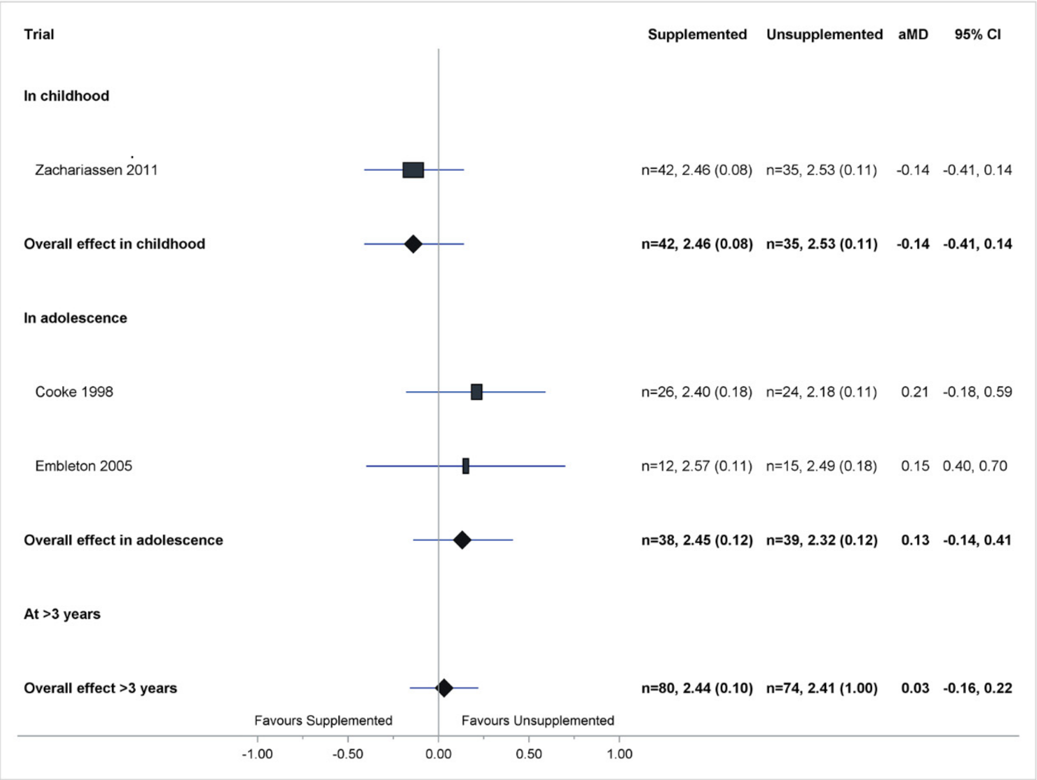


b.

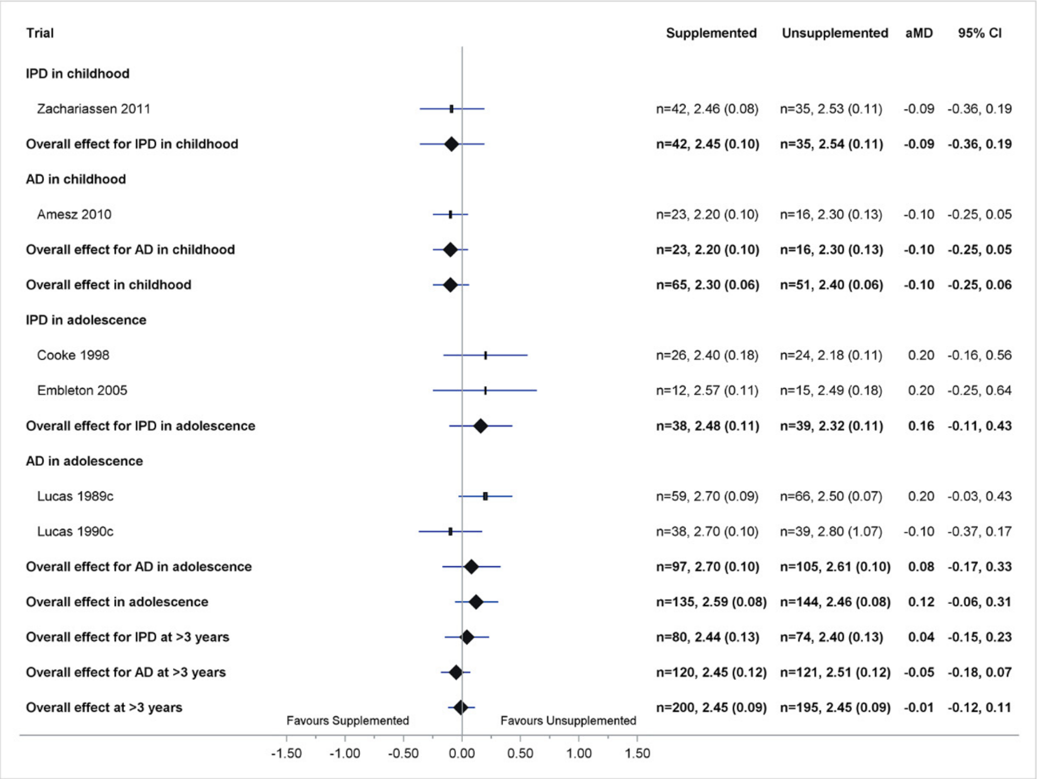


**Figure S10. Forest plot of effect of macronutrient supplementation on LDL.** a. IPD analysis, b. Combined IPD and AD analysis. Data are mean and standard error, with adjusted mean difference (aMD) for treatment effect and 95% confidence intervals (CIs) adjusted for sex, gestational age and birthweight z-scores. The box size of point estimate is proportional to inverse variance. Heterogeneity of IPD analysis in adolescence p=0.47, $tau^2$=0.02; at >3 years p=0.49, $tau^2$=0.01. Heterogeneity of combined IPD and AD analysis in childhood $tau^2$=0.01; in adolescence $tau^2$=0.01; at >3 years $tau^2$=0.003. IPD, individual participant data; AD, aggregated data.

a.

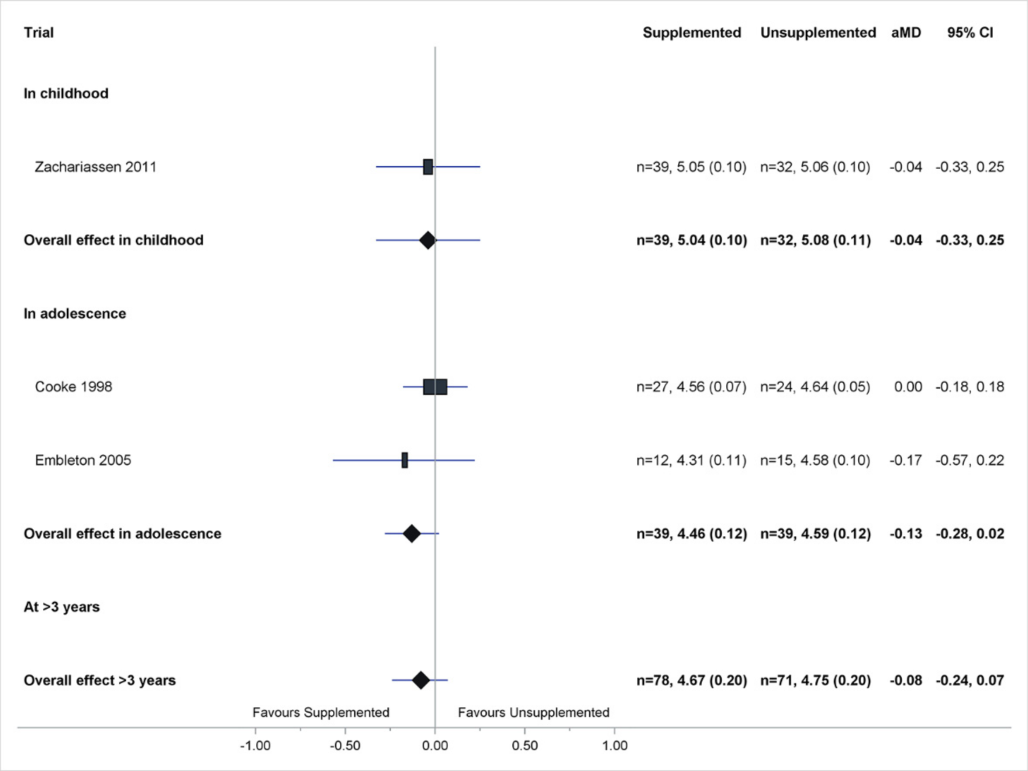


b.

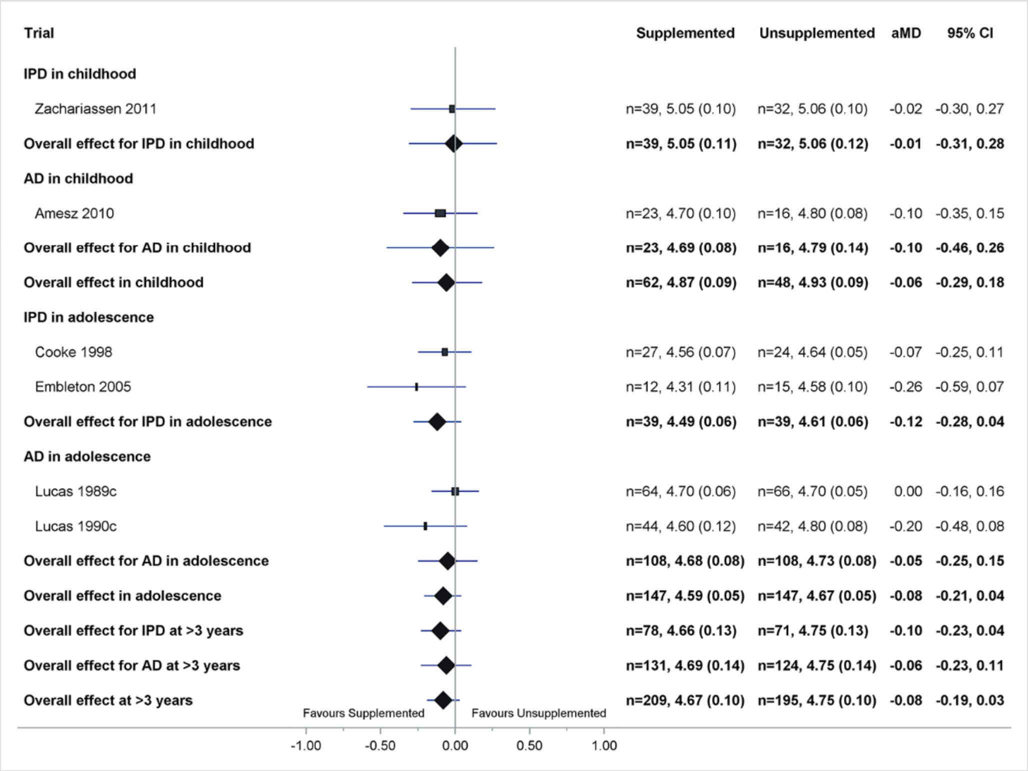


**Figure S11. Forest plot of effect of macronutrient supplementation on fasting blood glucose concentration.** a. IPD analysis, b. Combined IPD and AD analysis. Data are mean and standard error, with adjusted mean difference (aMD) for treatment effect and 95% confidence intervals (CIs) adjusted for sex, gestational age and birthweight z-scores. The box size of point estimate is proportional to inverse variance. Heterogeneity of IPD analysis in adolescence p=0.13, $tau^2$=0.01; at >3 years p=0.62, $tau^2$=0.01. Heterogeneity of combined IPD and AD analysis in childhood $tau^2$=0.01; in adolescence $tau^2$=0.004; at >3 years $tau^2$=0.003. IPD, individual participant data; AD, aggregated data.

a.

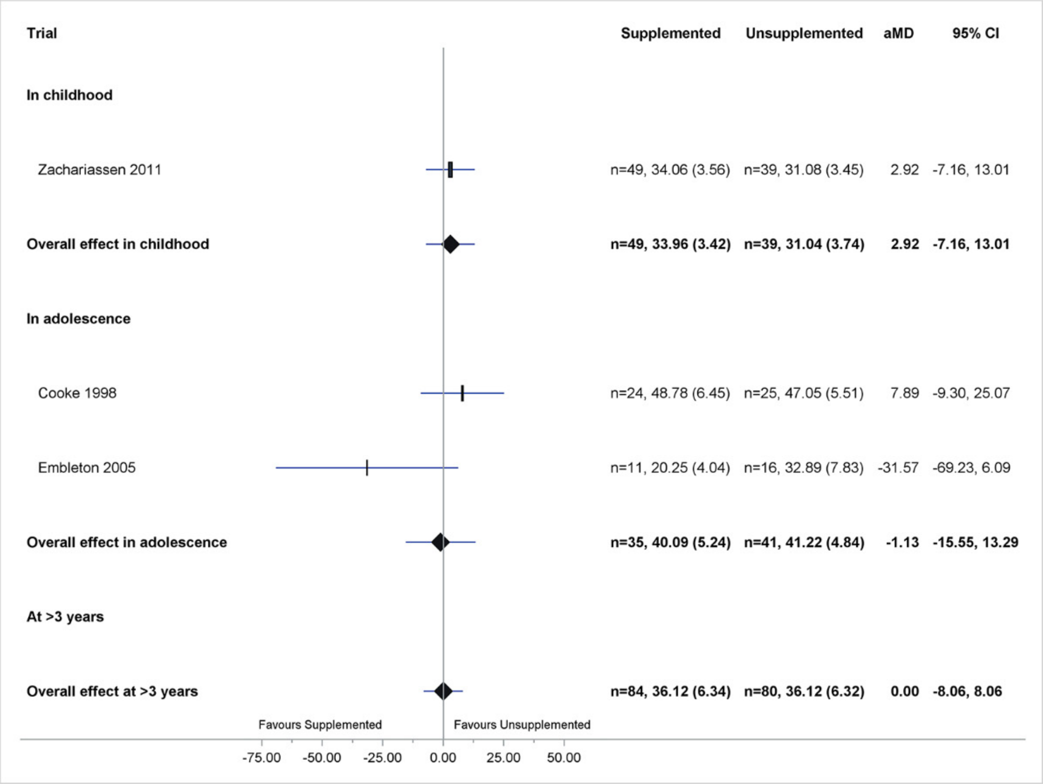


b.

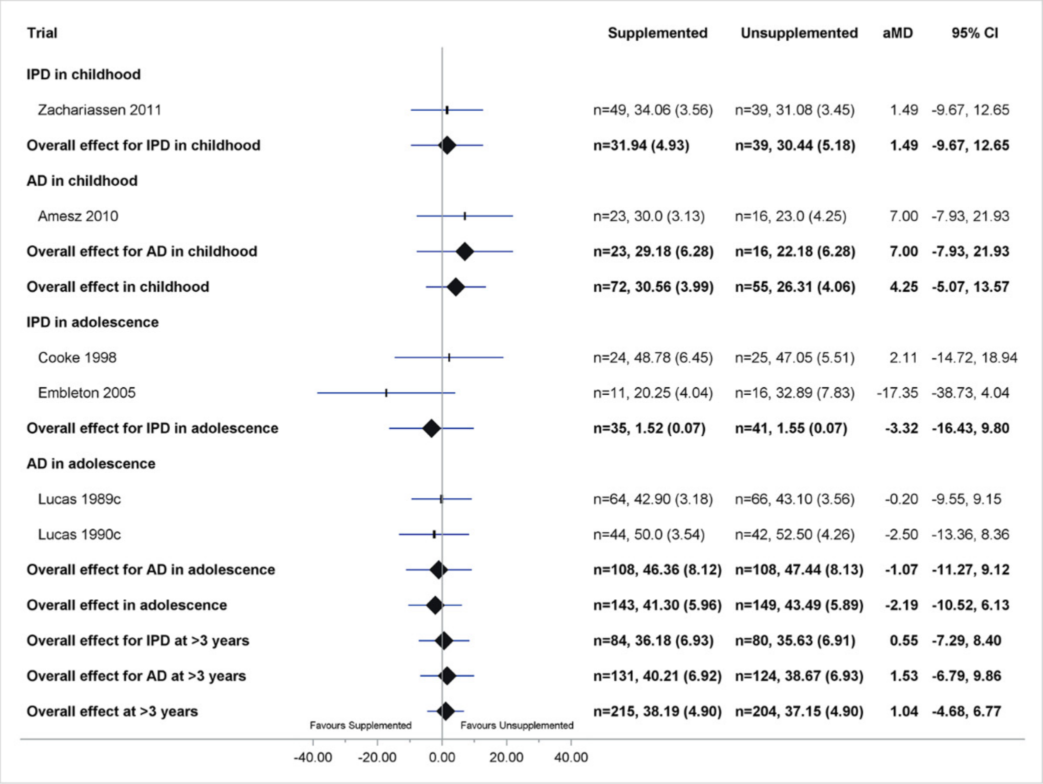


**Figure S12. Forest plot of effect of macronutrient supplementation on fasting insulin concentration.** a. IPD analysis, b. Combined IPD and AD analysis. Data are mean and standard error, with adjusted mean difference (aMD) for treatment effect and 95% confidence intervals (CIs) adjusted for sex, gestational age and birthweight z-scores. The box size of point estimate is proportional to inverse variance. Heterogeneity of IPD analysis heterogeneity in adolescence p=0.29, $tau^2$=46.24, at >3 years p=0.32; $tau^2$=16.13. Heterogeneity of combined IPD and AD analysis in childhood $tau^2$=21.72; in adolescence $tau^2$=17.43; at >3 years $tau^2$=8.41. IPD, individual participant data; AD, aggregated data.

a.

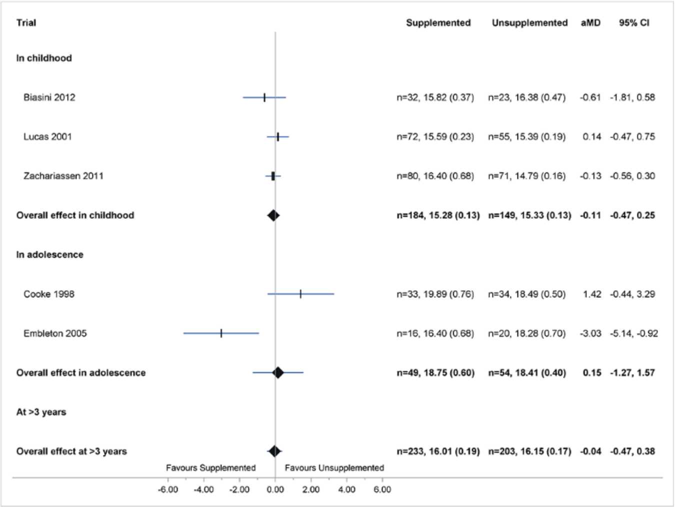


b.

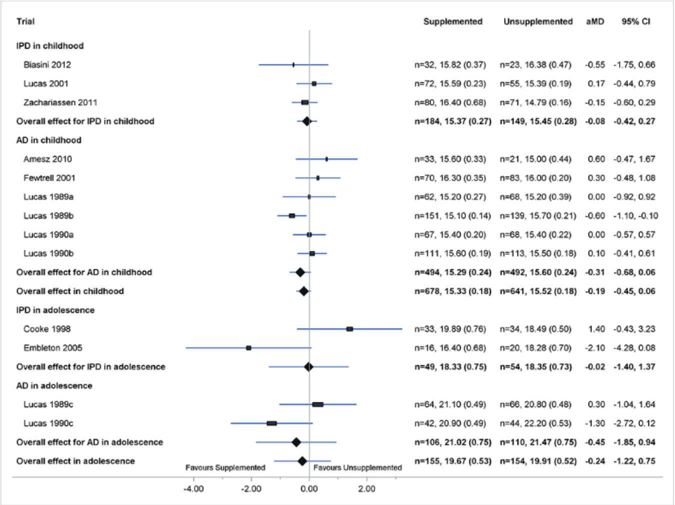


c.

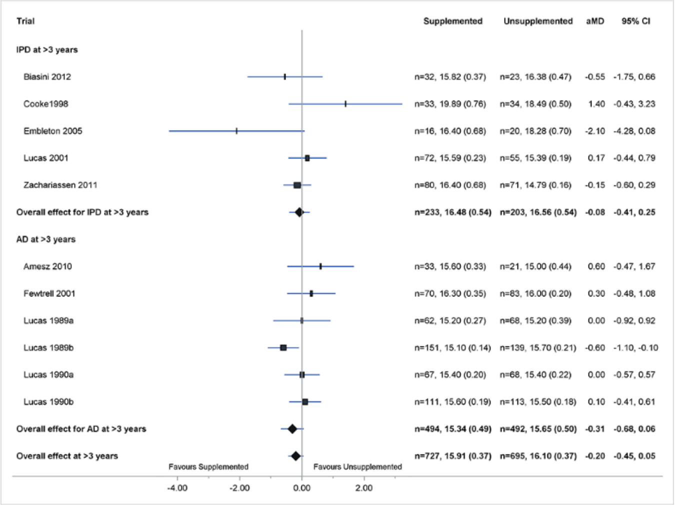


**Figure S13. Forest plot of effect of macronutrient supplementation on BMI.** a. IPD analysis, b. Combined IPD and AD analysis in childhood and in adolescence. C. Combined IPD and AD analysis at >3 years. Data are mean and standard error, with adjusted mean difference (aMD) for treatment effect and 95% confidence intervals (CIs) adjusted for sex, gestational age and birthweight z-scores. The box size of point estimate is proportional to inverse variance. Heterogeneity of IPD analysis in childhood p=0.39, $tau^2$=0.03; in adolescence p=0.01, $tau^2$=0.51; at >3 years p=0.006, $tau^2$=0.04. Heterogeneity of combined IPD and AD analysis in childhood $tau^2$=0.02, in adolescence $tau^2$=0.25; at >3 years $tau^2$=0.02. IPD, individual participant data; AD, aggregated data.

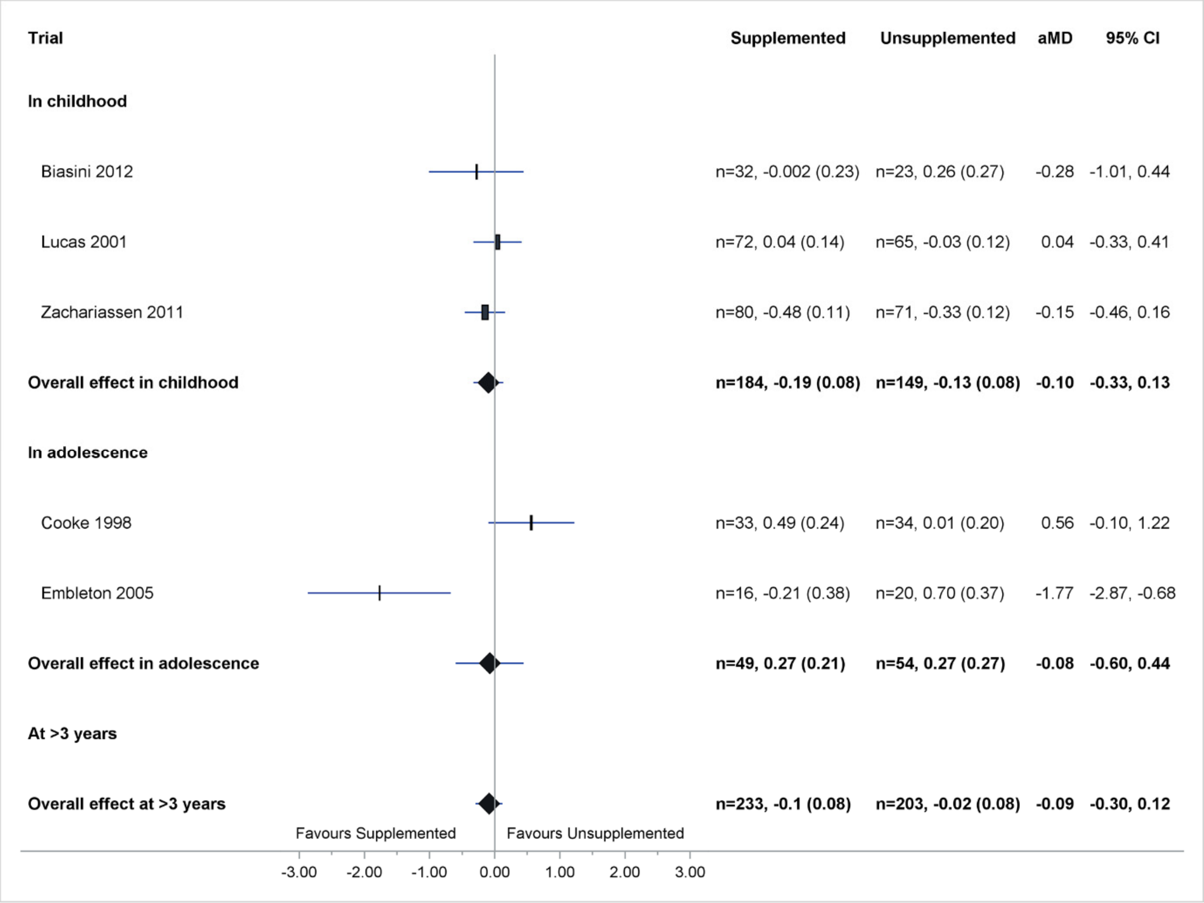


**Figure S14. Forest plot of effect of macronutrient supplementation on BMI z-scores (IPD analysis).** Data are mean and standard error, with adjusted mean difference (aMD) for treatment effect and 95% confidence intervals (CIs) adjusted for sex, gestational age and birthweight z-scores. The box size of point estimate is proportional to inverse variance. Heterogeneity in childhood p=0.64, tau$^2$=0.01; in adolescence p=0.005, tau$^2$=0.07; at >3 years p=0.04, tau$^2$=0.01.

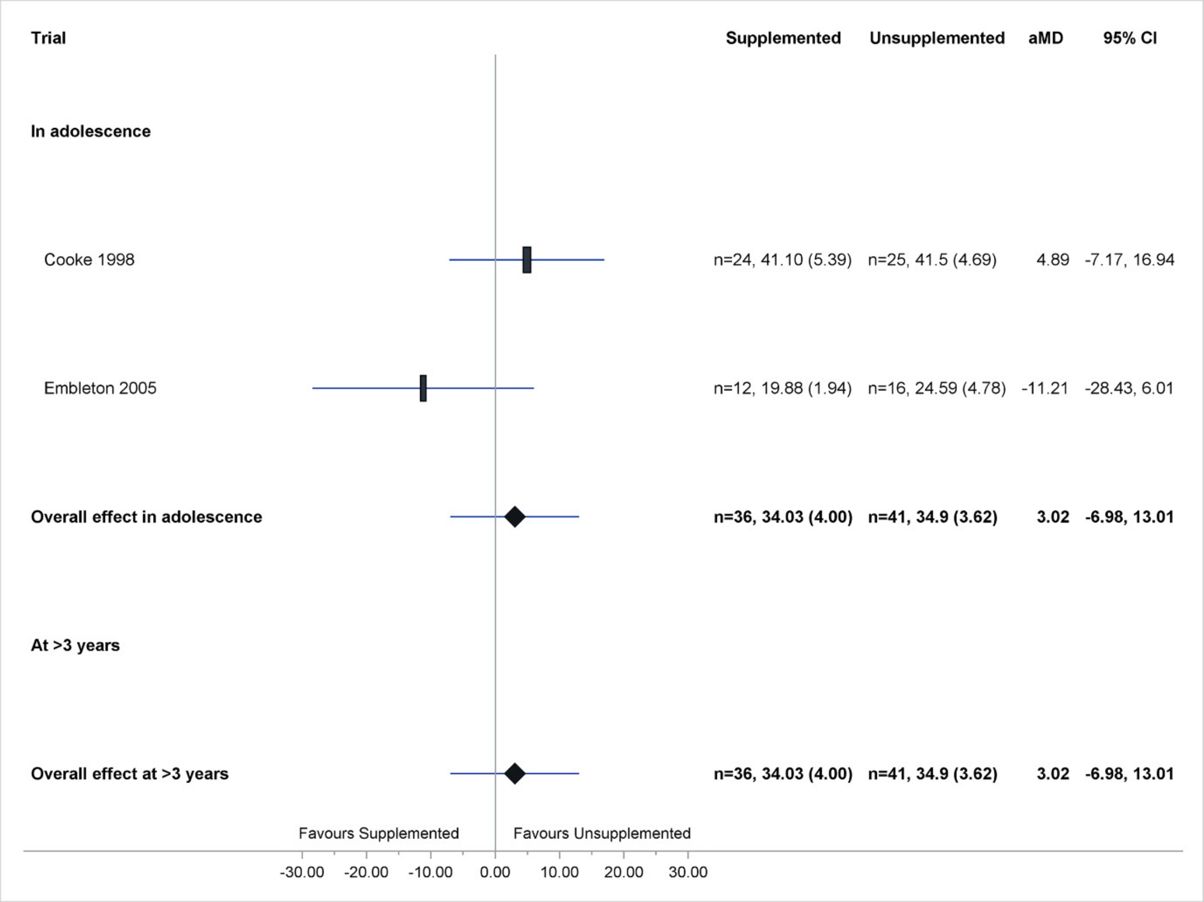


**Figure S15. Forest plot of effect of macronutrient supplementation on IGF-I concentration (IPD analysis).** Data are mean and standard error, with adjusted mean difference (aMD) for treatment effect and 95% confidence intervals (CIs) adjusted for sex, gestational age and birthweight z-scores. The box size of point estimate is proportional to inverse variance. Heterogeneity in adolescence p=0.80, tau$^2$=22.66.

a.

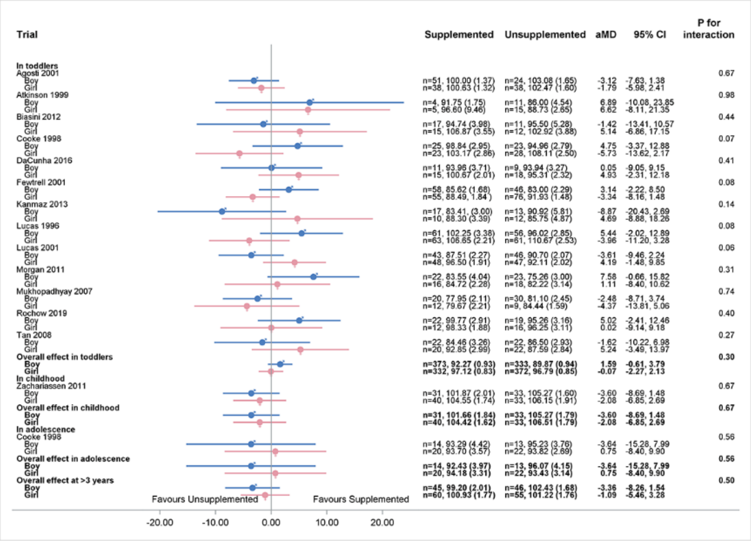


b.

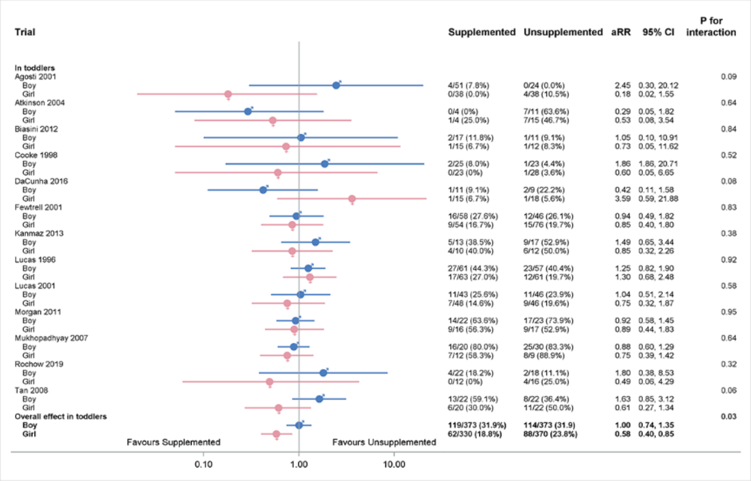


c.

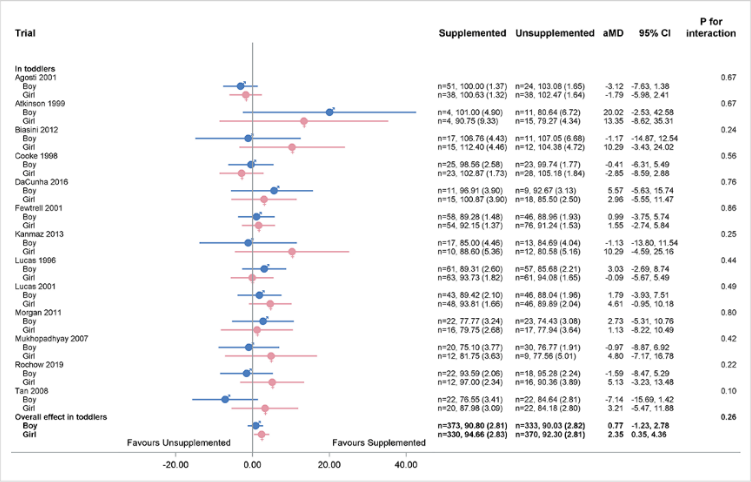


**Figure S16. IPD analysis of secondary developmental outcomes separated for boys and girls.** a. cognitive scores, b. motor impairment, c. motor scores. Data are mean and standard error, with adjusted mean difference (aMD) or data are numbers (percentages) with adjusted relative risk (aRR) and 95% confidence intervals (CIs) adjusted for gestational age and birthweight z-scores. Heterogeneity for a.cognitive scores, boys in toddlers p=0.34, tau$^2$=1.25; at >3 years p=0.91, tau$^2$=6.15; girls in toddlers p=0.27, tau$^2$=1.25; at >3 years p=0.53, tau$^2$=4.93. Heterogeneity for b. motor impairment, boys in toddlers p=0.98, tau$^2$=0.03; girls in toddlers p=0.95, tau$^2$=0.04. Heterogeneity for c. motor scores, boys in toddlers p=0.35, tau$^2$=0.03; girls in toddlers p=0.42, tau$^2$=0.04.

a.

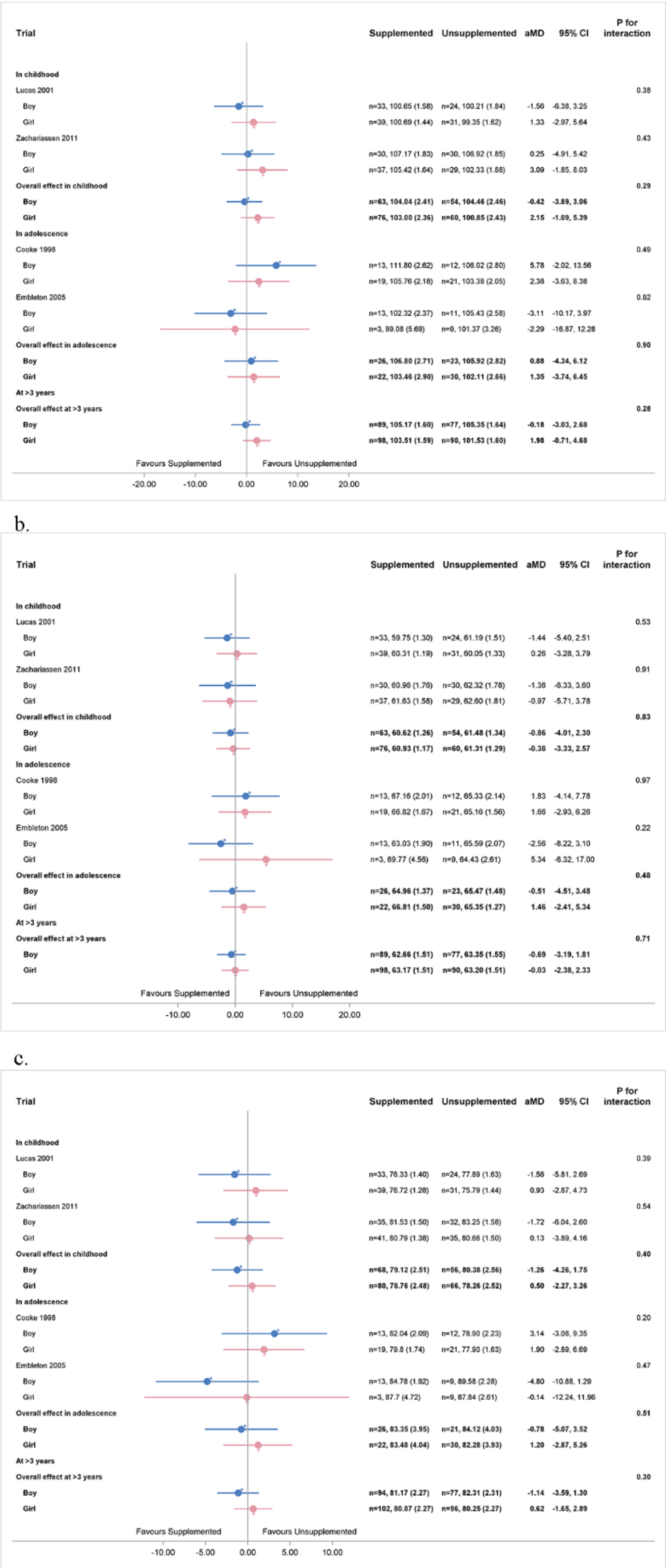


**Figure S17. IPD analysis of blood pressure separated for boys and girls.** a. SBP, b. DBP, c. MAP. Data are mean and standard error, with adjusted mean difference (aMD) and 95% confidence intervals (CIs) adjusted for gestational age and birthweight z-scores. Heterogeneity for a. SBP, boys in childhood p=0.63, $tau^2$=3.10; in adolescence p=0.19, $tau^2$=6.92; at >3 years p=0.64, $tau^2$=2.10. Girls in childhood p=0.54, $tau^2$=2.72; in adolescence p=0.19, $tau^2$=6.66; at >3 years p=0.74, $tau^2$=1.88. Heterogeneity for b. DBP, boys in childhood p=0.87, $tau^2$=2.56; in adolescence p= 0.25, $tau^2$=4.04; at >3 years p= 0.86, $tau^2$=1.61. Girls in childhood p=0.68, $tau^2$=2.25; in adolescence p=0.42, $tau^2$=3.80; at >3 years p=0.87, $tau^2$=1.44. Heterogeneity for c. MAP, boys in childhood p=0.87, $tau^2$=2.31; in adolescence p=0.11, $tau^2$=4.67; at >3 years p= 0.53, $tau^2$=1.56. Girls in childhood p=0.75, $tau^2$=1.99; in adolescence p=0.29, $tau^2$=4.20, at >3 years p= 0.94, $tau^2$=1.32.

a.

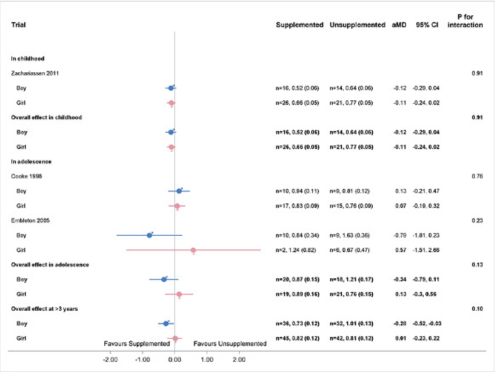

b.

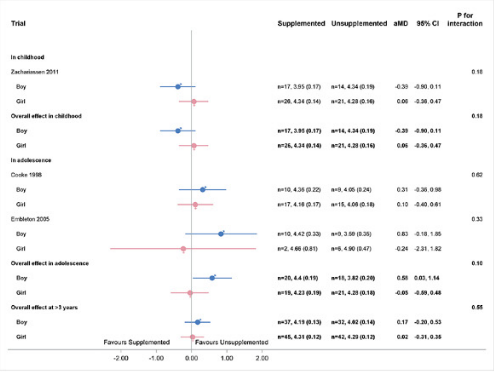

c.

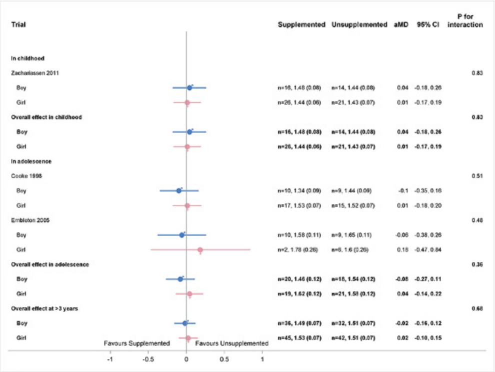

d.

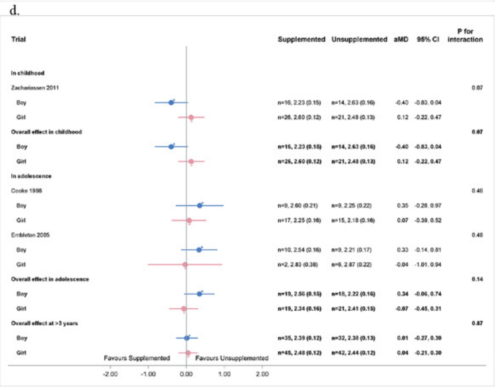

**Figure S18. IPD analysis of metabolic outcomes separated for boys and girls.** a. Triglyceride concentrations, b. Cholesterol concentrations, c. HDL concentrations, d. LDL concentrations. Data are mean and standard error, with adjusted mean difference (aMD) and 95% confidence intervals (CIs) adjusted for gestational age and birthweight z-scores. For triglyceride concentrations, heterogeneity for boys in adolescence p=0.08, $tau^2$=0.05; at >3 years p=0.07, $tau^2$=0.02. Heterogeneity for girls in adolescence p=0.39, $tau^2$=0.05; at >3 years p=0.13, $tau^2$=0.01. For cholesterol concentrations, heterogeneity for boys in adolescence p=0.49, $tau^2$=0.34; at >3 years p=0.34, $tau^2$=0.04. Heterogeneity for girls in adolescence p=0.04, $tau^2$=0.07; at >3 years p=0.01, $tau^2$=0.03. For HDL concentrations, heterogeneity for boys in adolescence p=0.77, $tau^2$=0.01; at >3 years p=0.89, $tau^2$=0.01. Heterogeneity for girls in adolescence p=0.93, $tau^2$=0.01; at >3 years p=0.98, $tau^2$=0.004. For LDL concentrations, heterogeneity for boys in adolescence p=0.98, $tau^2$=0.04; at >3 years p=0.11, $tau^2$=0.02. Heterogeneity for girls in adolescence p=0.59, $tau^2$=0.04; at >3 years p=0.84, $tau^2$=0.02.

a.

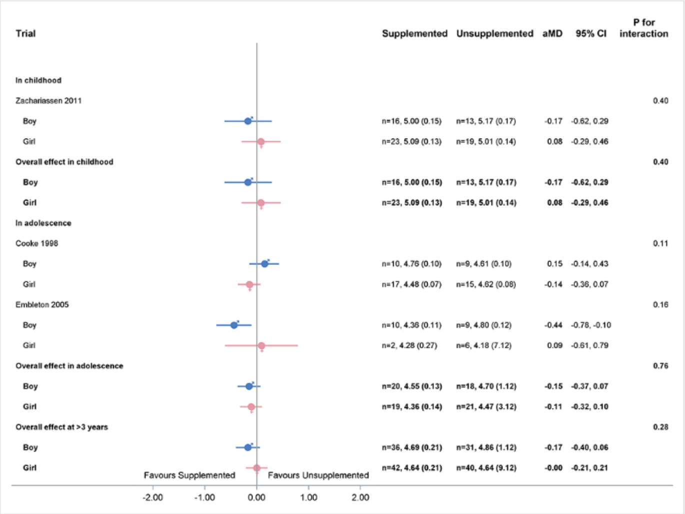


b.

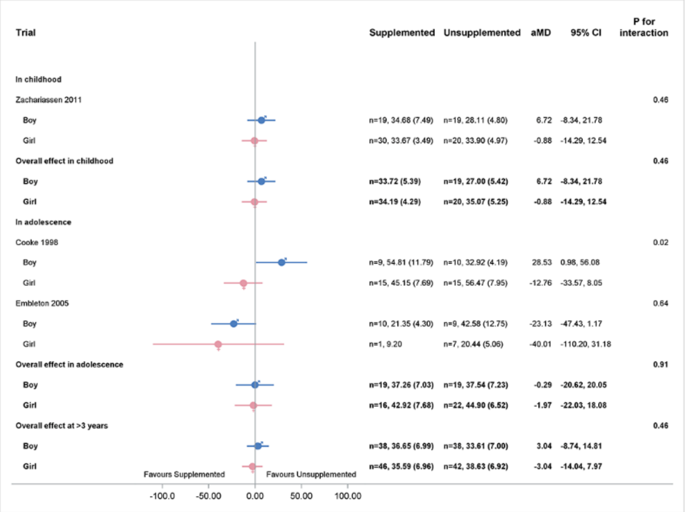


c.

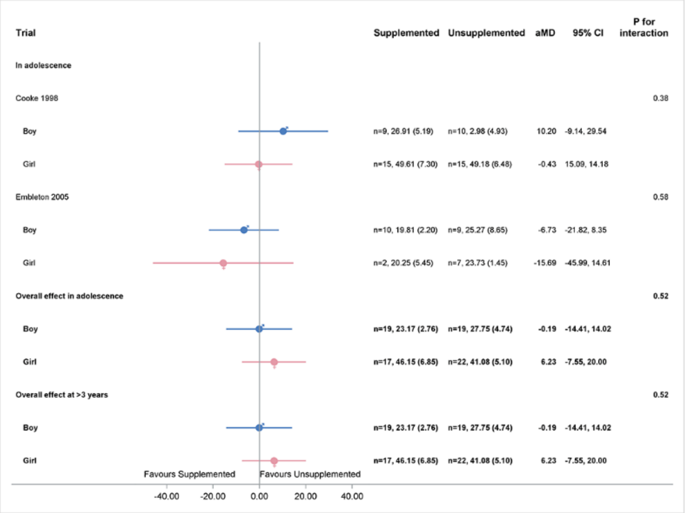


**Figure S19. IPD analysis of metabolic outcomes separated for boys and girls.** a. Blood glucose concentrations, b. Fasting insulin concentrations, c. IGF-I. Data are mean and standard error, with adjusted mean difference (aMD) and 95% confidence intervals (CIs) adjusted for gestational age and birthweight z-scores. For blood glucose concentrations, heterogeneity for boys in adolescence p=0.01, $tau^2$=0.01; at >3 years p=0.27, $tau^2$=0.11. Heterogeneity for girls in adolescence p=0.84, $tau^2$=0.01; at >3 years p=0.54, $tau^2$=0.11. For fasting insulin concentrations, heterogeneity for boys in adolescence p=0.03, $tau^2$=103.84; at >3 years p=0.06, $tau^2$=35.52. Heterogeneity for girls in adolescence p=0.41, $tau^2$=101.20; at >3 years p=0.52, $tau^2$=31.03. For IGF-I, heterogeneity for boys in adolescence p=0.85, $tau^2$=45.83; heterogeneity for girls in adolescence p=0.29, $tau^2$=43.69.

a.

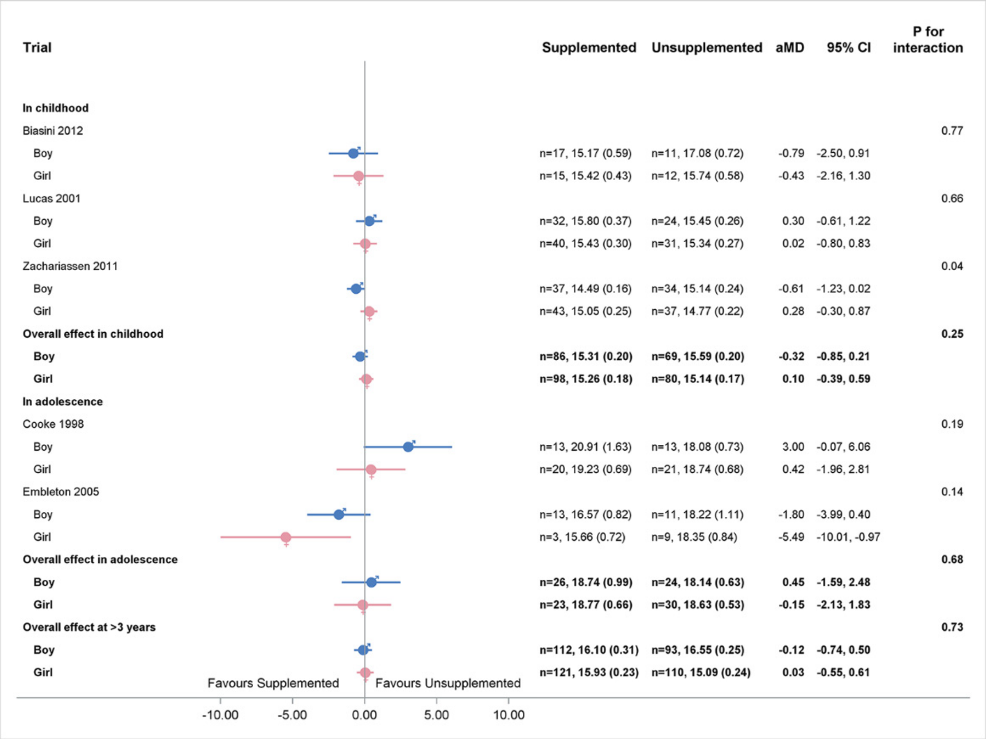


b.

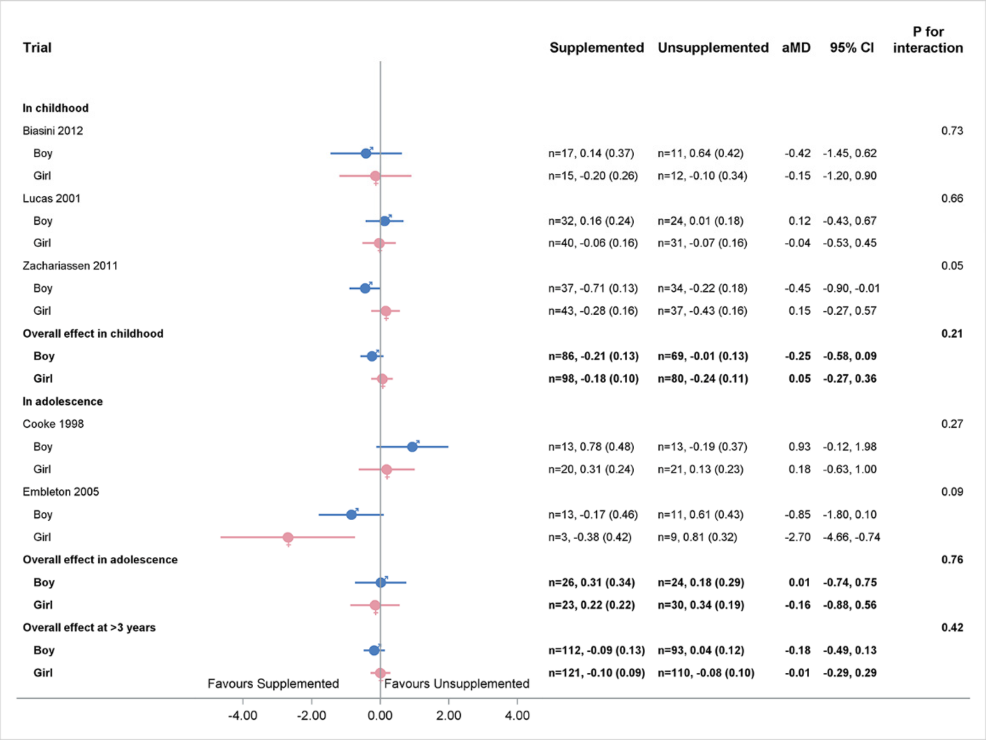


**Figure S20. IPD analysis of metabolic outcomes separated for boys and girls.** a. BMI, b. BMI z-scores. Data are mean and standard error, with adjusted mean difference (aMD) and 95% confidence intervals (CIs) adjusted for gestational age and birthweight z-scores. For BMI, heterogeneity for boys in childhood p= 0.17, tau²=0.07; in adolescence for boys p=0.08, tau²=1.04; at >3 years p=0.01, tau²=0.10. Heterogeneity for girls in childhood p=0.74, tau²=0.06; in adolescence p=0.06, p=1.00; at >3 years p=0·15, tau²=0.09. For BMI z-scores, heterogeneity for boys in childhood p= 0.28, tau²=0.03; in adolescence p=0.10, tau²=0.14; at >3 years p=0.09, tau²=0.03. Heterogeneity for girls in childhood p=0·82, tau²=0.02; in adolescence p=0.02, tau²=0.13; at >3 years p=0·24, tau²=0.02.

**Table S1. Risk of bias within studies**

| Study | Randomisation[1] | Concealment[2] | Performance[3] | Detection[4] | Attrition[5] | Reporting[6] |
|---|---|---|---|---|---|---|
| **Studies with IPD** | | | | | | |
| Agosti 2003 | Unclear | Unclear | Low | Low | High | Low |
| Atkinson 1999 | Low | Low | Low | Low | Low | Low |
| Biasini 2012 | High | High | High | Low | Low | Low |
| Cooke 1998 | Low | Low | Low | Low | Low | Low |
| Embleton 2005 | Low | Low | Low | Low | High | Low |
| da Cunha 2016 | Low | Low | High | Low | Low | Low |
| Fewtrell 2001 | Low | Low | Low | Low | Low | Low |
| Kanmaz 2013 | Low | Low | High | Low | Low | Low |
| Lucas 1996 | Low | Low | High | Low | Low | Low |
| Lucas 2001 | Low | Low | Low | Low | Low | Low |
| Morgan 2011 | Low | Low | Low | Low | Low | Low |
| Mukhopadhyay 2007 | Low | Low | Unclear | Unclear | Low | Low |
| Rochow 2019 | Low | Low | Low | Low | Low | Low |
| Tan 2008 | Low | Low | High | High | Low | Low |
| Zachariassen 2011 | Low | Low | High | High | Low | Low |
| **Studies with AD** | | | | | | |
| Amesz 2010 | Low | Low | Low | Unclear | High | Low |
| Bellagamba 2016 | Low | Unclear | Low | Low | Unclear | Low |
| Dorga 2017 | Low | Low | Low | Low | High | Low |
| Goldman 1969 | Unclear | Unclear | High | Low | High | Low |
| Jeon 2011 | Unclear | Unclear | Unclear | Unclear | High | Unclear |
| Lucas 1989 | Low | Low | High | Low | High | Low |
| Lucas 1990 | Low | Low | High | Low | High | Low |

| | | | | | | |
|---|---|---|---|---|---|---|
| O'Connor 2008 | Low | Low | Unclear | Unclear | High | Low |
| Roggero 2012 | Low | Unclear | Unclear | Low | Low | High |
| Svenningsen 1982 | Unclear | Unclear | Unclear | Unclear | Low | High |

[1]Random sequence generation. [2]Allocation concealment. [3]Blinding of participants and personnel. [4]Blinding of outcome assessment. [5]Incomplete outcome data. [6]Selective reporting.
We used IPD not the published data for studies with IPD, so the risk of reporting bias is low for all the studies with IPD.
IPD: individual participant data; AD: aggregated data.

**Table S2. Subgroup analyses of size for gestation of the infants**

| Outcome | Subgroup | No. of trials | No. of participants | aRR or aMD (95% CI) | P for overall effect | P for heterogeneity | Tau² | P for subgroup interaction |
|---|---|---|---|---|---|---|---|---|
| Cognitive impairment in toddlers | AGA | 13 trials | 990 | 1.05 (0.77, 1.41) | 0.76 | 0.91 | 0.02 | 0.21 |
| | SGA | 13 trials | 420 | 0.77 (0.54, 1.11) | 0.16 | 0.14 | 0.03 | |
| Cognitive impairment in childhood | AGA | 1 trial | 187 | 1.01 (0.62, 1.63) | 0.98 | N/A | N/A | 1.00 |
| | SGA | 1 trial | 20 | 1.00 (0.30, 3.31) | 0.99 | N/A | N/A | |
| Cognitive impairment in adolescence | AGA | 1 trial | 57 | 1.23 (0.54, 2.74) | 0.63 | N/A | N/A | 0.80 |
| | SGA | 1 trial | 12 | 1.64 (0.19, 14.31) | 0.65 | N/A | N/A | |
| Cognitive impairment at >3 years | AGA | 2 trials | 244 | 1.16 (0.50, 2.73) | 0.73 | 0.81 | 0.05 | 0.78 |
| | SGA | 2 trials | 32 | 1.02 (0.73, 1.44) | 0.91 | 0.59 | 0.29 | |
| Metabolic risk in childhood | AGA | 3 trials | 280 | 1.07 (0.78, 1.45) | 0.68 | 0.29 | 0.03 | 0.32 |
| | SGA | 3 trials | 54 | 0.72 (0.35, 1.48) | 0.37 | 0.34 | 0.14 | |
| Metabolic risk in adolescence | AGA | 2 trials | 84 | 0.89 (0.67, 1.18) | 0.42 | 0.09 | 0.27 | 0.77 |
| | SGA | 2 trials | 20 | 0.80 (0.41, 1.55) | 0.51 | 0.25 | 1.02 | |
| Metabolic risk at >3 years | AGA | 5 trials | 364 | 0.98 (0.78, 1.23) | 0.86 | 0.19 | 0.01 | 0.21 |
| | SGA | 5 trials | 74 | 0.68 (0.40, 1.16) | 0.15 | 0.33 | 0.07 | |
| Cognitive scores in toddlers | AGA | 13 trials | 990 | 0.27 (-1.58, 2.11) | 0.78 | 0.62 | 0.88 | 0.29 |
| | SGA | 13 trials | 420 | 2.11 (-0.76, 4.97) | 0.15 | 0.69 | 2.13 | |

| Outcome | Subgroup | No. of trials | No. of participants | aRR or aMD (95% CI) | P for overall effect | P for heterogeneity | Tau² | P for subgroup interaction |
|---|---|---|---|---|---|---|---|---|
| Cognitive scores in childhood | AGA | 1 trial | 187 | -2.01 (-5.66, 1.64) | 0.28 | N/A | N/A | 0.10 |
| | SGA | 1 trial | 20 | -12.92 (-25.36, -0.48) | 0.04 | N/A | N/A | |
| Cognitive scores in adolescence | AGA | 1 trial | 57 | -1.51 (-9.33, 6.31) | 0.70 | N/A | N/A | 0.63 |
| | SGA | 1 trial | 12 | 3.08 (-14.13, 20.28) | 0.72 | N/A | N/A | |
| Cognitive scores at >3 years | AGA | 2 trials | 244 | -1.68 (-5.16, 1.80) | 0.34 | 0.89 | 3.10 | 0.53 |
| | SGA | 2 trials | 32 | -5.02 (-14.89, 4.85) | 0.32 | 0.21 | 25 | |
| Motor impairment in toddlers | AGA | 13 trials | 987 | 0.91 (0.69, 1.23) | 0.56 | 0.97 | 0.02 | 0.09 |
| | SGA | 13 trials | 419 | 0.59 (0.38, 0.90) | 0.02 | 0.95 | 0.05 | |
| Motor scores in toddlers | AGA | 13 trials | 987 | 0.79 (-0.86, 2.45) | 0.35 | 0.34 | 0.74 | 0.06 |
| | SGA | 13 trials | 419 | 3.79 (1.17, 6.42) | 0.005 | 0.36 | 1.77 | |
| SBP in childhood (mmHg) | AGA | 2 trials | 219 | 1.55 (-1.01, 4.11) | 0.23 | 0.79 | 1.69 | 0.25 |
| | SGA | 2 trials | 34 | -2.58 (-9.11, 3.95) | 0.44 | 0.41 | 10.96 | |
| SBP in adolescence (mmHg) | AGA | 2 trials | 81 | 1.22 (-2.83, 5.27) | 0.55 | 0.36 | 4.17 | 0.93 |
| | SGA | 2 trials | 20 | 0.81 (-7.69, 9.31) | 0.85 | 0.03 | 18.32 | |
| SBP at >3 years (mmHg) | AGA | 4 trials | 300 | 1.50 (-0.63, 3.63) | 0.17 | 0.79 | 1.17 | 0.24 |
| | SGA | 4 trials | 54 | -1.76 (-6.82. 3.30) | 0.50 | 0.42 | 6.61 | |
| DBP in childhood (mmHg) | AGA | 2 trials | 219 | -0.37 (-2.69, 1.95) | 0.75 | 0.41 | 1.39 | 0.59 |
| | SGA | 2 trials | 34 | -2.11 (-8.02, 3.80) | 0.48 | 0.27 | 9.00 | |

| Outcome | Subgroup | No. of trials | No. of participants | aRR or aMD (95% CI) | P for overall effect | P for heterogeneity | Tau² | P for subgroup interaction |
|---|---|---|---|---|---|---|---|---|
| DBP in adolescence (mmHg) | AGA | 2 trials | 81 | 1.71 (-1.36, 4.78) | 0.27 | 0.89 | 2.40 | 0.11 |
| | SGA | 2 trials | 20 | -4.08 (-10.50, 2.33) | 0.21 | 0.01 | 10.43 | |
| DBP at >3 years (mmHg) | AGA | 4 trials | 300 | 0.15 (-1.70, 2.01) | 0.87 | 0.66 | 0.88 | 0.18 |
| | SGA | 4 trials | 54 | -3.13 (-7.54, 1.28) | 0.16 | 0.22 | 5.02 | |
| MAP in childhood (mmHg) | AGA | 2 trials | 235 | -0.08 (-2.26, 2.11) | 0.95 | 0.46 | 1.23 | 0.56 |
| | SGA | 2 trials | 35 | -1.87 (-7.58, 3.83) | 0.52 | 0.42 | 8.41 | |
| MAP in adolescence (mmHg) | AGA | 2 trials | 79 | -0.07 (-3.36, 3.22) | 0.97 | 0.07 | 2.76 | 0.65 |
| | SGA | 2 trials | 20 | 1.67 (-5.13, 8.46) | 0.63 | 0.48 | 11.70 | |
| MAP at >3 years (mmHg) | AGA | 4 trials | 314 | -0.09 (-1.90, 1.72) | 0.92 | 0.28 | 0.85 | 0.74 |
| | SGA | 4 trials | 55 | -0.89 (-5.23, 3.46) | 0.69 | 0.68 | 4.88 | |
| Triglyceride concentrations in childhood (mmol/L) | AGA | 1 trial | 68 | -0.11 (-0.22, 0.003) | 0.06 | N/A | N/A | 0.53 |
| | SGA | 1 trial | 9 | -0.22 (-0.56, 0.12) | 0.19 | N/A | N/A | |
| Triglyceride concentrations in adolescence (mmol/L) | AGA | 2 trials | 21 | 0.02 (-0.31, 0.35) | 0.89 | 0.64 | 0.03 | 0.10 |
| | SGA | 2 trials | 6 | -0.70 (-1.50, 0.10) | 0.09 | 0.20 | 0.16 | |
| Triglyceride concentrations at >3 years (mmol/L) | AGA | 3 trials | 133 | -0.03 (-0.21, 0.14) | 0.71 | 0.46 | 0.01 | 0.008 |
| | SGA | 3 trials | 22 | -0.66 (-1.09, -0.23) | 0.03 | 0.26 | 0.05 | |

| Outcome | Subgroup | No. of trials | No. of participants | aRR or aMD (95% CI) | P for overall effect | P for heterogeneity | Tau² | P for subgroup interaction |
|---|---|---|---|---|---|---|---|---|
| Cholesterol concentrations in childhood (mmol/L) | AGA | 1 trial | 69 | -0.19 (-0.53, 0.15) | 0.27 | N/A | N/A | 0.30 |
| | SGA | 1 trial | 9 | 0.39 (-0.65, 1.42) | 0.46 | N/A | N/A | |
| Cholesterol concentrations in adolescence (mmol/L) | AGA | 2 trials | 65 | 0.17 (-0.24, 0.57) | 0.42 | 0.21 | 0.04 | 0.38 |
| | SGA | 2 trials | 13 | 0.63 (-0.35, 1.61) | 0.20 | 0.40 | 0.05 | |
| Cholesterol concentrations at >3 years (mmol/L) | AGA | 3 trials | 134 | -0.02 (-0.28, 0.24) | 0.88 | 0.19 | 0.05 | 0.03 |
| | SGA | 3 trials | 22 | 0.74 (0.09, 1.39) | 0.03 | 0.18 | 0.11 | |
| HDL concentrations in childhood (mmol/L) | AGA | 1 trial | 68 | 0.003 (-0.15, 0.15) | 0.97 | N/A | N/A | 0.37 |
| | SGA | 1 trial | 9 | 0.21 (-0.23, 0.65) | 0.34 | N/A | N/A | |
| HDL concentrations in adolescence (mmol/L) | AGA | 2 trials | 65 | -0.04 (-0.18, 0.10) | 0.58 | 0.41 | 0.01 | 0.43 |
| | SGA | 2 trials | 13 | 0.11 (-0.24, 0.45) | 0.53 | 0.02 | 0.03 | |
| HDL concentrations at >3 years (mmol/L) | AGA | 3 trials | 133 | -0.02 (-0.12, 0.08) | 0.69 | 0.67 | 0.003 | 0.21 |
| | SGA | 3 trials | 22 | 0.15 (-0.10, 0.40) | 0.24 | 0.05 | 0.02 | |
| LDL concentrations in childhood (mmol/L) | AGA | 1 trial | 68 | -0.13 (-0.43, 016) | 0.38 | N/A | N/A | 0.39 |
| | SGA | 1 trial | 9 | 0.27 (-0.61, 1.15) | 0.54 | N/A | N/A | |
| LDL concentrations in adolescence (mmol/L) | AGA | 2 trials | 64 | 0.17 (-0.12, 0.47) | 0.25 | 0.49 | 0.02 | 0.40 |
| | SGA | 2 trials | 13 | -0.16 (-0.87, 0.56) | 0.66 | 0.37 | 0.13 | |

| Outcome | Subgroup | No. of trials | No. of participants | aRR or aMD (95% CI) | P for overall effect | P for heterogeneity | Tau² | P for subgroup interaction |
|---|---|---|---|---|---|---|---|---|
| LDL concentrations at >3 years (mmol/L) | AGA | 3 trials | 132 | 0.02 (-0.19, 0.23) | 0.84 | 0.27 | 0.01 | 0.72 |
| | SGA | 3 trials | 22 | 0.12 (-0.39, 0.63) | 0.64 | 0.22 | 0.07 | |
| Blood glucose concentrations in childhood (mmol/L) | AGA | 1 trial | 62 | 0.03 (-0.28, 0.33) | 0.86 | N/A | N/A | 0.36 |
| | SGA | 1 trial | 9 | -0.40 (-1.28, 0.48) | 0.36 | N/A | N/A | |
| Blood glucose concentrations in adolescence (mmol/L) | AGA | 2 trials | 65 | -0.13 (-0.29, 0.04) | 0.13 | 0.38 | 0.01 | 0.77 |
| | SGA | 2 trials | 13 | -0.19 (-0.59, 0.21) | 0.34 | 0.01 | 0.04 | |
| Blood glucose concentrations at >3 years (mmol/L) | AGA | 3 trials | 127 | -0.05 (-0.21, 0.12) | 0.60 | 0.60 | 0.01 | 0.29 |
| | SGA | 3 trials | 22 | -0.28 (-0.69, 0.13) | 0.17 | 0.20 | 0.04 | |
| Fasting insulin in childhood (pmol/L) | AGA | 1 trial | 81 | 3.97 (-6.68, 14.62) | 0.46 | N/A | N/A | 0.40 |
| | SGA | 1 trial | 7 | -14.20 (-54.96, 26.55) | 0.49 | N/A | N/A | |
| Fasting insulin in adolescence (pmol/L) | AGA | 2 trials | 62 | -8.91 (-23.30, 5.47) | 0.22 | 0.31 | 51.98 | 0.04 |
| | SGA | 2 trials | 14 | 29.67 (-3.65, 62.98) | 0.08 | 0.30 | 27.89 | |
| Fasting insulin at >3 years (pmol/L) | AGA | 3 trials | 143 | -1.56 (-10.13, 7.00) | 0.72 | 0.18 | 27.25 | 0.25 |
| | SGA | 3 trials | 21 | 12.86 (-9.95, 35.66) | 0.27 | 0.53 | 133.17 | |
| IGF-I in adolescence (nmol/L) | AGA | 2 trials | 63 | -2.45 (-12.95, 8.05) | 0.64 | 0.83 | 27.67 | 0.15 |
| | SGA | 2 trials | 14 | 17.32 (-7.26, 41.90) | 0.16 | 0.19 | 151.78 | |

| Outcome | Subgroup | No. of trials | No. of participants | aRR or aMD (95% CI) | P for overall effect | P for heterogeneity | Tau$^2$ | P for subgroup interaction |
|---|---|---|---|---|---|---|---|---|
| BMI in childhood (kg/m$^2$) | AGA | 3 trials | 280 | 0.02 (-0.38, 0.41) | 0.93 | 0.63 | 0.04 | 0.21 |
| | SGA | 3 trials | 53 | -0.63 (-1.55, 0.29) | 0.18 | 0.17 | 0.25 | |
| BMI in adolescence (kg/m$^2$) | AGA | 2 trials | 83 | 0.04 (-1.54, 1.61) | 0.96 | 0.03 | 0.64 | 0.68 |
| | SGA | 2 trials | 20 | 0.82 (-2.54, 4.18) | 0.63 | 0.01 | 2.89 | |
| BMI at >3 years (kg/m$^2$) | AGA | 5 trials | 363 | 0.01 (-0.46, 0.48) | 0.97 | 0.01 | 0.06 | 0.79 |
| | SGA | 5 trials | 73 | -0.15 (-1.20, 0.90) | 0.78 | 0.01 | 0.28 | |
| BMI z-score in childhood | AGA | 3 trials | 280 | -0.03 (-0.28, 0.23) | 0.85 | 0.72 | 0.02 | 0.29 |
| | SGA | 3 trials | 53 | -0.37 (-0.96, 0.22) | 0.21 | 0.25 | 0.09 | |
| BMI z-score in adolescence | AGA | 2 trials | 83 | -0.05 (-0.63, 0.53) | 0.87 | 0.02 | 0.08 | 1.00 |
| | SGA | 2 trials | 20 | -0.05 (-1.29, 1.18) | 0.93 | 0.004 | 0.38 | |
| BMI z-score at >3 years | AGA | 5 trials | 363 | -0.04 (-0.28, 0.19) | 0.73 | 0.06 | 0.01 | 0.55 |
| | SGA | 5 trials | 73 | -0.22 (-0.74, 0.31) | 0.42 | 0.01 | 0.07 | |

Abbreviation: AGA: appropriate for gestational age; SGA: small for gestational age; SBP: systolic blood pressure; DBP: diastolic blood pressure; MAP: mean arterial pressure; HDL: high-density lipoproteins; LDL: low-density lipoproteins; BMI: body mass index; aRR: adjusted relative risk; aMD: adjusted mean difference; N/A: not applicable

Relative risk and mean difference were adjusted for sex and gestational age.

**Table S3. Subgroup analyses of size of infant at birth**

| Outcome | Subgroup | No. of trials | No. of participants | aRR or aMD (95% CI) | P for overall effect | P for heterogeneity | Tau² | P for subgroup interaction |
|---|---|---|---|---|---|---|---|---|
| Cognitive impairment in toddlers | ≤1 kg | 12 trials | 335 | 0.85 (0.63, 1.14) | 0.28 | 0.99 | 0.02 | 0.47 |
| | >1 kg | 13 trials | 1075 | 0.92 (0.76, 1.11) | 0.39 | 0.99 | 0.01 | |
| Cognitive impairment in childhood | ≤1 kg | 1 trial | 41 | 0.96 (0.42, 2.19) | 0.93 | N/A | N/A | 0.89 |
| | >1 kg | 1 trial | 166 | 1.03 (0.68, 1.55) | 0.90 | N/A | N/A | |
| Cognitive impairment in adolescence | ≤1 kg | 1 trial | 9 | 1.17 (0.08, 17.86) | 0.89 | N/A | N/A | 0.74 |
| | >1 kg | 1 trial | 60 | 1.36 (0.66, 2.79) | 0.40 | N/A | N/A | |
| Cognitive impairment at >3 years | ≤1 kg | 2 trials | 50 | 0.98 (0.48, 2.02) | 0.97 | 0.84 | 0.13 | 0.86 |
| | >1 kg | 2 trials | 226 | 1.06 (0.75, 1.52) | 0.73 | 0.72 | 0.03 | |
| Metabolic risk in childhood | ≤1 kg | 3 trials | 77 | 1.43 (0.66, 3.08) | 0.36 | 0.21 | 0.14 | 0.06 |
| | >1 kg | 3 trials | 257 | 0.93 (0.69, 1.26) | 0.63 | 0.38 | 0.02 | |
| Metabolic risk in adolescence | ≤1 kg | 2 trials | 10 | 1.32 (0.39, 4.50) | 0.61 | 0.84 | 0.27 | 0.39 |
| | >1 kg | 2 trials | 94 | 0.85 (0.65, 1.11) | 0.24 | 0.39 | 0.02 | |
| Metabolic risk at >3 years | ≤1 kg | 5 trials | 87 | 1.43 (0.74, 2.79) | 0.29 | 0.36 | 0.11 | 0.09 |
| | >1 kg | 5 trials | 351 | 0.87 (0.70, 1.09) | 0.22 | 0.62 | 0.01 | |
| Cognitive scores in toddlers | ≤1 kg | 12 trials | 335 | 1.56 (-1.76, 4.89) | 0.35 | 0.55 | 2.86 | 0.49 |
| | >1 kg | 13 trials | 1075 | 0.41 (-1.37, 2.19) | 0.65 | 0.96 | 0.83 | |
| | ≤1 kg | 1 trial | 41 | -6.89 (-18.01, 4.24) | 0.21 | N/A | N/A | 0.29 |

| Outcome | Subgroup | No. of trials | No. of participants | aRR or aMD (95% CI) | P for overall effect | P for heterogeneity | Tau² | P for subgroup interaction |
|---|---|---|---|---|---|---|---|---|
| Cognitive scores in childhood | >1 kg | 1 trial | 166 | -2.19 (-5.72. 1.34) | 0.22 | N/A | N/A | |
| Cognitive scores in adolescence | ≤1 kg | 1 trial | 9 | 10.72 (-17.08, 38.51) | 0.38 | N/A | N/A | 0.19 |
| | >1 kg | 1 trial | 60 | -2.54 (-10.04, 4.37) | 0.50 | N/A | N/A | |
| Cognitive scores at >3 years | ≤1 kg | 2 trials | 50 | -2.36 (-12.23, 7.51) | 0.63 | 0.12 | 23.52 | 0.84 |
| | >1 kg | 2 trials | 226 | -2.15 (-5.59, 1.29) | 0.22 | 0.94 | 3.03 | |
| Motor impairment in toddlers | ≤1 kg | 12 trials | 334 | 0.92 (0.69, 1.23) | 0.58 | 0.99 | 0.06 | 0.85 |
| | >1 kg | 13 trials | 1072 | 0.82 (0.60, 1.12) | 0.21 | 0.97 | 0.03 | |
| Motor scores in toddlers | ≤1 kg | 12 trials | 334 | 0.74 (-2.20, 3.69) | 0.62 | 0.69 | 2.82 | 0.61 |
| | >1 kg | 13 trials | 1072 | 1.61 (-0.04, 3.25) | 0.06 | 0.48 | 0.66 | |
| SBP in childhood (mmHg) | ≤1 kg | 2 trials | 43 | -1.58 (-8.34, 5.18) | 0.64 | 0.44 | 11.16 | 0.38 |
| | >1 kg | 2 trials | 210 | 1.34 (-1.22, 3.91) | 0.30 | 0.78 | 1.69 | |
| SBP in adolescence (mmHg) | ≤1 kg | 2 trials | 9 | -13.42 (-27.54, 0.71) | 0.06 | 0.12 | 30.25 | 0.07 |
| | >1 kg | 2 trials | 92 | 2.19 (-1.60, 5.97) | 0.25 | 0.18 | 3.61 | |
| SBP at >3 years (mmHg) | ≤1 kg | 4 trials | 52 | -3.42 (-9.30, 2.47) | 0.25 | 0.70 | 8.53 | 0.12 |
| | >1 kg | 4 trials | 302 | 1.61 (-0.49, 3.71) | 0.13 | 0.60 | 1.12 | |
| DBP in childhood (mmHg) | ≤1 kg | 2 trials | 43 | -1.02 (-7.73, 5.70) | 0.76 | 0.93 | 11.02 | 0.90 |
| | >1 kg | 2 trials | 210 | -0.72 (-2.96, 1.53) | 0.53 | 0.81 | 1.30 | |

| Outcome | Subgroup | No. of trials | No. of participants | aRR or aMD (95% CI) | P for overall effect | P for heterogeneity | Tau² | P for subgroup interaction |
|---|---|---|---|---|---|---|---|---|
| DBP in adolescence (mmHg) | ≤1 kg | 2 trials | 9 | -0.11 (-5.91, 5.68) | 0.96 | 0.63 | 5.06 | 0.06 |
| | >1 kg | 2 trials | 92 | 1.27 (-1.56, 4.11) | 0.37 | 0.008 | 2.05 | |
| DBP at >3 years (mmHg) | ≤1 kg | 4 trials | 52 | -2.48 (-8.22, 3.25) | 0.39 | 0.94 | 8.12 | 0.49 |
| | >1 kg | 4 trials | 302 | -0.18 (-1.95, 1.59) | 0.84 | 0.75 | 0.81 | |
| MAP in childhood (mmHg) | ≤1 kg | 2 trials | 46 | -2.68 (-8.60, 3.24) | 0.37 | 0.85 | 8.58 | 0.33 |
| | >1 kg | 2 trials | 224 | 0.05 (-2.12, 2.22) | 0.97 | 0.93 | 1.21 | |
| MAP in adolescence (mmHg) | ≤1 kg | 2 trials | 9 | -0.95 (-10.46, 8.56) | 0.81 | 0.15 | 13.69 | 0.94 |
| | >1 kg | 2 trials | 90 | 0.24 (-2.91, 3.39) | 0.88 | 0.05 | 2.53 | |
| MAP at >3 years (mmHg) | ≤1 kg | 4 trials | 55 | -1.99 (-7.18, 3.21) | 0.45 | 0.96 | 6.66 | 0.36 |
| | >1 kg | 4 trials | 314 | 0.06 (-1.72, 1.83) | 0.95 | 0.31 | 0.81 | |
| Triglyceride concentrations in childhood (mmol/L) | ≤1 kg | 1 trial | 13 | -0.03 (-0.28, 0.22) | 0.78 | N/A | N/A | 0.31 |
| | >1 kg | 1 trial | 64 | -0.14 (-0.26, -0.02) | 0.03 | N/A | N/A | |
| Triglyceride concentrations in adolescence (mmol/L) | ≤1 kg | 2 trials | 9 | -0.22 (-0.56, 0.13) | 0.17 | 0.35 | 0.02 | 0.87 |
| | >1 kg | 2 trials | 69 | -0.16 (-0.51, 0.19) | 0.38 | 0.10 | 0.03 | |
| Triglyceride concentrations at >3 years (mmol/L) | ≤1 kg | 3 trials | 22 | -0.13 (-0.33, 0.07) | 0.18 | 0.76 | 0.01 | 0.78 |
| | >1 kg | 3 trials | 133 | -0.12 (-0.31, 0.07) | 0.22 | 0.17 | 0.01 | |
| | ≤1 kg | 1 trial | 14 | 0.34 (-0.20, 0.87) | 0.20 | N/A | N/A | 0.25 |

| Outcome | Subgroup | No. of trials | No. of participants | aRR or aMD (95% CI) | P for overall effect | P for heterogeneity | Tau² | P for subgroup interaction |
|---|---|---|---|---|---|---|---|---|
| Cholesterol concentrations in childhood (mmol/L) | >1 kg | 1 trial | 64 | -0.23 (-0.61, 0.15) | 0.24 | N/A | N/A | |
| Cholesterol concentrations in adolescence (mmol/L) | ≤1 kg | 2 trials | 9 | -0.23 (-1.07, 0.61) | 0.52 | 0.20 | 0.11 | 0.63 |
| | >1 kg | 2 trials | 69 | 0.34 (-0.09, 0.78) | 0.12 | 0.67 | 0.05 | |
| Cholesterol concentrations at >3 years (mmol/L) | ≤1 kg | 3 trials | 23 | 0.18 (-0.22, 0.58) | 0.36 | 0.12 | 0.04 | 0.74 |
| | >1 kg | 3 trials | 133 | 0.08 (-0.21, 0.36) | 0.60 | 0.11 | 0.02 | |
| HDL concentrations in childhood (mmol/L) | ≤1 kg | 1 trial | 13 | 0.20 (-0.13, 0.52) | 0.20 | N/A | N/A | 0.20 |
| | >1 kg | 1 trial | 64 | -0.01 (-0.17, 0.15) | 0.92 | N/A | N/A | |
| HDL concentrations in adolescence (mmol/L) | ≤1 kg | 2 trials | 9 | -0.04 (-0.82, 0.73) | 0.89 | 0.15 | 0.09 | 0.30 |
| | >1 kg | 2 trials | 69 | -0.05 (-0.18, 0.08) | 0.45 | 0.46 | 0.004 | |
| HDL concentrations at >3 years (mmol/L) | ≤1 kg | 3 trials | 22 | 0.23 (-0.08, 0.55) | 0.14 | 0.27 | 0.02 | 0.06 |
| | >1 kg | 3 trials | 133 | -0.04 (-0.14, 0.06) | 0.47 | 0.72 | 0.003 | |
| LDL concentrations in childhood (mmol/L) | ≤1 kg | 1 trial | 13 | 0.18 (-0.44, 0.81) | 0.53 | N/A | N/A | 0.50 |
| | >1 kg | 1 trial | 64 | -0.15 (-0.46, 0.17) | 0.36 | N/A | N/A | |
| LDL concentrations in adolescence (mmol/L) | ≤1 kg | 2 trials | 8 | -0.23 (-0.78, 0.32) | 0.31 | 0.37 | 0.04 | 0.38 |
| | >1 kg | 2 trials | 69 | 0.21 (-0.11, 0.52) | 0.19 | 0.71 | 0.02 | |
| | ≤1 kg | 3 trials | 21 | 0.04 (-0.18, 0.26) | 0.73 | 0.24 | 0.03 | 0.88 |

| Outcome | Subgroup | No. of trials | No. of participants | aRR or aMD (95% CI) | P for overall effect | P for heterogeneity | Tau² | P for subgroup interaction |
|---|---|---|---|---|---|---|---|---|
| LDL concentrations at >3 years (mmol/L) | >1 kg | 3 trials | 133 | 0.05 (-0.32, 0.42) | 0.76 | 0.28 | 0.01 | |
| Blood glucose concentrations in childhood (mmol/L) | ≤1 kg | 1 trial | 13 | 0.00 (-0.81, 0.81) | 1.00 | N/A | N/A | 0.96 |
| | >1 kg | 1 trial | 58 | -0.02 (-0.34, 0.30) | 0.88 | N/A | N/A | |
| Blood glucose concentrations in adolescence (mmol/L) | ≤1 kg | 2 trials | 9 | -0.30 (-0.91, 0.31) | 0.26 | 0.08 | 0.06 | 0.17 |
| | >1 kg | 2 trials | 69 | -0.12 (-0.28, 0.04) | 0.14 | 0.22 | 0.01 | |
| Blood glucose concentrations at >3 years (mmol/L) | ≤1 kg | 3 trials | 22 | -0.15 (-0.65, 0.35) | 0.54 | 0.69 | 0.06 | 0.57 |
| | >1 kg | 3 trials | 127 | -0.07 (-0.23, 0.10) | 0.44 | 0.64 | 0.01 | |
| BMI in childhood (kg/m²) | ≤1 kg | 3 trials | 76 | 0.47 (-0.37, 1.31) | 0.27 | 0.13 | 0.18 | 0.12 |
| | >1 kg | 3 trials | 257 | -0.25 (-0.66, 0.15) | 0.22 | 0.26 | 0.04 | |
| BMI in adolescence (kg/m²) | ≤1 kg | 2 trials | 10 | -3.05 (-6.59, 0.50) | 0.08 | 0.20 | 2.07 | 0.22 |
| | >1 kg | 2 trials | 93 | 0.57 (-0.95, 2.08) | 0.46 | 0.04 | 0.58 | |
| BMI at >3 years (kg/m²) | ≤1 kg | 5 trials | 86 | 0.13 (-0.70, 0.96) | 0.75 | 0.08 | 0.02 | 0.76 |
| | >1 kg | 5 trials | 350 | -0.05 (-0.54, 0.45) | 0.86 | 0.01 | 0.06 | |
| BMI z-score in childhood | ≤1 kg | 3 trials | 76 | 0.33 (-0.20, 0.86) | 0.22 | 0.11 | 0.08 | 0.07 |
| | >1 kg | 3 trials | 257 | -0.21 (-0.46, 0.05) | 0.12 | 0.45 | 0.02 | |
| | ≤1 kg | 2 trials | 10 | -1.19 (-3.01, 0.63) | 0.16 | 0.38 | 0.56 | 0.13 |

| Outcome | Subgroup | No. of trials | No. of participants | aRR or aMD (95% CI) | P for overall effect | P for heterogeneity | Tau² | P for subgroup interaction |
|---|---|---|---|---|---|---|---|---|
| BMI z-score in adolescence | >1 kg | 2 trials | 93 | 0.13 (-0.41, 0.68) | 0.63 | 0.03 | 0.07 | |
| BMI z-score at >3 years | ≤1 kg | 5 trials | 86 | 0.16 (-0.35, 0.66) | 0.54 | 0.11 | 0.06 | 0.36 |
| | >1 kg | 5 trials | 350 | -0.12 (-0.36, 0.12) | 0.33 | 0.04 | 0.01 | |
| Fasting insulin in childhood (pmol/L) | ≤1 kg | 1 trial | 16 | 3.49 (-28.34, 35.31) | 0.82 | N/A | N/A | 0.67 |
| | >1 kg | 1 trial | 72 | 0.93 (-10.03, 11.89) | 0.87 | N/A | N/A | |
| Fasting insulin in adolescence (pmol/L) | ≤1 kg | 2 trials | 9 | -3.78 (-18.64, 11.09) | 0.61 | 0.41 | 190.72 | 0.80 |
| | >1 kg | 2 trials | 67 | -3.97 (-39.47, 31.52) | 0.79 | 0.49 | 55.35 | |
| Fasting insulin at >3 years (pmol/L) | ≤1 kg | 3 trials | 25 | -0.93 (-9.83, 7.96) | 0.84 | 0.58 | 20.25 | 0.61 |
| | >1 kg | 3 trials | 139 | -0.03 (-21.47, 21.41) | 0.99 | 0.99 | 10.68 | |
| IGF-I in adolescence (nmol/L) | ≤1 kg | 2 trials | 9 | -14.31 (-47.77, 19.15) | 0.32 | 0.60 | 169.52 | 0.49 |
| | >1 kg | 2 trials | 68 | 0.53 (-10.01, 11.07) | 0.92 | 0.89 | 27.88 | |

Abbreviation: SBP: systolic blood pressure; DBP diastolic blood pressure; MAP: mean arterial pressure; HDL: high-density lipoproteins; LDL: low-density lipoproteins; BMI: body mass index; aRR: adjusted relative risk; aMD: adjusted mean difference; N/A: not applicable
Relative risk and mean difference were adjusted for sex.

**Table S4. Subgroup analyses of gestational age of infant at birth**

| Outcome | Subgroup | No. of trials | No. of participants | aRR or aMD (95% CI) | P for overall effect | P for heterogeneity | Tau² | P for subgroup interaction |
|---|---|---|---|---|---|---|---|---|
| Cognitive impairment in toddlers | ≤28 weeks | 12 trials | 469 | 0.94 (0.71, 1.23) | 0.64 | 0.93 | 0.46 | 0.95 |
| | 29 to 32 weeks | 10 trials | 544 | 0.81 (0.61, 1.08) | 0.15 | 0.99 | 0.02 | |
| | 33 to 36 weeks | 7 trials | 157 | 0.81 (0.48, 1.35) | 0.42 | 0.91 | 0.07 | |
| Cognitive impairment in childhood | ≤28 weeks | 1 trial | 51 | 1.04 (0.48, 2.22) | 0.92 | N/A | N/A | 0.99 |
| | 29 to 32 weeks | 1 trial | 86 | 1.02 (0.58, 1.80) | 0.94 | N/A | N/A | |
| Cognitive impairment in adolescence | ≤28 weeks | 1 trial | 14 | 0.63 (0.12, 3.34) | 0.55 | N/A | N/A | 0.68 |
| | 29 to 32 weeks | 1 trial | 47 | 1.64 (0.57, 4.72) | 0.35 | N/A | N/A | |
| | 33 to 36 weeks | 1 trial | 8 | 0.67 (0.04, 13.09) | 0.72 | N/A | N/A | |
| Cognitive impairment at >3 years | ≤28 weeks | 2 trials | 65 | 0.97 (0.56, 1.69) | 0.93 | 0.69 | | 0.87 |
| | 29 to 32 weeks | 2 trials | 133 | 1.12 (0.74, 1.70) | 0.60 | 0.50 | | |
| | 33 to 36 weeks | 1 trial | 8 | 0.67 (0.04, 13.09) | 0.72 | N/A | N/A | |
| Metabolic risk in childhood | ≤28 weeks | 3 trials | 121 | 1.40 (0.83, 2.36) | 0.20 | 0.36 | 0.07 | 0.22 |
| | 29 to 32 weeks | 3 trials | 188 | 0.84 (0.59, 1.21) | 0.35 | 0.99 | 0.03 | |
| | 33 to 36 weeks | 2 trials | 25 | 1.07 (0.24, 4.82) | 0.93 | 0.93 | 0.52 | |
| Metabolic risk in adolescence | ≤28 weeks | 2 trials | 18 | 1.07 (0.37, 3.14) | 0.89 | 0.87 | 0.18 | 0.56 |
| | 29 to 32 weeks | 2 trials | 70 | 0.83 (0.61, 1.13) | 0.24 | 0.13 | 0.03 | |

| Outcome | Subgroup | No. of trials | No. of participants | aRR or aMD (95% CI) | P for overall effect | P for heterogeneity | Tau² | P for subgroup interaction |
|---|---|---|---|---|---|---|---|---|
| | 33 to 36 weeks | 2 trials | 16 | 0.58 (0.16, 2.11) | 0.38 | 0.77 | 0.18 | |
| Metabolic risk at >3 years | ≤28 weeks | 5 trials | 139 | 1.37 (0.88, 2.13) | 0.16 | 0.65 | 0.24 | 0.10 |
| | 29 to 32 weeks | 5 trials | 258 | 0.79 (0.61, 1.02) | 0.07 | 0.69 | 0.02 | |
| | 33 to 36 weeks | 4 trials | 41 | 0.73 (0.31, 1.67) | 0.44 | 0.54 | 0.17 | |
| Cognitive scores in toddlers | ≤28 weeks | 12 trials | 469 | 0.51 (-2.27, 3.28) | 0.72 | 0.66 | 1.99 | 0.53 |
| | 29 to 32 weeks | 10 trials | 544 | 0.62 (-1.83, 3.06) | 0.62 | 0.57 | 0.38 | |
| | 33 to 36 weeks | 7 trials | 157 | 3.28 (-1.95, 8.50) | 0.22 | 0.87 | 6.97 | |
| Cognitive scores in childhood | ≤28 weeks | 1 trial | 51 | -2.48 (-9.80, 4.84) | 0.50 | N/A | N/A | 0.94 |
| | 29 to 32 weeks | 1 trial | 86 | -2.96 (-6.93, 1.01) | 0.14 | N/A | N/A | |
| Cognitive scores in adolescence | ≤28 weeks | 1 trial | 14 | 1.00 (-18.86, 20.87) | 0.91 | N/A | N/A | 0.94 |
| | 29 to 32 weeks | 1 trial | 47 | 0.23 (-7.35, 7.79) | 0.95 | N/A | N/A | |
| | 33 to 36 weeks | 1 trial | 8 | 3.41 (-37.48, 44.30) | 0.83 | N/A | N/A | |
| Cognitive scores at >3 years | ≤28 weeks | 2 trials | 65 | -2.59 (-9.49, 4.30) | 0.45 | 0.82 | 0.08 | 0.87 |
| | 29 to 32 weeks | 2 trials | 133 | -1.80 (-5.46, 1.85) | 0.33 | 0.61 | 0.04 | |
| | 33 to 36 weeks | 1 trial | 8 | 3.41 (-37.48, 44.30) | 0.83 | N/A | 1.14 | |
| Motor impairment in toddlers | ≤28 weeks | 12 trials | 467 | 0.96 (0.75, 1.22) | 0.73 | 0.41 | 0.01 | 0.87 |
| | 29 to 32 weeks | 10 trials | 543 | 0.80 (0.60, 1.05) | 0.11 | 0.13 | 0.02 | |
| | 33 to 36 weeks | 7 trials | 156 | 0.67 (0.39, 1.17) | 0.16 | 0.96 | 0.08 | |

| Outcome | Subgroup | No. of trials | No. of participants | aRR or aMD (95% CI) | P for overall effect | P for heterogeneity | Tau² | P for subgroup interaction |
|---|---|---|---|---|---|---|---|---|
| Motor scores in toddlers | ≤28 weeks | 12 trials | 467 | 0.57 (-2.09, 3.23) | 0.67 | 0.99 | 0.32 | 0.37 |
| | 29 to 32 weeks | 10 trials | 543 | 1.78 (-0.46, 4.01) | 0.12 | 0.02 | 1.30 | |
| | 33 to 36 weeks | 7 trials | 156 | 4.35 (0.14, 8.56) | 0.04 | 0.72 | 4.54 | |
| SBP in childhood (mmHg) | ≤28 weeks | 2 trials | 77 | 0.31 (-4.55, 5.16) | 0.90 | 0.41 | 5.96 | 0.88 |
| | 29 to 32 weeks | 2 trials | 152 | 1.43 (-1.66, 4.53) | 0.36 | 0.14 | 2.47 | |
| | 33 to 36 weeks | 1 trial | 24 | -0.26 (-6.36, 5.84) | 0.93 | N/A | N/A | |
| SBP in adolescence (mmHg) | ≤28 weeks | 2 trials | 16 | 4.21 (-8.27, 16.68) | 0.47 | 0.56 | 32.15 | 0.43 |
| | 29 to 32 weeks | 2 trials | 69 | -0.27 (-4.55, 4.01) | 0.90 | 0.14 | 4.58 | |
| | 33 to 36 weeks | 2 trials | 16 | 9.59 (-0.59, 19.77) | 0.06 | 0.18 | 21.34 | |
| SBP at >3 years (mmHg) | ≤28 weeks | 4 trials | 93 | 0.16 (-4.17, 4.50) | 0.60 | 0.79 | 4.75 | 0.92 |
| | 29 to 32 weeks | 4 trials | 221 | 0.92 (-1.56, 3.41) | 0.46 | 0.26 | 1.59 | |
| | 33 to 36 weeks | 3 trials | 40 | 2.58 (-2.79, 7.96) | 0.34 | 0.15 | 6.97 | |
| DBP in childhood (mmHg) | ≤28 weeks | 2 trials | 77 | -1.65 (-6.19, 2.89) | 0.47 | 0.02 | 5.20 | 0.75 |
| | 29 to 32 weeks | 2 trials | 152 | -0.59 (-3.27, 2.09) | 0.67 | 0.06 | 1.85 | |
| | 33 to 36 weeks | 1 trial | 24 | 0.71 (-0.54, 6.77) | 0.81 | N/A | N/A | |
| DBP in adolescence (mmHg) | ≤28 weeks | 2 trials | 16 | 1.18 (-4.11, 7.66) | 0.52 | 0.67 | 29.27 | 0.98 |
| | 29 to 32 weeks | 2 trials | 69 | 0.13 (-2.98, 3.24) | 0.93 | 0.19 | 2.43 | |

| Outcome | Subgroup | No. of trials | No. of participants | aRR or aMD (95% CI) | P for overall effect | P for heterogeneity | Tau² | P for subgroup interaction |
|---|---|---|---|---|---|---|---|---|
| | 33 to 36 weeks | 2 trials | 16 | 1.18 (-4.11, 7.66) | 0.52 | 0.56 | 7.13 | |
| DBP at >3 years (mmHg) | ≤28 weeks | 4 trials | 93 | -1.23 (-5.39, 2.94) | 0.56 | 0.07 | 4.37 | 0.88 |
| | 29 to 32 weeks | 4 trials | 221 | -0.35 (-2.41, 1.72) | 0.74 | 0.14 | 1.10 | |
| | 33 to 36 weeks | 3 trials | 40 | 1.15 (-3.34, 5.65) | 0.61 | 0.86 | 4.88 | |
| MAP in childhood (mmHg) | ≤28 weeks | 2 trials | 81 | -0.56 (-4.69, 3.57) | 0.75 | 0.08 | 4.29 | 0.66 |
| | 29 to 32 weeks | 2 trials | 165 | -0.60 (-3.22, 2.02) | 0.59 | 0.13 | 1.77 | |
| | 33 to 36 weeks | 1 trial | 24 | 2.11 (-2.99, 7.22) | 0.40 | N/A | N/A | |
| MAP in adolescence (mmHg) | ≤28 weeks | 2 trials | 15 | 8.23 (-3.68, 20.15) | 0.25 | 0.16 | 28.62 | 0.66 |
| | 29 to 32 weeks | 2 trials | 68 | -0.81 (-4.34, 2.72) | 0.60 | 0.003 | 3.13 | |
| | 33 to 36 weeks | 2 trials | 16 | 3.99 (-4.42, 12.39) | 0.32 | 0.07 | 14.59 | |
| MAP at >3 years (mmHg) | ≤28 weeks | 4 trials | 96 | 0.07 (-3.74, 3.89) | 0.97 | 0.16 | 3.67 | 0.60 |
| | 29 to 32 weeks | 4 trials | 233 | -0.63 (-2.43, 1.46) | 0.54 | 0.14 | 1.15 | |
| | 33 to 36 weeks | 3 trials | 40 | 2.59 (-1.52, 6.70) | 0.21 | 0.73 | 4.08 | |
| Triglyceride concentrations in childhood (mmol/L) | ≤28 weeks | 1 trial | 24 | -0.004 (-0.17, 0.16) | 0.96 | N/A | N/A | 0.22 |
| | 29 to 32 weeks | 1 trial | 53 | -0.15 (-0.29, -0.008) | 0.04 | N/A | N/A | |
| Triglyceride concentrations in adolescence (mmol/L) | ≤28 weeks | 3 trials | 15 | -0.05 (-0.34, 0.23) | 0.68 | 0.53 | 0.02 | 0.49 |
| | 29 to 32 weeks | 3 trials | 52 | -0.18 (-0.62, 0.27) | 0.43 | 0.04 | 0.05 | |
| | 33 to 36 weeks | 2 trials | 11 | 0.37 (-0.50, 1.23) | 0.34 | 0.64 | 0.02 | |

| Outcome | Subgroup | No. of trials | No. of participants | aRR or aMD (95% CI) | P for overall effect | P for heterogeneity | Tau² | P for subgroup interaction |
|---|---|---|---|---|---|---|---|---|
| Triglyceride concentrations at >3 years (mmol/L) | ≤28 weeks | 3 trials | 39 | -0.02 (-0.15, 0.11) | 0.76 | 0.72 | 0.004 | 0.17 |
| | 29 to 32 weeks | 3 trials | 105 | -0.19 (-0.42, 0.04) | 0.11 | 0.05 | 0.11 | |
| | 33 to 36 weeks | 2 trials | 11 | 0.37 (-0.50, 1.23) | 0.34 | 0.64 | 0.12 | |
| Cholesterol concentrations in childhood (mmol/L) | ≤28 weeks | 1 trial | 25 | 0.37 (-0.09, 0.84) | 0.11 | N/A | N/A | 0.06 |
| | 29 to 32 weeks | 1 trial | 53 | -0.29 (-0.70, 0.13) | 0.17 | N/A | N/A | |
| Cholesterol concentrations in adolescence | ≤28 weeks | 3 trials | 15 | 0.13 (-0.39, 0.66) | 0.58 | 0.07 | 0.06 | 0.82 |
| | 29 to 32 weeks | 3 trials | 52 | 0.36 (-0.17, 0.90) | 0.18 | 0.42 | 0.07 | |
| | 33 to 36 weeks | 2 trials | 11 | 0.18 (-1.19, 1.54) | 0.56 | 0.94 | 0.30 | |
| Cholesterol concentrations at >3 years (mmol/L) | ≤28 weeks | 3 trials | 40 | 0.29 (-0.04, 0.62) | 0.08 | 0.29 | 0.03 | 0.85 |
| | 29 to 32 weeks | 3 trials | 105 | 0.07 (-0.26, 0.40) | 0.69 | 0.12 | 0.03 | |
| | 33 to 36 weeks | 2 trials | 11 | 0.18 (-1.19, 1.54) | 0.76 | 0.83 | 0.31 | |
| HDL concentrations in childhood (mmol/L) | ≤28 weeks | 1 trial | 24 | 0.12 (-0.10, 0.34) | 0.28 | N/A | N/A | 0.30 |
| | 29 to 32 weeks | 1 trial | 53 | -0.03 (-0.22, 0.15) | 0.70 | N/A | N/A | |
| HDL concentrations in adolescence (mmol/L) | ≤28 weeks | 3 trials | 15 | 0.17 (-0.16, 0.49) | 0.28 | 0.68 | 0.02 | 0.20 |
| | 29 to 32 weeks | 3 trials | 52 | -0.01 (-0.17, 0.15) | 0.91 | 0.17 | 0.06 | |
| | 33 to 36 weeks | 2 trials | 11 | -0.47 (-0.80, -0.13) | 0.01 | 0.05 | 0.02 | |
| | ≤28 weeks | 3 trials | 39 | 0.13 (-0.04, 0.30) | 0.12 | 0.98 | 0.06 | 0.08 |

| Outcome | Subgroup | No. of trials | No. of participants | aRR or aMD (95% CI) | P for overall effect | P for heterogeneity | Tau² | P for subgroup interaction |
|---|---|---|---|---|---|---|---|---|
| HDL concentrations at >3 years (mmol/L) | 29 to 32 weeks | 3 trials | 105 | -0.03 (-0.15, 0.08) | 0.58 | 0.75 | 0.004 | |
| | 33 to 36 weeks | 2 trials | 11 | -0.47 (-0.80, -0.13) | 0.01 | 0.05 | 0.02 | |
| LDL concentrations in childhood (mmol/L) | ≤28 weeks | 1 trial | 24 | 0.27 (-0.21, 0.75) | 0.25 | N/A | N/A | 0.13 |
| | 29 to 32 weeks | 1 trial | 53 | -0.17 (-0.52, 0.17) | 0.31 | N/A | N/A | |
| LDL concentrations in adolescence (mmol/L) | ≤28 weeks | 3 trials | 14 | -0.15 (-0.59, 0.29) | 0.47 | 0.28 | 0.04 | 0.39 |
| | 29 to 32 weeks | 3 trials | 52 | 0.19 (-0.16, 0.54) | 0.27 | 0.91 | 0.03 | |
| | 33 to 36 weeks | 2 trials | 11 | 0.46 (-0.84, 1.76) | 0.42 | 0.62 | 0.28 | |
| LDL concentrations at >3 years (mmol/L) | ≤28 weeks | 3 trials | 38 | 0.12 (-0.21, 0.46) | 0.47 | 0.34 | 0.03 | 0.56 |
| | 29 to 32 weeks | 3 trials | 105 | 0.01 (-0.23, 0.25) | 0.93 | 0.31 | 0.01 | |
| | 33 to 36 weeks | 2 trials | 11 | 0.46 (-0.84, 1.76) | 0.42 | 0.62 | 0.28 | |
| Blood glucose concentrations in childhood (mmol/L) | ≤28 weeks | 1 trial | 24 | 0.18 (-0.42, 0.79) | 0.53 | N/A | N/A | 0.36 |
| | 29 to 32 weeks | 1 trial | 47 | -0.09 (-0.43, 0.24) | 0.58 | N/A | N/A | |
| Blood glucose concentrations in adolescence (mmol/L) | ≤28 weeks | 3 trials | 15 | -0.08 (-0.40, 0.23) | 0.57 | 0.04 | 0.02 | 0.19 |
| | 29 to 32 weeks | 3 trials | 52 | -0.21 (-0.41, -0.01) | 0.04 | 0.05 | 0.01 | |
| | 33 to 36 weeks | 2 trials | 11 | 0.35 (0.01, 0.68) | 0.04 | 0.09 | 0.02 | |
| | ≤28 weeks | 3 trials | 39 | 0.07 (-0.30, 0.45) | 0.70 | 0.61 | 0.03 | 0.25 |
| | 29 to 32 weeks | 3 trials | 99 | -0.17 (-0.35, 0.02) | 0.07 | 0.81 | 0.01 | |

| Outcome | Subgroup | No. of trials | No. of participants | aRR or aMD (95% CI) | P for overall effect | P for heterogeneity | Tau² | P for subgroup interaction |
|---|---|---|---|---|---|---|---|---|
| Blood glucose concentrations at >3 years (mmol/L) | 33 to 36 weeks | 2 trials | 11 | 0.35 (0.01, 0.68) | 0.04 | 0.09 | 0.02 | |
| BMI in childhood (kg/m²) | ≤28 weeks | 3 trials | 121 | 0.62 (-0.03, 1.26) | 0.06 | 0.15 | 0.11 | 0.01 |
| | 29 to 32 weeks | 3 trials | 187 | -0.46 (-0.93, 0.01) | 0.06 | <.0001 | 0.06 | |
| | 33 to 36 weeks | 2 trials | 25 | -0.84 (-1.91, 0.23) | 0.51 | 0.13 | 0.03 | |
| BMI in adolescence (kg/m²) | ≤28 weeks | 2 trials | 17 | -2.07 (-5.44, 1.30) | 0.21 | 0.62 | 2.40 | 0.25 |
| | 29 to 32 weeks | 2 trials | 70 | 0.27 (-1.52, 2.06) | 0.76 | 0.006 | 0.81 | |
| | 33 to 36 weeks | 2 trials | 16 | 2.81 (-1.16, 6.78) | 0.15 | 0.36 | 3.24 | |
| BMI at >3 years (kg/m²) | ≤28 weeks | 5 trials | 138 | 0.33 (-0.35, 1.01) | 0.34 | 0.04 | 0.11 | 0.44 |
| | 29 to 32 weeks | 5 trials | 257 | -0.26 (-0.85, 0.32) | 0.30 | <.0001 | 0.09 | |
| | 33 to 36 weeks | 4 trials | 41 | 0.12 (-1.46, 1.70) | 0.78 | 0.04 | 0.01 | |
| BMI z-score in childhood | ≤28 weeks | 3 trials | 121 | 0.38 (-0.03, 0.79) | 0.06 | 0.01 | 0.04 | 0.01 |
| | 29 to 32 weeks | 3 trials | 187 | -0.33 (-0.62, -0.03) | 0.03 | <.0001 | 0.02 | |
| | 33 to 36 weeks | 2 trials | 25 | -0.63 (-1.34, 0.09) | 0.08 | 0.09 | 0.12 | |
| BMI z-score in adolescence | ≤28 weeks | 2 trials | 17 | 0.83 (-1.00, 2.67) | 0.34 | 0.34 | 0.40 | 0.56 |
| | 29 to 32 weeks | 2 trials | 70 | -0.08 (-0.71, 0.55) | 0.80 | 0.002 | 0.10 | |
| | 33 to 36 weeks | 2 trials | 16 | -0.51 (-1.89, 0.87) | 0.44 | 0.51 | 0.69 | |
| | ≤28 weeks | 5 trials | 138 | 0.27 (-0.11, 0.66) | 0.27 | 0.13 | 0.04 | 0.10 |

| Outcome | Subgroup | No. of trials | No. of participants | aRR or aMD (95% CI) | P for overall effect | P for heterogeneity | Tau² | P for subgroup interaction |
|---|---|---|---|---|---|---|---|---|
| BMI z-score at >3 years | 29 to 32 weeks | 5 trials | 257 | -0.26 (-0.54, 0.01) | 0.06 | <.0001 | 0.02 | |
| | 33 to 36 weeks | 4 trials | 41 | 0.34 (-0.82, 0.55) | 0.69 | 0.05 | 0.04 | |
| Fasting insulin in childhood (pmol/L) | ≤28 weeks | 1 trial | 34 | 0.65 (-16.01, 17.31) | 0.94 | N/A | N/A | 0.42 |
| | 29 to 32 weeks | 1 trial | 54 | 6.46 (-6.61, 19.53) | 0.33 | N/A | N/A | |
| Fasting insulin in adolescence (pmol/L) | ≤28 weeks | 3 trials | 15 | -13.25 (-35.27, 8.76) | 0.21 | 0.25 | 97.61 | 0.18 |
| | 29 to 32 weeks | 3 trials | 48 | -5.88 (-24.64, 12.88) | 0.53 | 0.41 | 86.49 | |
| | 33 to 36 weeks | 2 trials | 13 | 30.03 (-15.88, 75.94) | 0.17 | 0.18 | 396.01 | |
| Fasting insulin at >3 years (pmol/L) | ≤28 weeks | 3 trials | 49 | -4.06 (16.59, 8.47) | 0.52 | 0.37 | 38.56 | 0.17 |
| | 29 to 32 weeks | 3 trials | 102 | -0.60 (-11.54, 10.35) | 0.91 | 0.39 | 30.36 | |
| | 33 to 36 weeks | 2 trials | 13 | 30.03 (-15.88, 75.94) | 0.17 | 0.18 | 396.41 | |
| IGF-I in adolescence (nmol/L) | ≤28 weeks | 2 trials | 15 | -10.57 (-34.05, 13.67) | 0.34 | 0.0002 | 111.73 | 0.26 |
| | 29 to 32 weeks | 2 trials | 49 | 1.47 (-9.12, 12.07) | 0.78 | 0.55 | 27.67 | |
| | 33 to 36 weeks | 2 trials | 13 | 12.27 (-22.91, 47.44) | 0.45 | 0.10 | 232.56 | |

Abbreviation: SBP: systolic blood pressure; DBP diastolic blood pressure; MAP: mean arterial pressure; HDL: high-density lipoproteins; LDL: low-density lipoproteins; BMI: body mass index; aRR: adjusted relative risk; aMD: adjusted mean difference; N/A: not applicable
Relative risk and mean difference were adjusted for sex and birthweight z-scores.

**Table S5. Subgroup analyses of timing of supplements**

| Outcome | Subgroup | No. of trials | No. of participants | aRR or aMD (95% CI) | P for overall effect | P for heterogeneity | Tau² | P for subgroup interaction |
|---|---|---|---|---|---|---|---|---|
| Cognitive impairment in toddlers | In hospital | 7 trials | 653 | 0.91 (0.69, 1.20) | 0.50 | 0.83 | 0.02 | 0.89 |
| | Post discharge | 6 trials | 757 | 1.02 (0.75, 1.38) | 0.89 | 0.69 | 0.02 | |
| Metabolic risk in childhood | In hospital | 1 trial | 55 | 0.40 (0.14, 1.10) | 0.07 | N/A | N/A | 0.04 |
| | Post discharge | 2 trials | 279 | 1.16 (0.86, 1.56) | 0.32 | 0.53 | 0.02 | |
| Metabolic risk in adolescence | In hospital | 1 trial | 36 | 0.59 (0.38, 0.92) | 0.02 | N/A | N/A | 0.04 |
| | Post discharge | 1 trial | 68 | 1.11 (0.77, 1.59) | 0.58 | N/A | N/A | |
| Metabolic risk at >3 years | In hospital | 2 trials | 91 | 0.55 (0.34, 0.89) | 0.02 | 0.43 | 0.06 | 0.003 |
| | Post discharge | 3 trials | 347 | 1.12 (0.88, 1.42) | 0.37 | 0.80 | 0.01 | |
| Cognitive scores in toddlers | In hospital | 7 trials | 653 | 1.31 (-1.25, 3.86) | 0.32 | 0.73 | 1.69 | 0.58 |
| | Post discharge | 6 trials | 757 | 0.02 (-1.83, 1.87) | 0.99 | 0.58 | 0.88 | |
| Motor impairment in toddlers | In hospital | 7 trials | 652 | 0.95 (0.78, 1.17) | 0.65 | 0.97 | 0.01 | 0.31 |
| | Post discharge | 6 trials | 754 | 0.82 (0.56, 1.21) | 0.32 | 0.84 | 0.04 | |
| Motor scores in toddlers | In hospital | 7 trials | 652 | 1.16 (-0.63, 3.94) | 0.16 | 0.92 | 1.35 | 0.97 |

| Outcome | Subgroup | No. of trials | No. of participants | aRR or aMD (95% CI) | P for overall effect | P for heterogeneity | Tau² | P for subgroup interaction |
|---|---|---|---|---|---|---|---|---|
| | Post discharge | 6 trials | 754 | 1.32 (-0.44, 3.08) | 0.14 | 0.008 | 0.80 | |
| SBP in childhood (mmHg) | In hospital | None | N/A | N/A | N/A | N/A | N/A | N/A |
| | Post discharge | 2 trials | 253 | 0.95 (-1.41, 3.32) | 0.43 | 0.46 | 1.42 | |
| SBP in adolescence (mmHg) | In hospital | 1 trial | 36 | -2.95 (-9.22, 3.32) | 0.34 | N/A | N/A | 0.11 |
| | Post discharge | 1 trial | 65 | 3.64 (-1.06, 8.35) | 0.54 | N/A | N/A | |
| SBP at >3 years (mmHg) | In hospital | 1 trial | 36 | -2.95 (-9.22, 3.32) | 0.34 | N/A | N/A | 0.19 |
| | Post discharge | 3 trials | 318 | 1.41 (-0.68, 3.51) | 0.19 | 0.55 | 1.14 | |
| DBP in childhood (mmHg) | In hospital | None | N/A | N/A | N/A | N/A | N/A | N/A |
| | Post discharge | 2 trials | 253 | -0.60 (-2.75, 1.54) | 0.58 | 0.83 | 1.19 | |
| DBP in adolescence (mmHg) | In hospital | 1 trial | 36 | -1.07 (-6.22, 4.08) | 0.67 | N/A | N/A | 0.26 |
| | Post discharge | 1 trial | 65 | 1.72 (-1.87, 5.31) | 0.34 | N/A | N/A | |
| DBP at >3 years (mmHg) | In hospital | 1 trial | 36 | -1.07 (-6.22, 4.08) | 0.67 | N/A | N/A | 0.55 |
| | Post discharge | 3 trials | 318 | -0.14 (-1.98, 1.71) | 0.89 | 0.61 | 0.88 | |

| Outcome | Subgroup | No. of trials | No. of participants | aRR or aMD (95% CI) | P for overall effect | P for heterogeneity | Tau² | P for subgroup interaction |
|---|---|---|---|---|---|---|---|---|
| MAP in childhood (mmHg) | In hospital | None | N/A | N/A | N/A | N/A | N/A | N/A |
| | Post discharge | 2 trials | 270 | -0.31 (-2.34, 1.72) | 0.76 | 0.82 | 1.06 | |
| MAP in adolescence (mmHg) | In hospital | 1 trial | 34 | -3.97 (-9.55, 1.61) | 0.16 | N/A | N/A | 0.06 |
| | Post discharge | 1 trial | 65 | 2.36 (-1.38, 6.11) | 0.21 | N/A | N/A | |
| MAP at >3 years (mmHg) | In hospital | 1 trial | 34 | -3.97 (-9.55, 1.61) | 0.16 | N/A | N/A | 0.16 |
| | Post discharge | 3 trials | 335 | 0.18 (-1.59, 1.96) | 0.84 | 0.52 | 0.81 | |
| Triglyceride concentrations in childhood (mmol/L) | In hospital | none | N/A | N/A | N/A | N/A | N/A | N/A |
| | Post discharge | 1 trial | 77 | -0.12 (-0.22, -0.01) | 0.03 | N/A | N/A | |
| Triglyceride concentrations in adolescence (mmol/L) | In hospital | 1 trial | 27 | -0.54 (-1.49, 0.40) | 0.25 | N/A | N/A | 0.07 |
| | Post discharge | 1 trial | 51 | 0.09 (-0.11, 0.29) | 0.37 | N/A | N/A | |
| Triglyceride concentrations at >3 years (mmol/L) | In hospital | 1 trial | 27 | -0.50 (-0.90, -0.10) | 0.01 | N/A | N/A | 0.04 |
| | Post discharge | 2 trials | 128 | -0.04 (-0.22, 0.14) | 0.67 | 0.05 | 0.003 | |
| | In hospital | none | N/A | N/A | N/A | N/A | N/A | N/A |

| Outcome | Subgroup | No. of trials | No. of participants | aRR or aMD (95% CI) | P for overall effect | P for heterogeneity | Tau² | P for subgroup interaction |
|---|---|---|---|---|---|---|---|---|
| Cholesterol concentrations in childhood (mmol/L) | Post discharge | 1 trial | 78 | -0.12 (-0.45, 0.20) | 0.45 | N/A | N/A | |
| Cholesterol concentrations in adolescence (mmol/L) | In hospital | 1 trial | 27 | 0.64 (-0.29, 1.56) | 0.17 | N/A | N/A | 0.42 |
| | Post discharge | 1 trial | 51 | 0.18 (-0.22, 0.58) | 0.38 | N/A | N/A | |
| Cholesterol concentrations at >3 years | In hospital | 1 trial | 27 | 0.64 (-0.29, 1.56) | 0.17 | N/A | N/A | 0.13 |
| | Post discharge | 2 trials | 129 | 0.01 (-0.24, 0.26) | 0.94 | 0.21 | 0.01 | |
| HDL concentrations in childhood (mmol/L) | In hospital | none | N/A | N/A | N/A | N/A | N/A | N/A |
| | Post discharge | 1 trial | 77 | 0.02 (-0.12, 0.16) | 0.74 | N/A | N/A | |
| HDL concentrations in adolescence (mmol/L) | In hospital | 1 trial | 27 | -0.02 (-0.31, 0.27) | 0.89 | N/A | N/A | 0.97 |
| | Post discharge | 1 trial | 51 | -0.03 (-0.18, 0.12) | 0.70 | N/A | N/A | |
| HDL concentrations at >3 years (mmol/L) | In hospital | 1 trial | 27 | -0.02 (-0.31, 0.27) | 0.89 | N/A | N/A | 0.82 |
| | Post discharge | 2 trials | 128 | 0.01 (-0.09, 0.11) | 0.86 | 0.64 | 0.03 | |
| LDL concentrations in childhood (mmol/L) | In hospital | none | N/A | N/A | N/A | N/A | N/A | N/A |
| | Post discharge | 1 trial | 77 | -0.08 (-0.36, 0.19) | 0.56 | N/A | N/A | |

| Outcome | Subgroup | No. of trials | No. of participants | aRR or aMD (95% CI) | P for overall effect | P for heterogeneity | Tau² | P for subgroup interaction |
|---|---|---|---|---|---|---|---|---|
| LDL concentrations in adolescence (mmol/L) | In hospital | 1 trial | 27 | 0.27 (-0.16, 0.70) | 0.21 | N/A | N/A | 0.85 |
| | Post discharge | 1 trial | 50 | 0.16 (-0.21, 0.53) | 0.38 | N/A | N/A | |
| LDL concentrations at >3 years (mmol/L) | In hospital | 1 trial | 27 | 0.27 (-0.16, 0.70) | 0.21 | N/A | N/A | 0.72 |
| | Post discharge | 2 trials | 127 | 0.02 (-0.19, 0.24) | 0.82 | 0.25 | 0.01 | |
| Blood glucose concentrations in childhood (mmol/L) | In hospital | none | N/A | N/A | N/A | N/A | N/A | N/A |
| | Post discharge | 1 trial | 71 | -0.02 (-0.30, 0.27) | 0.91 | N/A | N/A | |
| Blood glucose concentrations in adolescence (mmol/L) | In hospital | 1 trial | 27 | -0.34 (-0.66, -0.02) | 0.04 | N/A | N/A | 0.13 |
| | Post discharge | 1 trial | 51 | -0.03 (-0.21, 0.14) | 0.67 | N/A | N/A | |
| Blood glucose concentrations at >3 years (mmol/L) | In hospital | 1 trial | 27 | -0.34 (-0.66, -0.02) | 0.04 | N/A | N/A | 0.24 |
| | Post discharge | 2 trials | 122 | -0.04 (-0.21, 0.14) | 0.70 | 0.79 | 0.01 | |
| BMI in childhood ($kg/m^2$) | In hospital | 1 trial | 55 | -0.61 (-1.80, 0.57) | 0.30 | N/A | N/A | 0.23 |
| | Post discharge | 2 trials | 278 | -0.01 (-0.37, 0.35) | 0.97 | 0.46 | 0.03 | |
| | In hospital | 1 trial | 36 | -2.49 (-4.51, -0.48) | 0.02 | N/A | N/A | 0.02 |

| Outcome | Subgroup | No. of trials | No. of participants | aRR or aMD (95% CI) | P for overall effect | P for heterogeneity | Tau² | P for subgroup interaction |
|---|---|---|---|---|---|---|---|---|
| BMI in adolescence (kg/m²) | Post discharge | 1 trial | 67 | 1.40 (-0.48, 3.27) | 0.14 | N/A | N/A | |
| BMI at >3 years (kg/m²) | In hospital | 2 trials | 91 | -1.17 (-2.25, -0.10) | 0.03 | 0.18 | 0.29 | 0.01 |
| | Post discharge | 3 trials | 345 | 0.26 (-0.20, 0.71) | 0.27 | 0.06 | 0.05 | |
| BMI z-score in childhood | In hospital | 1 trial | 55 | -0.29 (-1.01, 0.43) | 0.43 | N/A | N/A | 0.48 |
| | Post discharge | 2 trials | 278 | -0.06 (-0.29, 0.18) | 0.63 | 0.51 | 0.01 | |
| BMI z-score in adolescence | In hospital | 1 trial | 36 | -1.20 (-2.08, -0.31) | 0.01 | N/A | N/A | 0.01 |
| | Post discharge | 1 trial | 67 | 0.47 (-0.17, 1.11) | 0.15 | N/A | N/A | |
| BMI z-score at >3 years | In hospital | 2 trials | 91 | -0.55 (-1.11, 0.02) | 0.06 | 0.22 | 0.08 | 0.02 |
| | Post discharge | 3 trials | 345 | 0.04 (-0.18, 0.26) | 0.73 | 0.18 | 0.01 | |
| Fasting insulin in childhood (pmol/L) | In hospital | none | N/A | N/A | N/A | N/A | N/A | N/A |
| | Post discharge | 1 trial | 88 | 2.48 (-7.51, 12.47) | 0.62 | N/A | N/A | |
| Fasting insulin in adolescence (pmol/L) | In hospital | 1 trial | 27 | -27.74 (-47.54, -1.94) | 0.03 | N/A | N/A | 0.34 |
| | Post discharge | 1 trial | 49 | 2.29 (-15.09, 19.68) | 0.79 | N/A | N/A | |

| Outcome | Subgroup | No. of trials | No. of participants | aRR or aMD (95% CI) | P for overall effect | P for heterogeneity | Tau² | P for subgroup interaction |
|---|---|---|---|---|---|---|---|---|
| Fasting insulin at >3 years (pmol/L) | In hospital | 1 trial | 27 | -27.74 (-47.54, -1.94) | 0.03 | N/A | N/A | 0.21 |
| | Post discharge | 2 trials | 137 | 1.68 (-7.04, 10.39) | 0.70 | 0.87 | 19.36 | |
| IGF-I in adolescence (nmol/L) | In hospital | 1 trial | 28 | -8.41 (-21.91, 5.09) | 0.21 | N/A | N/A | 0.87 |
| | Post discharge | 1 trial | 49 | 3.45 (-8.11, 15.01) | 0.55 | N/A | N/A | |

Abbreviation: SBP: systolic blood pressure; DBP diastolic blood pressure; MAP: mean arterial pressure; HDL: high-density lipoproteins; LDL: low-density lipoproteins; BMI: body mass index; aRR: adjusted relative risk; aMD: adjusted mean difference; N/A: not applicable.
Relative risk and mean difference were adjusted for sex, gestational age and birthweight z-scores.

**Table S6. Subgroup analyses of type of supplement**

| Outcome | Subgroup | No. of trials | No. of participants | aRR or aMD (95% CI) | P for overall effect | P for heterogeneity | Tau² | P for subgroup interaction |
|---|---|---|---|---|---|---|---|---|
| Cognitive impairment in toddlers | Protein | 1 trial | 55 | 1.58 (0.28, 8.81) | 0.59 | N/A | N/A | 0.37 |
| | Multicomponent | 12 trials | 1355 | 0.92 (0.73, 1.17) | 0.51 | 0.90 | 0.01 | |
| Metabolic risk in childhood | Protein | 1 trial | 55 | 0.40 (0.14, 1.10) | 0.07 | N/A | N/A | 0.04 |
| | Multicomponent | 2 trials | 279 | 1.16 (0.86, 1.56) | 0.32 | 0.53 | 0.02 | |
| Metabolic risk in adolescence | Protein | 1 trial | 36 | 0.59 (0.38, 0.92) | 0.02 | N/A | N/A | 0.04 |
| | Multicomponent | 1 trial | 68 | 1.11 (0.77, 1.59) | 0.58 | N/A | N/A | |
| Metabolic risk at >3 years | Protein | 2 trials | 91 | 0.55 (0.34, 0.89) | 0.02 | 0.43 | 0.06 | 0.003 |
| | Multicomponent | 3 trials | 347 | 1.12 (0.88, 1.42) | 0.37 | 0.80 | 0.01 | |
| Cognitive scores in toddlers | Protein | 1 trial | 55 | 1.86 (-6.53, 10.25) | 0.66 | N/A | N/A | 0.86 |
| | Multicomponent | 12 trials | 1355 | 0.71 (-0.88, 2.30) | 0.38 | 0.71 | 0.64 | |
| Motor impairment in toddlers | Protein | 1 trial | 55 | 0.82 (0.12, 5.75) | 0.84 | N/A | N/A | 0.78 |
| | Multicomponent | 12 trials | 1351 | 0.82 (0.65, 1.04) | 0.10 | 0.98 | 0.01 | |
| | Protein | 1 trial | 55 | 4.56 (-5.11, 14.22) | 0.35 | N/A | N/A | 0.46 |

| Outcome | Subgroup | No. of trials | No. of participants | aRR or aMD (95% CI) | P for overall effect | P for heterogeneity | $Tau^2$ | P for subgroup interaction |
|---|---|---|---|---|---|---|---|---|
| Motor scores in toddlers | Multicomponent | 12 trials | 1351 | 0.73 (0.02, 2.87) | 0.05 | 0.19 | 0.53 | |
| SBP in childhood (mmHg) | Protein | None | N/A | N/A | N/A | N/A | N/A | N/A |
| | Multicomponent | 2 trials | 253 | 0.95 (-1.41, 3.32) | 0.43 | 0.46 | 1.42 | |
| SBP in adolescence (mmHg) | Protein | 1 trial | 36 | -2.95 (-9.22, 3.32) | 0.34 | N/A | N/A | 0.11 |
| | Multicomponent | 1 trial | 65 | 3.64 (-1.06, 8.35) | 0.54 | N/A | N/A | |
| SBP at >3 years (mmHg) | Protein | 1 trial | 36 | -2.95 (-9.22, 3.32) | 0.34 | N/A | N/A | 0.19 |
| | Multicomponent | 3 trials | 318 | 1.41 (-0.68, 3.51) | 0.19 | 0.55 | 1.14 | |
| DBP in childhood (mmHg) | Protein | None | N/A | N/A | N/A | N/A | N/A | N/A |
| | Multicomponent | 2 trials | 253 | -0.60 (-2.75, 1.54) | 0.58 | 0.83 | 1.19 | |
| DBP in adolescence (mmHg) | Protein | 1 trial | 36 | -1.07 (-6.22, 4.08) | 0.67 | N/A | N/A | 0.26 |
| | Multicomponent | 1 trial | 65 | 1.72 (-1.87, 5.31) | 0.34 | N/A | N/A | |
| DBP at >3 years (mmHg) | Protein | 1 trial | 36 | -1.07 (-6.22, 4.08) | 0.67 | N/A | N/A | 0.55 |
| | Multicomponent | 3 trials | 318 | -0.14 (-1.98, 1.71) | 0.89 | 0.61 | 0.88 | |

| Outcome | Subgroup | No. of trials | No. of participants | aRR or aMD (95% CI) | P for overall effect | P for heterogeneity | Tau² | P for subgroup interaction |
|---|---|---|---|---|---|---|---|---|
| MAP in childhood (mmHg) | Protein | None | N/A | N/A | N/A | N/A | N/A | N/A |
| | Multicomponent | 2 trials | 270 | -0.31 (-2.34, 1.72) | 0.76 | 0.82 | 1.06 | |
| MAP in adolescence (mmHg) | Protein | 1 trial | 34 | -3.97 (-9.55, 1.61) | 0.16 | N/A | N/A | 0.06 |
| | Multicomponent | 1 trial | 65 | 2.36 (-1.38, 6.11) | 0.21 | N/A | N/A | |
| MAP at >3 years (mmHg) | Protein | 1 trial | 34 | -3.97 (-9.55, 1.61) | 0.16 | N/A | N/A | 0.16 |
| | Multicomponent | 3 trials | 335 | 0.18 (-1.59, 1.96) | 0.84 | 0.52 | 0.81 | |
| Triglyceride concentrations in childhood (mmol/L) | Protein | none | N/A | N/A | N/A | N/A | N/A | N/A |
| | Multicomponent | 1 trial | 77 | -0.12 (-0.22, -0.01) | 0.03 | N/A | N/A | |
| Triglyceride concentrations in adolescence (mmol/L) | Protein | 1 trial | 27 | -0.54 (-1.49, 0.40) | 0.25 | N/A | N/A | 0.07 |
| | Multicomponent | 1 trial | 51 | 0.09 (-0.11, 0.29) | 0.37 | N/A | N/A | |
| Triglyceride concentrations at >3 years (mmol/L) | Protein | 1 trial | 27 | -0.54 (-1.49, 0.40) | 0.25 | N/A | N/A | 0.04 |
| | Multicomponent | 2 trials | 128 | -0.04 (-0.15, 0.06) | 0.41 | 0.05 | 0.003 | |
| | Protein | none | N/A | N/A | N/A | N/A | N/A | N/A |

| Outcome | Subgroup | No. of trials | No. of participants | aRR or aMD (95% CI) | P for overall effect | P for heterogeneity | Tau² | P for subgroup interaction |
|---|---|---|---|---|---|---|---|---|
| Cholesterol concentrations in childhood (mmol/L) | Multicomponent | 1 trial | 78 | -0.12 (-0.45, 0.20) | 0.45 | N/A | N/A | |
| Cholesterol concentrations in adolescence (mmol/L) | Protein | 1 trial | 27 | 0.64 (-0.29, 1.56) | 0.17 | N/A | N/A | 0.42 |
| | Multicomponent | 1 trial | 51 | 0.18 (-0.22, 0.58) | 0.38 | N/A | N/A | |
| Cholesterol concentrations at >3 years (mmol/L) | Protein | 1 trial | 27 | 0.64 (-0.29, 1.56) | 0.17 | N/A | N/A | 0.13 |
| | Multicomponent | 2 trials | 129 | 0.01 (-0.24, 0.26) | 0.94 | 0.21 | 0.01 | |
| HDL concentrations in childhood (mmol/L) | Protein | none | N/A | N/A | N/A | N/A | N/A | N/A |
| | Multicomponent | 1 trial | 77 | 0.02 (-0.12, 0.16) | 0.74 | N/A | N/A | |
| HDL concentrations in adolescence (mmol/L) | Protein | 1 trial | 27 | -0.02 (-0.31, 0.27) | 0.89 | N/A | N/A | 0.97 |
| | Multicomponent | 1 trial | 51 | -0.03 (-0.18, 0.12) | 0.70 | N/A | N/A | |
| HDL concentrations at >3 years (mmol/L) | Protein | 1 trial | 27 | -0.02 (-0.31, 0.27) | 0.89 | N/A | N/A | 0.82 |
| | Multicomponent | 2 trials | 128 | 0.01 (-0.09, 0.11) | 0.86 | 0.64 | 0.03 | |
| LDL concentrations in childhood (mmol/L) | Protein | none | N/A | N/A | N/A | N/A | N/A | N/A |
| | Multicomponent | 1 trial | 77 | -0.08 (-0.36, 0.19) | 0.56 | N/A | N/A | |

| Outcome | Subgroup | No. of trials | No. of participants | aRR or aMD (95% CI) | P for overall effect | P for heterogeneity | Tau² | P for subgroup interaction |
|---|---|---|---|---|---|---|---|---|
| LDL concentrations in adolescence (mmol/L) | Protein | 1 trial | 27 | 0.27 (-0.16, 0.70) | 0.21 | N/A | N/A | 0.85 |
| | Multicomponent | 1 trial | 50 | 0.16 (-0.21, 0.53) | 0.38 | N/A | N/A | |
| LDL concentrations at >3 years (mmol/L) | Protein | 1 trial | 27 | 0.27 (-0.16, 0.70) | 0.21 | N/A | N/A | 0.72 |
| | Multicomponent | 2 trials | 127 | 0.02 (-0.19, 0.24) | 0.82 | 0.25 | 0.01 | |
| Blood glucose concentrations in childhood (mmol/L) | Protein | none | N/A | N/A | N/A | N/A | N/A | N/A |
| | Multicomponent | 1 trial | 71 | -0.02 (-0.30, 0.27) | 0.91 | N/A | N/A | |
| Blood glucose concentrations in adolescence (mmol/L) | Protein | 1 trial | 27 | -0.34 (-0.66, -0.02) | 0.04 | N/A | N/A | 0.13 |
| | Multicomponent | 1 trial | 51 | -0.03 (-0.21, 0.14) | 0.67 | N/A | N/A | |
| Blood glucose concentrations at >3 years (mmol/L) | Protein | 1 trial | 27 | -0.34 (-0.66, -0.02) | 0.04 | N/A | N/A | 0.24 |
| | Multicomponent | 2 trials | 122 | -0.04 (-0.21, 0.14) | 0.70 | 0.79 | 0.01 | |
| BMI in childhood (kg/m²) | Protein | 1 trial | 55 | -0.61 (-1.80, 0.57) | 0.30 | N/A | N/A | 0.23 |
| | Multicomponent | 2 trials | 278 | -0.01 (-0.37, 0.35) | 0.97 | 0.46 | 0.03 | |
| | Protein | 1 trial | 36 | -2.49 (-4.51, -0.48) | 0.02 | N/A | N/A | 0.02 |

| Outcome | Subgroup | No. of trials | No. of participants | aRR or aMD (95% CI) | P for overall effect | P for heterogeneity | Tau² | P for subgroup interaction |
|---|---|---|---|---|---|---|---|---|
| BMI in adolescence (kg/m²) | Multicomponent | 1 trial | 67 | 1.40 (-0.48, 3.27) | 0.14 | N/A | N/A | |
| BMI at >3 years (kg/m²) | Protein | 2 trials | 91 | -1.17 (-2.25, -0.10) | 0.03 | 0.18 | 0.29 | 0.01 |
| | Multicomponent | 3 trials | 345 | 0.26 (-0.20, 0.71) | 0.27 | 0.06 | 0.05 | |
| BMI z-score in childhood | Protein | 1 trial | 55 | -0.29 (-1.01, 0.43) | 0.43 | N/A | N/A | 0.48 |
| | Multicomponent | 2 trials | 278 | -0.06 (-0.29, 0.18) | 0.63 | 0.51 | 0.01 | |
| BMI z-score in adolescence | Protein | 1 trial | 36 | -1.20 (-2.08, -0.31) | 0.01 | N/A | N/A | 0.01 |
| | Multicomponent | 1 trial | 67 | 0.47 (-0.17, 1.11) | 0.15 | N/A | N/A | |
| BMI z-score at >3 years | Protein | 2 trials | 91 | -0.55 (-1.11, 0.02) | 0.06 | 0.22 | 0.08 | 0.02 |
| | Multicomponent | 3 trials | 345 | 0.04 (-0.18, 0.26) | 0.73 | 0.18 | 0.01 | |
| Fasting insulin in childhood (pmol/L) | Protein | none | | | | | N/A | N/A |
| | Multicomponent | 1 trial | 88 | 2.48 (-7.51, 12.47) | 0.62 | N/A | N/A | |
| Fasting insulin in adolescence (pmol/L) | Protein | 1 trial | 27 | -27.74 (-47.54, -1.94) | 0.03 | N/A | N/A | 0.34 |
| | Multicomponent | 1 trial | 49 | 2.29 (-15.09, 19.68) | 0.79 | N/A | N/A | |

| Outcome | Subgroup | No. of trials | No. of participants | aRR or aMD (95% CI) | P for overall effect | P for heterogeneity | Tau² | P for subgroup interaction |
|---|---|---|---|---|---|---|---|---|
| Fasting insulin at >3 years (pmol/L) | Protein | 1 trial | 27 | -27.74 (-47.54, -1.94) | 0.03 | N/A | N/A | 0.21 |
| | Multicomponent | 2 trials | 137 | 1.68 (-7.04, 10.39) | 0.70 | 0.87 | 19.36 | |
| IGF-I in adolescence (nmol/L) | Protein | 1 trial | 28 | -8.41 (-21.91, 5.09) | 0.21 | N/A | N/A | 0.87 |
| | Multicomponent | 1 trial | 49 | 3.45 (-8.11, 15.01) | 0.55 | N/A | N/A | |

Abbreviation: SBP: systolic blood pressure; DBP diastolic blood pressure; MAP: mean arterial pressure; HDL: high-density lipoproteins; LDL: low-density lipoproteins; BMI: body mass index; aRR: adjusted relative risk; aMD: adjusted mean difference; N/A: not applicable
Relative risk and mean difference were adjusted for sex, gestational age and birthweight z-scores.

**Table S7. Subgroup analyses of primary milk feed**

| Outcome | Subgroup | No. of trials | No. of participants | aRR or aMD (95% CI) | P for overall effect | P for heterogeneity | Tau² | P for subgroup interaction |
|---|---|---|---|---|---|---|---|---|
| Cognitive impairment in toddlers | BM | 6 trials | 541 | 1.03 (0.72, 1.48) | 0.87 | 0.84 | 0.03 | 0.54 |
| | Formula | 5 trials | 704 | 1.02 (0.74, 1.39) | 0.92 | 0.56 | 0.02 | |
| | PN+EN | 2 trials | 165 | 0.76 (0.54, 1.07) | 0.12 | 0.77 | 0.03 | |
| Cognitive impairment at >3 years | BM | 1 trial | 137 | 1.01 (0.63, 1.60) | 0.98 | N/A | N/A | 0.63 |
| | Formula | 1 trial | 69 | 1.15 (0.50, 2.64) | 0.73 | N/A | N/A | |
| Metabolic risk in childhood | BM | 2 trials | 206 | 0.85 (0.59, 1.22) | 0.38 | 0.12 | 0.03 | 0.12 |
| | Formula | 1 trial | 128 | 1.34 (0.83, 2.18) | 0.23 | N/A | N/A | |
| Metabolic risk in adolescence | BM | none | N/A | N/A | N/A | N/A | N/A | N/A |
| | Formula | 2 trials | 104 | 0.86 (0.66, 1.11) | 0.24 | 0.27 | 0.02 | |
| Metabolic risk at >3 years | BM | 2 trials | 206 | 0.85 (0.59, 1.22) | 0.38 | 0.12 | 0.03 | 0.51 |
| | Formula | 3 trials | 232 | 0.98 (0.76, 1.26) | 0.87 | 0.30 | 0.17 | |
| Cognitive scores in toddlers | BM | 6 trials | 541 | 0.68 (-2.17, 3.53) | 0.64 | 0.82 | 2.10 | 0.40 |
| | Formula | 5 trials | 704 | -0.25 (-2.20, 1.70) | 0.80 | 0.60 | 0.98 | |
| | PN+EN | 2 trials | 165 | 3.75 (-0.58, 8.08) | 0.09 | 0.49 | 4.84 | |
| Cognitive scores at >3 years | BM | 1 trial | 137 | -2.84 (-6.33, 0.65) | 0.11 | N/A | N/A | 0.69 |
| | Formula | 1 trial | 69 | -1.45 (-8.76, 5.86) | 0.69 | N/A | N/A | |

| Outcome | Subgroup | No. of trials | No. of participants | aRR or aMD (95% CI) | P for overall effect | P for heterogeneity | Tau² | P for subgroup interaction |
|---|---|---|---|---|---|---|---|---|
| Motor impairment in toddlers | BM | 6 trials | 541 | 0.94 (0.69, 1.27) | 0.67 | 0.89 | 0.02 | 0.64 |
| | Formula | 5 trials | 701 | 0.83 (0.56, 1.24) | 0.37 | 0.74 | 0.04 | |
| | PN+EN | 2 trials | 264 | 0.98 (0.73, 1.32) | 0.90 | 0.87 | 0.02 | |
| Motor scores in toddlers | BM | 6 trials | 541 | 2.32 (0.05, 5.06) | 0.046 | 0.87 | 23.14 | 0.64 |
| | Formula | 5 trials | 701 | 1.02 (-0.82, 2.85) | 0.28 | 0.006 | 0.86 | |
| | PN+EN | 2 trials | 264 | 0.28 (-4.00, 4.55) | 0.90 | 0.38 | 4.67 | |
| SBP in childhood (mmHg) | BM | 1 trial | 126 | 1.74 (-1.82, 5.29) | 0.34 | N/A | N/A | 0.47 |
| | Formula | 1 trial | 127 | 0.05 (-3.15, 3.24) | 0.98 | N/A | N/A | |
| SBP in adolescence (mmHg) | BM | None | N/A | N/A | N/A | N/A | N/A | N/A |
| | Formula | 2 trials | 101 | 1.13 (-2.52, 4.77) | 0.54 | 0.11 | 3.24 | |
| SBP at >3 years (mmHg) | BM | 1 trial | 126 | 1.74 (-1.82, 5.29) | 0.34 | N/A | N/A | 0.51 |
| | Formula | 3 trials | 228 | 0.52 (-1.86, 2.89) | 0.52 | 0.22 | 1.44 | |
| DBP in childhood (mmHg) | BM | 1 trial | 126 | -1.16 (-4.57, 2.24) | 0.50 | N/A | N/A | 0.83 |
| | Formula | 1 trial | 127 | -0.50 (-3.12, 2.12) | 0.71 | N/A | N/A | |
| | BM | None | N/A | N/A | N/A | N/A | N/A | N/A |

| Outcome | Subgroup | No. of trials | No. of participants | aRR or aMD (95% CI) | P for overall effect | P for heterogeneity | Tau² | P for subgroup interaction |
|---|---|---|---|---|---|---|---|---|
| DBP in adolescence (mmHg) | Formula | 2 trials | 101 | 0.51 (-2.29, 3.30) | 0.72 | 0.26 | 1.99 | |
| DBP at >3 years (mmHg) | BM | 1 trial | 126 | -1.16 (-4.57, 2.24) | 0.50 | N/A | N/A | 0.66 |
| | Formula | 3 trials | 228 | -0.14 (-2.04, 1.75) | 0.88 | 0.38 | 0.92 | |
| MAP in childhood (mmHg) | BM | 1 trial | 143 | -0.73 (-3.66, 2.21) | 0.62 | N/A | N/A | 0.82 |
| | Formula | 1 trial | 127 | -0.18 (-3.00, 2.64) | 0.90 | N/A | N/A | |
| MAP in adolescence (mmHg) | BM | None | N/A | N/A | N/A | N/A | N/A | N/A |
| | Formula | 2 trials | 99 | 0.27 (-2.68, 3.21) | 0.86 | 0.06 | 2.22 | |
| MAP at >3 years (mmHg) | BM | 1 trial | 143 | -0.73 (-3.66, 2.21) | 0.62 | N/A | N/A | 0.74 |
| | Formula | 3 trials | 226 | -0.05 (-2.07, 1.97) | 0.96 | 0.14 | 1.04 | |
| Triglyceride concentrations at >3 years (mmol/L) | BM | 1 trial | 77 | -0.12 (-0.22, -0.01) | 0.03 | N/A | N/A | 0.88 |
| | Formula | 2 trials | 78 | -0.09 (-0.41, 0.23) | 0.57 | 0.06 | 0.03 | |
| Cholesterol concentrations at >3 years (mmol/L) | BM | 1 trial | 78 | -0.12 (-0.45, 0.20) | 0.45 | N/A | N/A | 0.11 |
| | Formula | 2 trials | 78 | 0.25 (-0.14, 0.64) | 0.21 | 0.52 | 0.04 | |
| | BM | 1 trial | 77 | 0.02 (-0.12, 0.16) | 0.74 | N/A | N/A | 0.61 |

| Outcome | Subgroup | No. of trials | No. of participants | aRR or aMD (95% CI) | P for overall effect | P for heterogeneity | Tau² | P for subgroup interaction |
|---|---|---|---|---|---|---|---|---|
| HDL concentrations at >3 years (mmol/L) | Formula | 2 trials | 78 | -0.02 (-0.15, 0.11) | 0.78 | 0.92 | 0.004 | |
| LDL concentrations at >3 years (mmol/L) | BM | 1 trial | 77 | -0.08 (-0.36, 0.19) | 0.56 | N/A | N/A | 0.25 |
| | Formula | 2 trials | 77 | 0.12 (-0.16, 0.40) | 0.38 | 0.65 | 0.02 | |
| Blood glucose concentrations at >3 years (mmol/L) | BM | 1 trial | 71 | -0.02 (-0.30, 0.27) | 0.91 | N/A | N/A | 0.46 |
| | Formula | 2 trials | 78 | -0.13 (-0.28, 0.02) | 0.10 | 0.14 | 0.01 | |
| BMI in childhood (kg/m²) | BM | 2 trials | 206 | -0.26 (-0.71, 0.19) | 0.25 | 0.42 | 0.05 | 0.26 |
| | Formula | 1 trial | 127 | 0.14 (-0.47, 0.75) | 0.65 | N/A | N/A | |
| BMI in adolescence (kg/m²) | BM | none | N/A | N/A | N/A | N/A | N/A | N/A |
| | Formula | 2 trials | 103 | 0.14 (-1.27, 1.56) | 0.84 | 0.02 | 0.50 | |
| BMI at >3 years (kg/m²) | BM | 2 trials | 206 | -0.26 (-0.71, 0.19) | 0.25 | 0.42 | 0.05 | 0.32 |
| | Formula | 3 trials | 230 | 0.14 (-0.56, 0.84) | 0.70 | 0.01 | 0.13 | |
| BMI z-score in childhood | BM | 2 trials | 206 | -0.17 (-0.47, 0.12) | 0.25 | 0.72 | 0.02 | 0.37 |
| | Formula | 1 trial | 127 | 0.03 (-0.34, 0.39) | 0.88 | N/A | N/A | |

| Outcome | Subgroup | No. of trials | No. of participants | aRR or aMD (95% CI) | P for overall effect | P for heterogeneity | Tau² | P for subgroup interaction |
|---|---|---|---|---|---|---|---|---|
| BMI z-score in adolescence | BM | none | N/A | N/A | N/A | N/A | N/A | N/A |
| | Formula | 2 trials | 103 | -0.08 (-0.60, 0.44) | 0.76 | 0.01 | 0.07 | |
| BMI z-score at >3 years | BM | 2 trials | 206 | -0.17 (-0.47, 0.12) | 0.25 | 0.72 | 0.02 | 0.44 |
| | Formula | 3 trials | 230 | -0.03 (-0.33, 0.27) | 0.84 | 0.01 | 0.02 | |
| Fasting insulin at >3 years (pmol/L) | BM | 1 trial | 88 | 2.48 (-7.51, 12.47) | 0.62 | N/A | N/A | 0.46 |
| | Formula | 2 trials | 76 | -2.57 (-16.24, 11.11) | 0.71 | 0.32 | 47.06 | |

Abbreviation: BM: breast milk; SBP: systolic blood pressure; DBP diastolic blood pressure; MAP: mean arterial pressure; HDL: high-density lipoproteins; LDL: low-density lipoproteins; BMI: body mass index; aRR: adjusted relative risk; aMD: adjusted mean difference; NA, not applicable; BM: breast milk; PN: parenteral nutrition; EN: enteral nutrition
Relative risk and mean difference were adjusted for sex, gestational age and birthweight z-scores.

**Table S8. Subgroup analyse of different epochs**

| Outcome | Subgroup | No. of trials | No. of participants | aRR or aMD (95% CI) | P for overall effect | P for heterogeneity | $Tau^2$ | P for subgroup interaction |
|---|---|---|---|---|---|---|---|---|
| Cognitive impairment in toddlers | Before or in 2000 | 6 trials | 945 | 1.00 (0.75, 1.33) | 0.99 | 0.79 | | 0.70 |
| | After 2000 | 7 trials | 465 | 0.87 (0.65, 1.16) | 0.35 | 0.80 | | |
| Cognitive impairment at >3 years | Before or in 2000 | 1 trial | 69 | 1.15 (0.50, 2.64) | 0.73 | N/A | N/A | 0.63 |
| | After 2000 | 1 trial | 137 | 1.01 (0.63, 1.60) | 0.98 | N/A | N/A | |
| Metabolic risk in childhood | Before or in 2000 | 1 trial | 128 | 1.34 (0.83, 2.18) | 0.23 | N/A | N/A | 0.12 |
| | After 2000 | 2 trials | 206 | 0.85 (0.59, 1.22) | 0.38 | 0.12 | 0.03 | |
| Metabolic risk in adolescence | Before or in 2000 | 1 trial | 68 | 1.11 (0.73, 1.68) | 0.63 | N/A | N/A | 0.04 |
| | After 2000 | 1 trial | 36 | 0.59 (0.36, 0.97) | 0.04 | N/A | N/A | |
| Metabolic risk at >3 years | Before or in 2000 | 2 trials | 196 | 1.16 (0.85, 1.59) | 0.34 | 0.62 | 0.02 | 0.06 |
| | After 2000 | 3 trials | 242 | 0.78 (0.58, 1.05) | 0.09 | 0.25 | 0.02 | |
| Cognitive scores in toddlers | Before or in 2000 | 6 trials | 945 | 0.34 (-1.66, 2.34) | 0.40 | 0.83 | | 0.42 |
| | After 2000 | 7 trials | 465 | 1.62 (-0.80, 4.04) | 0.19 | 0.44 | | |
| Cognitive scores at >3 years | Before or in 2000 | 1 trial | 69 | -1.45 (-8.76, 5.86) | 0.69 | N/A | N/A | 0.69 |
| | After 2000 | 1 trial | 137 | -2.84 (-6.33, 0.65) | 0.11 | N/A | N/A | |
| Motor impairment in toddlers | Before or in 2000 | 6 trials | 943 | 0.87 (0.63, 1.21) | 0.62 | 0.48 | | 0.94 |
| | After 2000 | 7 trials | 463 | 0.84 (0.62, 1.14) | 0.25 | 0.99 | | |

| Outcome | Subgroup | No. of trials | No. of participants | aRR or aMD (95% CI) | P for overall effect | P for heterogeneity | Tau² | P for subgroup interaction |
|---|---|---|---|---|---|---|---|---|
| Motor scores in toddlers | Before or in 2000 | 6 trials | 943 | 1.34 (-0.37, 3.04) | 0.13 | 0.03 | | 0.69 |
| | After 2000 | 7 trials | 463 | 2.01 (-0.54, 4.57) | 0.12 | 0.87 | | |
| SBP in childhood (mmHg) | Before or in 2000 | 1 trial | 127 | 0.04 (-3.15, 3.24) | 0.65 | N/A | N/A | 0.46 |
| | After 2000 | 1 trial | 126 | 1.74 (-1.82, 5.29) | 0.34 | N/A | N/A | |
| SBP in adolescence (mmHg) | Before or in 2000 | 1 trial | 65 | 3.64 (-1.06, 8.35) | 0.13 | N/A | N/A | 0.11 |
| | After 2000 | 1 trial | 36 | -2.95 (-9.22, 3.32) | 0.35 | N/A | N/A | |
| SBP at >3 years (mmHg) | Before or in 2000 | 2 trials | 192 | 1.23 (-1.40, 3.85) | 0.36 | 0.23 | 2.25 | 0.89 |
| | After 2000 | 2 trials | 162 | 0.84 (-2.14. 3.82) | 0.56 | 0.24 | 2.25 | |
| DBP in childhood (mmHg) | Before or in 2000 | 1 trial | 127 | -0.50 (-3.12, 2.12) | 0.71 | N/A | N/A | 0.83 |
| | After 2000 | 1 trial | 126 | -1.16 (-4.57, 2.45) | 0.50 | N/A | N/A | |
| DBP in adolescence (mmHg) | Before or in 2000 | 1 trial | 65 | 1.72 (-1.87, 5.31) | 0.34 | N/A | N/A | 0.26 |
| | After 2000 | 1 trial | 36 | -1.07 (-6.22, 4.08) | 0.67 | N/A | N/A | |
| DBP at >3 years (mmHg) | Before or in 2000 | 2 trials | 192 | 0.28 (-1.80, 2.37) | 0.79 | 0.33 | 1.12 | 0.42 |
| | After 2000 | 2 trials | 162 | -1.07 (-3.86, 1.72) | 0.45 | 0.89 | 1.99 | |
| MAP in childhood (mmHg) | Before or in 2000 | 1 trial | 127 | -0.18 (-2.99, 2.64) | 0.90 | N/A | N/A | 0.82 |
| | After 2000 | 1 trial | 143 | -0.73 (-3.66, 2.21) | 0.62 | N/A | N/A | |
| MAP in adolescence (mmHg) | Before or in 2000 | 1 trial | 65 | 2.36 (-1.38, 6.11) | 0.21 | N/A | N/A | 0.05 |
| | After 2000 | 1 trial | 34 | -3.97 (-9.55, 1.61) | 0.16 | N/A | N/A | |

| Outcome | Subgroup | No. of trials | No. of participants | aRR or aMD (95% CI) | P for overall effect | P for heterogeneity | Tau² | P for subgroup interaction |
|---|---|---|---|---|---|---|---|---|
| MAP at >3 years (mmHg) | Before or in 2000 | 2 trials | 192 | 0.66 (-1.57, 2.88) | 0.56 | 0.32 | 0.44 | 0.26 |
| | After 2000 | 2 trials | 177 | -1.15 (-3.67, 1.36) | 0.37 | 0.47 | 1.61 | |
| Triglyceride concentration in childhood (mmol/L) | Before or in 2000 | none | N/A | N/A | N/A | N/A | N/A | N/A |
| | After 2000 | 1 trial | 77 | -0.12 (-0.22, -0.01) | 0.03 | N/A | N/A | |
| Triglyceride concentration in adolescence (mmol/L) | Before or in 2000 | 1 trial | 51 | 0.09 (-0.11, 0.29) | 0.37 | N/A | N/A | 0.07 |
| | After 2000 | 1 trial | 27 | -0.54 (-1.49, 0.10) | 0.25 | N/A | N/A | |
| Triglyceride concentration at >3 years (mmol/L) | Before or in 2000 | 1 trial | 51 | 0.09 (-0.11, 0.29) | 0.37 | N/A | N/A | 0.06 |
| | After 2000 | 2 trials | 104 | -0.23 (-0.45, -0.00) | 0.05 | 0.12 | 0.03 | |
| Cholesterol concentration in childhood (mmol/L) | Before or in 2000 | none | N/A | N/A | N/A | N/A | N/A | N/A |
| | After 2000 | 1 trial | 78 | -0.12 (00.45, 0.20) | 0.45 | N/A | N/A | |
| Cholesterol concentration in adolescence (mmol/L) | Before or in 2000 | 1 trial | 51 | 0.18 (-0.22, 0.58) | 0.37 | N/A | N/A | 0.42 |
| | After 2000 | 1 trial | 27 | 0.64 (-0.29, 1.56) | 0.17 | N/A | N/A | |
| Cholesterol concentration at >3 years (mmol/L) | Before or in 2000 | 1 trial | 51 | 0.18 (-0.22, 0.58) | 0.37 | N/A | N/A | 0.61 |
| | After 2000 | 2 trials | 105 | 0.05 (-0.27, 0.36) | 0.77 | 0.12 | 0.04 | |
| HDL concentration in childhood (mmol/L) | Before or in 2000 | none | N/A | N/A | N/A | N/A | N/A | N/A |
| | After 2000 | 1 trial | 77 | 0.02 (-0.12, 0.16) | 0.74 | N/A | N/A | |

| Outcome | Subgroup | No. of trials | No. of participants | aRR or aMD (95% CI) | P for overall effect | P for heterogeneity | Tau² | P for subgroup interaction |
|---|---|---|---|---|---|---|---|---|
| HDL concentration in adolescence (mmol/L) | Before or in 2000 | 1 trial | 51 | -0.03 (-0.18, 0.12) | 0.70 | N/A | N/A | 0.97 |
| | After 2000 | 1 trial | 27 | -0.02 (-0.31, 0.27) | 0.90 | N/A | N/A | |
| HDL concentration at >3 years (mmol/L) | Before or in 2000 | 1 trial | 51 | -0.03 (-0.18, 0.12) | 0.70 | N/A | N/A | 0.76 |
| | After 2000 | 2 trials | 104 | 0.01 (-0.11, 0.13) | 0.83 | 0.86 | 0.004 | |
| LDL concentration in childhood (mmol/L) | Before or in 2000 | none | N/A | N/A | N/A | N/A | N/A | N/A |
| | After 2000 | 1 trial | 77 | -0.08 (-0.36, 0.20) | 0.56 | N/A | N/A | |
| LDL concentration in adolescence (mmol/L) | Before or in 2000 | 1 trial | 50 | 0.16 (-0.21, 0.53) | 0.38 | N/A | N/A | 0.85 |
| | After 2000 | 1 trial | 27 | 0.27 (-0.16, 0.69) | 0.21 | N/A | N/A | |
| LDL concentration at >3 years (mmol/L) | Before or in 2000 | 1 trial | 50 | 0.16 (-0.21, 0.53) | 0.38 | N/A | N/A | 0.35 |
| | After 2000 | 2 trials | 104 | -0.03 (-0.25, 0.20 ) | 0.82 | 0.58 | 0.02 | |
| Blood glucose concentration in childhood (mmol/L) | Before or in 2000 | none | N/A | N/A | N/A | N/A | N/A | N/A |
| | After 2000 | 1 trial | 71 | -0.02 (-0.30, 0.27) | 0.91 | N/A | N/A | |
| Blood glucose concentration in adolescence (mmol/L) | Before or in 2000 | 1 trial | 51 | -0.04 (-0.21, 0.14) | 0.67 | N/A | N/A | 0.13 |
| | After 2000 | 1 trial | 27 | -0.34 (-0.67, -0.02) | 0.04 | N/A | N/A | |
| Blood glucose concentration at >3 years (mmol/L) | Before or in 2000 | 1 trial | 51 | -0.04 (-0.21, 0.14) | 0.67 | N/A | N/A | 0.89 |
| | After 2000 | 2 trials | 98 | -0.09 (-0.31, 0.14) | 0.45 | 0.28 | 0.05 | |
| | Before or in 2000 | 1 trial | 127 | 0.14 (-0.47, 0.75) | 0.65 | N/A | N/A | 0.26 |

| Outcome | Subgroup | No. of trials | No. of participants | aRR or aMD (95% CI) | P for overall effect | P for heterogeneity | Tau² | P for subgroup interaction |
|---|---|---|---|---|---|---|---|---|
| BMI in childhood (kg/m²) | After 2000 | 2 trials | 206 | -0.26 (-0.71, 0.19) | 0.25 | 0.42 | 0.05 | |
| BMI in adolescence (kg/m²) | Before or in 2000 | 1 trial | 67 | 1.40 (-0.48, 3.27) | 0.14 | N/A | N/A | 0.02 |
| | After 2000 | 1 trial | 36 | -2.49 (-4.51, -0.48) | 0.02 | N/A | N/A | |
| BMI at >3 years (kg/m²) | Before or in 2000 | 2 trials | 194 | 0.57 (-0.17, 1.32) | 0.13 | 0.13 | 0.14 | 0.01 |
| | After 2000 | 3 trials | 242 | -0.52 (-1.00, -0.04) | 0.03 | 0.03 | 0.06 | |
| BMI z-score in childhood | Before or in 2000 | 1 trial | 127 | 0.03 (-0.34, 0.39) | 0.88 | N/A | N/A | 0.37 |
| | After 2000 | 2 trials | 206 | -0.17 (-0.47, 0.12) | 0.25 | 0.72 | 0.03 | |
| BMI z-score in adolescence | Before or in 2000 | 1 trial | 67 | 0.47 (-0.17, 1.11) | 0.15 | N/A | N/A | 0.006 |
| | After 2000 | 1 trial | 36 | -1.20 (-2.08, -0.31) | 0.01 | N/A | N/A | |
| BMI z-score at >3 years | Before or in 2000 | 2 trials | 194 | 0.17 (-0.15, 0.49) | 0.30 | 0.23 | 0.03 | 0.03 |
| | After 2000 | 3 trials | 242 | -0.29 (-0.57, -0.01) | 0.04 | 0.14 | 0.02 | |
| Fasting insulin in childhood (pmol/L) | Before or in 2000 | none | N/A | N/A | N/A | N/A | N/A | N/A |
| | After 2000 | 1 trial | 88 | 2.48 (-7.51, 12.47) | 0.62 | N/A | N/A | |
| Fasting insulin in adolescence (pmol/L) | Before or in 2000 | 1 trial | 49 | 2.29 (-15.09, 19.68) | 0.79 | N/A | N/A | 0.34 |
| | After 2000 | 1 trial | 27 | -24.74 (-47.54, -1.94) | 0.03 | N/A | N/A | |
| Fasting insulin at >3 years (pmol/L) | Before or in 2000 | 1 trial | 49 | 2.29 (-15.09, 19.68) | 0.79 | N/A | N/A | 0.90 |
| | After 2000 | 2 trials | 115 | -0.87 (-9.79, 8.06) | 0.85 | 0.09 | 47.06 | |

| Outcome | Subgroup | No. of trials | No. of participants | aRR or aMD (95% CI) | P for overall effect | P for heterogeneity | Tau² | P for subgroup interaction |
|---|---|---|---|---|---|---|---|---|
| IGF-I in adolescence (nmol/L) | Before or in 2000 | 1 trail | 49 | 3.45 (-8.11, 15.01) | 0.55 | N/A | N/A | 0.87 |
| | After 2000 | 1 trial | 28 | -8.41 (-21.91, 5.09) | 0.21 | N/A | N/A | |

Abbreviation: SBP: systolic blood pressure; DBP diastolic blood pressure; MAP: mean arterial pressure; HDL: high-density lipoproteins; LDL: low-density lipoproteins; BMI: body mass index; aRR: adjusted relative risk; aMD: adjusted mean difference; NA, not applicable

**Table S9. Search strategies**

| **Embase from 1980 to 2019 April 01** | |
|---|---|
| # | Search strategies |
| 1 | exp prematurity/ |
| 2 | exp low birth weight/ |
| 3 | exp small for date infant/ |
| 4 | exp very low birth weight/ |
| 5 | (prematur* adj2 infant*).tw. |
| 6 | (prematur* adj2 newborn*).tw. |
| 7 | (prematur* adj2 neonate*).tw. |
| 8 | preterm.tw. |
| 9 | low birth weight.tw. |
| 10 | low birthweight.tw. |
| 11 | VLBW.tw. |
| 12 | LBW.tw. |
| 13 | ELBW.tw. |
| 14 | small for gestation*.tw. |
| 15 | SGA.tw. |
| 16 | (less than adj6 g).tw. |
| 17 | (less than adj3 32 weeks).tw. |
| 18 | birth weight below.tw. |
| 19 | (gestation* adj2 less than).tw. |
| 20 | or/1-19 |
| 21 | exp breast feeding/ |
| 22 | exp infant nutrition/ |
| 23 | exp protein intake/ |
| 24 | exp dietary supplement/ |
| 25 | exp omega 3 fatty acid/ct, ad, dt, ig, pa [Clinical Trial, Drug Administration, Drug Therapy, Intragastric Drug Administration, Parenteral Drug Administration] |
| 26 | exp arachidonic acid/ae, ct, ad, dt, ig, pa, th [Adverse Drug Reaction, Clinical Trial, Drug Administration, Drug Therapy, Intragastric Drug Administration, Parenteral Drug Administration, Therapy] |
| 27 | exp unsaturated fatty acid/ct, dt, pa, th [Clinical Trial, Drug Therapy, Parenteral Drug Administration, Therapy] |
| 28 | exp fat intake/ae, ad, dt [Adverse Drug Reaction, Drug Administration, Drug Therapy] |
| 29 | exp enteric feeding/ |
| 30 | exp parenteral nutrition/ |
| 31 | exp artificial milk/ |
| 32 | exp breast milk/ |
| 33 | exp fortified food/ |
| 34 | exp elemental diet/ |
| 35 | exp baby food/ |
| 36 | (breast milk or human milk).tw. |
| 37 | formula.tw. |
| 38 | PUFA supplement*.tw. |
| 39 | feed* regimen*.tw. |
| 40 | (protein* adj2 concentration*).tw. |
| 41 | probiotic$.tw. |
| 42 | parenteral*.tw. |
| 43 | enteral*.tw. |
| 44 | maternal milk.tw. |
| 45 | multinutrient supplement*.tw. |

| | |
|---|---|
| 46 | (breast fed or breastfed).tw. |
| 47 | prebiotic*.tw. |
| 48 | diet* supplement*.tw. |
| 49 | nutrient enriched.tw. |
| 50 | Docosahexaenoic Acid*.tw. |
| 51 | arachidonic acid*.tw. |
| 52 | (glutamine adj2 supplement*).tw. |
| 53 | (taurine adj2 supplement*).tw. |
| 54 | (calcium adj2 supplement*).tw. |
| 55 | palm olein.tw. |
| 56 | palmitic acid.tw. |
| 57 | (fortification or fortified).tw. |
| 58 | fatty acids.tw. |
| 59 | supplement* feed*.tw. |
| 60 | complementary feed*.tw. |
| 61 | nutrition*.tw. |
| 62 | Hydrolysed liquid.tw. |
| 63 | Hydrolyzed liquid.tw. |
| 64 | gamma-linoleic acid.tw. |
| 65 | (diet* adj3 protein*).tw. |
| 66 | or/21-65 |
| 67 | 20 and 66 |
| 68 | Clinical Trial/ |
| 69 | Randomized Controlled Trial/ |
| 70 | exp randomization/ |
| 71 | Single Blind Procedure/ |
| 72 | Double Blind Procedure/ |
| 73 | Crossover Procedure/ |
| 74 | Placebo/ |
| 75 | Randomi?ed controlled trial$.tw. |
| 76 | Rct.tw. |
| 77 | random allocation.tw. |
| 78 | randomly.tw. |
| 79 | randomly allocated.tw. |
| 80 | allocated randomly.tw. |
| 81 | (allocated adj2 random).tw. |
| 82 | Single blind$.tw. |
| 83 | Double blind$.tw. |
| 84 | ((treble or triple) adj blind$).tw. |
| 85 | placebo$.tw. |
| 86 | prospective study/ |
| 87 | or/68-86 |
| 88 | case study/ |
| 89 | case report.tw. |
| 90 | abstract report/ or letter/ |
| 91 | or/88-90 |
| 92 | 87 not 91 |
| 93 | 67 and 92 |

**Table S10. Definitions for primary outcome of metabolic risk.**

| Measurement | Guideline/ Equipment | Age | Abnormal | Notes |
|---|---|---|---|---|
| Size for gestation at birth | INTERGROWTH 21 Charts [1] | ≤6 months | ≤10th centile vs >10th centile | INTERGROWTH 21 charts for babies younger than 6 months [1] |
| Overweight/obese | WHO Growth Charts [2, 3] | <5 years [2] | Overweight: weight-for-height greater than 2 SD above WHO Child Growth Standards median; Obesity: weight-for-height greater than 3 SD above the WHO Child Growth Standards median. | Charts and tables: WHO child growth standards for children aged under 5 years [2] |
| | | 5-19 years [3] | Overweight: BMI-for-age greater than 1 SD above the WHO Growth Reference median; Obesity: greater than 2 SD above the WHO Growth Reference median. | Charts and tables: WHO growth reference for children aged between 5-19 years [2] |
| Waist Circumference | NHANES 2011-2014 [4] | 2- 60 years | ≥90th percentile [5] | |
| Fat mass (FM) | DXA-NHANES [6] | ≥8 years | Fat Mass Index (kg/m$^2$) classification ranges for sex | |
| | BIA [7] | 5-18 years | ≥85th percentile (%FM) | |
| | ADP- BodPod [7] | 5-18 years | ≥85th percentile (%FM) | |
| | ADP- PedPod [8] | 0·5-24 months | %FM greater than 1 SD above the reference mean | |
| | Skinfolds- NHES II, NHES III, NHANES I, NHANES II and NHANES III [9] | 1·5-19 years | ≥85th percentile [9] | |
| | Multicomponent model | 0·5-24 month [8] | %FM greater than 1 SD above the reference mean | |
| | | 5-20 years [10] | FM greater than 1 SD above the reference mean | Fat mass reference data for males and females by z-score or percentile [10] |

| Measurement | Guideline/ Equipment | Age | Abnormal | Notes |
|---|---|---|---|---|
| Blood pressure | NHBPEP [11] | 1 to 17 years | ≥90th percentile [5] (age, sex and height specific)<br>Charts and tables: WHO Child growth standards for length/height | Compared with Jackson LV 2007 [12], although the NHBPEP is older, it contains the appropriate age range and reported the actual numbers at each cut point. |
| Triglycerides | NHANES III, NHANES 1999–2004, Bogalusa, Muscatine, Fels, and Princeton [13] | 4-18 years | ≥90th percentile [14] | Compared to NHANES III, NCEP, and NGHS, this includes a wider age range. |
| | NHANES | >18 years | ≥150 mg/dL (8·3 mmol/L) [13] | |
| HDL-C | NHANES III, NHANES 1999–2004, Bogalusa, Muscatine, Fels, and Princeton [13] | 4-18 years | ≤10th percentile [14] | Compared to NHANES III, NCEP, and NGHS, this includes a wider age range. |
| | NHANES [13] | >18 years | <40 mg/dL (2·2 mmol/L) [13] for male | |
| | | | <50 mg/dL (2·8 mmol/L) [13] for female | |
| LDL-C | NHANES III, NHANES 1999–2004, Bogalusa, Muscatine, Fels, and Princeton [13] | 4-18 years | ≥90th percentile [13] | Compared to NHANES III, this includes a wider age range. |
| | NCEP ATP III | >18 years | >130 mg/dL (7·2 mmol/L) [13] | |
| Fasting plasma glucose concentration | ADA criterion [15] (increased risk for diabetes or prediabetes) | | FPG ≥100 mg/dL (5·6 mmol/L) | |
| Impaired glucose tolerance | ADA criterion [15] (increased risk for diabetes or prediabetes) | | 2 hours post meal glucose ≥140mg/dL (7·8 mmol/L) during a 75g oral glucose tolerance test | |

**References:**


1. Villar J, Cheikh Ismail L, Victora CG, Ohuma EO, Bertino E, Altman DG, et al. International standards for newborn weight, length, and head circumference by gestational age and sex: the Newborn Cross-Sectional Study of the INTERGROWTH-21st Project. Lancet. 2014;384(9946):857-68.

2. World Health Organization. Assessing growth using the WHO Growth Charts Geneva, Switzerland: World Health Organization; [cited 2018 March 19]. Available from: https://www.cdc.gov/nccdphp/dnpao/growthcharts/who/using/assessing_growth.htm

3. World Health Organization. Growth reference 5-19 years. Geneva, Switzerland: World Health Organization; [updated January 16, 2019; cited 2018 March 19]. Available from: https://www.who.int/growthref/who2007_bmi_for_age/en/

4. Fryar CD, Gu Q, Ogden CL, Flegal KM. Anthropometric reference data for children and adults: United States, 2011-2014. Vital Health Stat 3. 2016(39):1-46.

5. Zimmet P, George K, Alberti MM, Kaufman F, Tajima N, Silink M, et al. The metabolic syndrome in children and adolescents - an IDF consensus report. Pediatr Diabetes. 2007;8(5):299-306.

6. Kelly TL, Wilson KE, Heymsfield SB. Dual energy X-ray absorptiometry body composition reference values from NHANES. PLoS One. 2009;4(9).

7. McCarthy HD, Cole TJ, Fry T, Jebb SA, Prentice AM. Body fat reference curves for children. Int J Obes. 2006;30(4):598-602.

8. Butte NF, Hopkinson JM, Wong WW, Smith EO, Ellis KJ. Body composition during the first 2 years of life: An updated reference. Pediatr Res. 2000;47(5):578-85.

9. Addo OY, Himes JH. Reference curves for triceps and subscapular skinfold thicknesses in US children and adolescents. Am J Clin Nutr. 2010;91(3):635-42.

10. Wells JC, Williams JE, Chomtho S, Darch T, Grijalva-Eternod C, Kennedy K, et al. Body-composition reference data for simple and reference techniques and a 4-component model: a new UK reference child. Am J Clin Nutr. 2012;96(6):1316-26.

11. Falkner B, Daniels SR, Loggie JMH, Horan MJ, Prineas RJ, Rosner B, et al. Update on the 1987 task force report on high blood pressure in children and adolescents: a working group report from the National High Blood Pressure Education Program. Pediatrics. 1996;98(4):649-58.

12. Jackson LV, Thalange NKS, Cole TJ. Blood pressure centiles for Great Britain. Arch Dis Child. 2007;92(4):298-303.

13. Cook S, Auinger P, Huang TT. Growth curves for cardio-metabolic risk factors in children and adolescents. J Pediatr. 2009;155(3):S6 e15-26.

14. Hickman TB, Briefel RR, Carroll MD, Rifkind BM, Cleeman JI, Maurer KR, et al. Distributions and trends of serum lipid levels among United States children and adolescents ages 4-19 years: Data from the third National Health and Nutrition Examination Survey. Prev Med. 1998;27(6):879-90.

15. ADA criterion. 2. Classification and Diagnosis of Diabetes Diabetes Care American Diabetes Association; 2015 [updated 2015 Jan; cited 2017 Nov 27]; [American Diabetes Association ]. Available from: http://care.diabetesjournals.org/content/38/Supplement_1/S8

**Table S11. Definitions for secondary outcomes.**

| Term | Classification | Definition | Note |
|---|---|---|---|
| Cerebral palsy | | 1. Cerebral palsy is a physical disability that affects movement and posture.<br>Any definition that includes the following five key elements:<br>(1) is an umbrella term for a group of disorders<br>(2) is a condition that is permanent but not unchanging<br>(3) involves a disorder of movement and/or posture and of motor function<br>(4) is due to a non-progressive interference, lesion or abnormality, and<br>(5) the interference, lesion or abnormality originates in the immature brain<br>2. As defined by investigators | Australian cerebral palsy register report - CP Register.[1] |
| Severity of cerebral palsy | GMFCS Level I | Children walk at home, school, outdoors and in the community. They can climb stairs without the use of a railing. Children perform gross motor skills such as running and jumping, but speed, balance and coordination are limited. | Gross Motor Function Classification System (GMFCS).[2] |
| | GMFCS Level II | Children walk in most settings and climb stairs holding onto a railing. They may experience difficulty walking long distances and balancing on uneven terrain, inclines, in crowded areas or confined spaces.<br>Children may walk with physical assistance, a handheld mobility device or used wheeled mobility over long distances. Children have only minimal ability to perform gross motor skills such as running and jumping. | |
| | GMFCS Level III | Children walk using a hand-held mobility device in most indoor settings. They may climb stairs holding onto a railing with supervision or assistance. Children use wheeled mobility when traveling long distances and may self-propel for shorter distances. | |
| | GMFCS Level IV | Children use methods of mobility that require physical assistance or powered mobility in most settings. They may walk for short distances at home with physical assistance or use powered mobility or a body support walker when positioned. At school, outdoors and in the community children are transported in a manual wheelchair or use powered mobility. | |
| | GMFCS Level V | Children are transported in a manual wheelchair in all settings. Children are limited in their ability to maintain antigravity head and trunk postures and control leg and arm movements. | |
| Developmental delay or intellectual impairment | Mild | A score on scale from 2 SD to <1 SD below test mean. | Scores were obtained relative to the mean and SD for the normal birthweight population.[3] |
| | Moderate | A score on scale from 3 SD to <2 SD below test mean. | |
| | Severe | A score on scale 3 SD below test mean. | |
| Visual impairment | None | Presenting visual acuity 6/18 or better in the better eye. | WHO Definition of visual impairment.[4] |
| | Moderate/ low vision | Can see a toy and able to follow a toy. Presenting visual acuity worse than 6/18, equal to or better than 6/60 in the better eye in the better eye. | |

| Term | Classification | Definition | Note |
|---|---|---|---|
| | Severe/ no useful vision | Able to see light or gross movement up close (within 40cm). Presenting visual acuity worse than 6/60, equal to or better than 1/60 in the better eye. | Visual Standards- Aspects and Ranges of Vision Loss.[5] |
| | Blindness/ no light perception | No useful vision. Presenting visual acuity worse than 1/60 in the better eye or no light perception. | |
| | Legal blindness | Medically diagnosed central visual acuity of 20/200 (6/60) or less in the better eye with the best possible correction, and/or a visual field of 20 degrees or less. | American Foundation for the Blind.[6] |
| Hearing impairment (Classification 1) | None | None diagnosed. | WHO Grades of hearing impairment- Prevention of blindness and deafness.[7] |
| | Mild | Hearing level in decibels: 26-40dB<br>A child with this level of hearing loss will have trouble hearing and understanding soft speech, speech from a distance or speech against a background of noise. | |
| | Moderate | Hearing level in decibels: 41-60db<br>A child with this level of hearing loss will have difficulty hearing regular speech, even at close distance. | |
| | Severe | Hearing level in decibels: 61-80dB<br>A child with this level of hearing loss may only hear very loud speech or loud sounds in the environment, such as a fire truck siren or a door slamming. Most conversational speech is not heard. | |
| | Profound | Hearing level in decibels: over 81dB<br>A child with this level of hearing loss may perceive loud sounds as vibrations. | |
| Motor dysfunction | mild impairment | Test score between 5th and 15th centile on the Movement ABC / A score from 2 SD to <1 SD below the population mean on the BOTMP. | Movement Assessment Battery for Children (Movement ABC) |
| | moderate to severe impairment | Test score less than 5th centile on the Movement ABC / more than 2 SD below the population mean on the BOTMP. | Bruininks–Oseretsky Test of Motor Proficiency (BOTMP)[8] |
| School performance | Defined by teachers based on their observation and academic scores;<br>at or above vs below expected performance/level for age. | | Poor school performance[9] |
| Growth Z-scores | WHO Growth Charts. | | Charts and tables: WHO child growth standards for children[10] |


References:

1. The Austrialian Cerebral Palsy Register Group. Austrialian cerebral palsy register report 2016 Cerebral Palsy Alliance. Available from: https://www.cpregister.com/pubs/pdf/ACPR-Report_Web_2016.pdf
2. Palisano RJ, Rosenbaum P, Bartlett D, Livingston MH. Content validity of the expanded and revised Gross Motor Function Classification System. Dev Med Child Neurol. 2008;50(10):744-50.
3. Doyle LW, Roberts G, Anderson PJ, Stu VIC. Outcomes at age 2 years of infants < 28 weeks' gestational age born in Victoria in 2005. J Pediatr. 2010;156(1):49-U84.
4. IAPB, VISION 2020, WHO. Global Initiative for the elimination of avoidable blindness- Action plan 2006-2011. Available from: http://www.who.int/blindness/Vision2020_report.pdf

5. International Council of Ophthalmology. Visual standards- Aspects and ranges of vision loss with emphasis on population surveys 2002. Available from: http://www.icoph.org/downloads/visualstandardsreport.pdf
6. American Foundation for the Blind. Key definitions of statistical terms- vision terms 2017. Available from: http://www.afb.org/info/blindness-statistics/key-definitions-of-statistical-terms/25
7. WHO. Prevention of blindness and deafness- hearing loss grades Available from: http://www.who.int/pbd/deafness/hearing_impairment_grades/en/
8. Williams J, Lee KJ, Anderson PJ. Prevalence of motor-skill impairment in preterm children who do not develop cerebral palsy: a systematic review. Dev Med Child Neurol. 2010;52(3):232-7.
9. Siqueira CM, Gurgel-Giannetti J. Poor school performance: an updated review. Rev Assoc Med Bras (1992). 2011;57(1):78-87.
10. World Health Organization. Assessing growth using the WHO Growth Charts Geneva, Switzerland: World Health Organization; [cited 2018 March 19]. Available from: https://www.cdc.gov/nccdphp/dnpao/growthcharts/who/using/assessing_growth.htm
